# Global microplastic pollution at levels harmful to marine life

**DOI:** 10.1007/s11356-025-37149-x

**Published:** 2025-11-24

**Authors:** Mark E. M. Walton, Maria Wedinger, Victoria Mason, Maria Kristina O. Paler, Evelyn B. Taboada, Martin W. Skov, Jan G. Hiddink

**Affiliations:** 1 School of Ocean Sciences, Bangor University, Menai Bridge, UK; 2 Royal Netherlands Institute for Sea Research Division Yerseke: Koninklijk Nederlands Instituut Voor Onderzoekder , Zee Vestiging Yerseke, Yerseke, Kingdom of the Netherlands; 3 University of San Carlos, Cebu City, Philippines

**Keywords:** Pollution, Marine plastic litter, Mangrove, Blue Carbon, Biological impacts

## Abstract

Poor waste management has resulted in marine plastic litter increasing worldwide, and microplastics (MP) are now detected in all marine habitats. To better understand the distribution and ecological implications of MPs, we quantify the abundance of MP in all major marine habitats through a meta-analysis of data collected by systematic review. We extracted MP densities from 334 studies covering all major water column, intertidal, inshore and offshore seabed habitats from all continents. MPs are ubiquitous, and are present in all habitats in all continents with an average (± 95%CI) global density of 37,921 ± 13,925 particles m^−2^ or 0.011 kg m^−2^. MP densities in sediments are at least an order of magnitude higher than in the water column. Mean MP densities are highest in Asia, in tropical regions, and in mangroves. Although sedimentary habitats such as mangroves, saltmarshes, and seagrasses are often considered potential MP sinks due to high sedimentation rates, our analysis revealed that “Blue Carbon” habitats do not consistently exhibit elevated MP densities. We found that MP densities significantly increased with decreasing minimum detectable particle size. When we rescaled MP abundance to a minimum particle size of 0.01 mm to reduce the bias, this significantly increased the estimate of mean global MP density to 57,953  ±  17,442 particles m^−2^. Scaling our estimated MP densities for each habitat to a global estimate of total marine MP that exceeds 1.5 billion tonnes, far surpassing expectations based on historical plastic production. This discrepancy suggests that published MP studies may disproportionately sample polluted sites, underrepresenting broader environmental conditions. However, in the thousands of locations sampled in this meta-analysis, the mean MP densities are high enough to expect fundamental biological processes, such as growth and reproduction, of marine fauna to be significantly negatively impacted.

**Supplementary Information:**

The online version contains supplementary material available at 10.1007/s11356-025-37149-x.

## Introduction

The continuous rise in plastic production and poor waste management (Deng et al. 2021; Villarrubia-Gómez et al. 2018) has resulted in increasing amounts of plastic litter that impact marine organisms and habitats (Deng et al. 2021; Mason et al. 2022). Addressing marine plastic pollution is challenging due to the diversity of plastic types and sources from which they originate (Haward 2018). The exact amounts of plastic that enter and persist in the marine environment are debated (e.g., Eriksen et al. 2014; Worm et al. 2017; Wright and Kelly 2017) and the sheer volume increases the difficulty in managing marine plastic litter appropriately (Schwarz et al. 2019).

Since the 1950s, global plastic production has increased exponentially, reaching an estimated 10 billion metric tonnes by 2021 (Geyer et al. 2017; Plastics_Europe 2018, 2022), resulting in 6.3 billion tonnes of plastic waste in landfills and the natural environment (Brooks et al. 2018; Geyer et al. 2017). The durability of plastics, once considered a technological advantage, now poses a significant ecological threat. Degradation rates vary widely, from just two years for single-use items like bags and bottles to over 5000 years for industrial-grade polymers such as HDPE pipes (Chamas et al. 2020). While degradation is mostly slow, the bulk of plastic waste consists of packaging materials discarded within a year of production (Geyer et al. 2017; Plastics_Europe 2022) many of which degrade more rapidly. This suggests that a substantial fraction of historic plastic waste has already fragmented into microplastics (MPs), a form increasingly found in marine environments.

Microplastics originate from the breakdown of larger plastic debris through thermo-oxidative, hydrolytic, biological, mechanical or photochemical degradation processes (Reisser et al. 2013; Webb et al. 2012) and are classified by size as macroplastic (> 20 mm diameter), mesoplastic (5–20 mm), microplastic (0.001–5 mm), and nanoplastic (< 1 µm) (Bergmann et al. 2015; Napper and Thompson 2020). MPs are dispersed throughout the marine environment based on polymer density and hydrodynamic conditions (Schwarz et al. 2019). Buoyant polymers such as polyethylene (PE) and polypropylene (PP) can travel long distances across ocean currents (Galgani et al. 2021), in contrast to dense polymers like polyvinyl chloride (PVC), polyethylene terephthalate (PET), and polyamide (PA) which tend to sink quickly accumulating in coastal sediments (Horton and Dixon 2018). The majority of land-based plastic litter is retained in coastal environments (Harris 2020). Differences in plastic degradation rates across marine habitats, driven by environmental conditions, polymer type, and biological activity, directly influence MP accumulation, with slower degradation contributing to higher densities in sedimentary and vegetated coastal areas (Chamas et al. 2020).

Coastal environments, particularly those adjacent to urban centers and river mouths, are disproportionately affected by plastic pollution (Harris 2020; Islam and Tanaka 2004). These areas receive high inputs of land-based plastic waste and often act as major sinks for MPs due to their sediment-trapping characteristics and proximity to pollution sources (Thushari and Senevirathna 2020). Among coastal systems, vegetated “Blue Carbon” habitats such as mangroves, saltmarshes, and seagrasses are especially vulnerable. These habitats play crucial roles in carbon sequestration, sediment stabilization, coastal protection and as nursery and feeding grounds for many commercially important fished species (Himes-Cornell et al. 2018; Walton et al. 2021, 2006). Their dense vegetation promotes the deposition of suspended particles, including MPs, making them potential hotspots for plastic accumulation (Cappa et al. 2023; Carmen 2021; Cozzolino et al. 2020; Paler et al. 2022) and therefore potentially impacting these species at one of the most vulnerable stages in their life cycle.

Despite their ecological importance, Blue Carbon habitats have received relatively little attention in global assessments of MP pollution. Most studies have focused on sandy beaches or open water, overlooking the sediment-rich environments where MPs are likely to concentrate. This gap in knowledge is concerning, given the growing evidence that MPs can negatively impact marine organisms. MPs have been shown to cause physical damage such as intestinal blockage and laceration, physiological stress through the release of additives and adsorbed toxins, and disruption of biological processes including growth, reproduction, and feeding (Andrady 2017; Berlino et al. 2021, 2023; Mason et al. 2022; Salerno et al. 2021). Generally, seawater promotes faster biofilm formation and supports greater bacterial diversity compared to freshwater systems (Harrison et al. 2018). Marine biofilms frequently include opportunistic pathogens such as Vibrio spp., reinforcing the role of MPs as vectors for disease transmission across ecosystems (Chandra et al. 2025; Frère et al. 2018; Gaylarde et al. 2023). In addition, biofilm development can modify the buoyancy and transport behavior of MPs, enhancing their ecological persistence and spatial reach (Yu et al. 2025).

While laboratory studies have demonstrated the harmful effects of MPs on marine fauna (Berlino et al. 2023; Mason et al. 2022), it remains unclear whether environmental concentrations have reached levels sufficient to cause these impacts in situ. This uncertainty is compounded by methodological inconsistencies across studies, particularly in sampling techniques and detection thresholds. The abundance of MPs is known to increase exponentially with decreasing particle size, meaning that studies with higher detection limits may significantly underestimate true MP densities (Hidalgo-Ruz et al. 2012). Moreover, site selection bias, such as sampling near urban centers, can skew global estimates, as these locations tend to exhibit higher MP concentrations.

To address these gaps, this study presents a comprehensive meta-analysis of MP densities across all major marine habitats, continents, and climatic zones. We systematically reviewed 334 studies encompassing intertidal, inshore, offshore, and water column environments. Our objectives were to (1) quantify MP densities globally, (2) identify key sinks and hotspots, (3) assess the influence of methodological biases, and (4) evaluate whether current environmental concentrations are likely to impair biological processes in marine fauna.

We hypothesize that Blue Carbon habitats accumulate higher MP densities than adjacent non-vegetated systems due to their sediment-trapping structures and proximity to pollution sources. To test this, we conducted a sensitivity analysis examining the effects of sampling location, detection method, and minimum detectable particle size on reported MP densities. We also reanalysed data from Mason et al. (2022) to determine whether observed environmental concentrations align with those shown to cause biological harm in experimental settings.

This study provides the first global synthesis of MP densities across diverse marine habitats and offers critical insights into the ecological risks posed by MP pollution. By identifying patterns in MP distribution and evaluating their potential impacts, we aim to inform future research, guide policy interventions, and support efforts to improve plastic waste management worldwide.

## Methods

The habitats reviewed here include the major intertidal and subtidal sedimentary and water column habitats. The targeted habitats included coastal and offshore water, the Blue Carbon (BC) habitats (mangrove forest, seagrass beds and saltmarsh), and non-BC habitats inter-tidal mudflats and beaches and subtidal coral reefs, coastal sediments and offshore sediments. The boundary between coastal and offshore habitats and waters set at 100-m depth (following Pinet 2003). Studies that sampled a mix of habitats but did not specify the habitat at each sampling location were omitted from the review. We did not include other habitats (e.g., macroalgal beds, rocky reefs) because they either only cover a small area globally, or because they do not contain sedimentary areas that are routinely sampled for microplastics (MPs).

### Literature retrieval

This study conducted a systematic review of the literature which finished on 28th July 2023, following Pullin and Stewart (2006) and Field and Gillett (2010). The Web of Science (All Databases) was selected for literature searches because it supports truncation/wildcards, is capable of interpreting Boolean operators and is able to provide the maximum number of relevant articles from the searches (Gusenbauer and Haddaway 2020).

The search string used in the Web of Science included the three elements of our research question, namely, the exposure (e.g., microplastic*, micro-plastic*, and “micro plastic*”), the target habitats (e.g., beach*, mudflat*, sediment*, mangrove*, seagrass*, saltmarsh*, ocean*, and sea*) and the environment where the MP is found (e.g., sediment*, sand*, mud*, water*) linked by the Boolean operator “AND”. Within each element the search terms were linked by the operator “OR” and the wildcard character “*” was included to increase capture (see SM Table 2 for search string). We checked the search function with 5 previously identified relevant papers (Supplementary Information (SI) Table 1), and all were captured using the constructed search string on Web of Science.

**Table 1:** Number of data sets retrieved for each habitat (sediments and water) in each continent. The “Total” heading indicates the number of data sets for habitat or continent with each data set. The Sample No. is the total number of samples that were analysed in all data sets combined

Continent	Beach	Mangrove	Mudflats	Coral Reef	Saltmarsh	Seagrass	Coastal sediments	Offshore sediments	Coastal water	Offshore water	Total	Sample No
Africa	8	2	0	0	0	0	6	3	5	1	25	1398
Antarctica	0	0	0	0	0	0	0	4	2	0	6	51
Asia	65	30	13	13	4	6	71	8	41	1	252	7689
Europe	45	0	2	0	2	6	34	14	10	3	116	4460
North America	14	0	0	2	3	1	8	2	8	1	39	2041
Oceania	2	0	0	1	0	0	5	0	2	0	10	281
South America	18	3	1	1	1	0	7	1	10	0	42	2260
Classified by Ocean*	0	0	0	0	0	0	0	0	0	34	34	1258
Total	152	35	16	17	10	13	131	32	78	40	524	19,438
Sample No	7561	1427	1055	380	191	266	4102	310	2324	1822	19,438	

*Cruises that sampled oceans across two or more continents

### Article screening

The criteria for inclusion in the review were studies that provided an estimate of MP abundance with associated error and the number of samples for a defined habitat. After screening of the title, abstract and finally text, 334 papers of the 4875 papers identified on Web of Science were selected for the current meta-analysis. To be included in the meta-analysis studies had to have extractable data on MP abundance, an estimate of variance, sample number, habitat at the sample location, and geographical location. Non-critical data such as the size range of particles analysed were extracted alongside information such as MP detection technique.

### Data analysis

Each study generated one data set, or more if the study included several habitats or continents, each with an estimate of MP abundance (mean and standard deviation) resulting from a number of samples. MP density data were standardised to the most common units used. For sediments this was the number of particles in the top 5 cm of sediment m^−2^ following the protocol of Harris (2020). MP density presented as items per unit weight of dry sediment was converted to a volume estimate by multiplying by the dry bulk density of the sediment (see SI Table 2 for mean values used if no estimate provided) and applied to the first 5 cm of the sediment in 1 m squared. Similarly, seawater MP densities were commonly measured using a variety of surface towed nets where the amount of MP captured divided by the area covered by the net provided estimates of MP per unit area, that were standardised to MP per m^2^. Those MP densities given per volume of filtered seawater were rescaled to MP in 300 L of seawater, which equates to MP m^−2^ in the top 30 cm of the water column which is similar to the average net depth. All values are presented as means and 95% confidence intervals (CI) unless otherwise stated. We defined three major climatic regions as, tropical (extending from 23.5°N to 23.5°S), temperate (extending from 23.5° to 66.5° in both the northern and southern hemispheres) and polar (extending from 66.5° north and south towards the pole).

**Table 2:** Estimated upper and lower 95% confidence intervals of the weight and number of microplastic (MP) particles in each of the marine habitats

Ecosystem	Data sets	Area (km^2^)	MP abundance (counts m^−2^)	Global MP particles (total counts × 10^12^MP)***	Total weight (million tonnes)
Mangrove	35	135882^a^	32,454–121,791	4410–16,549	1.2–4.7
Seagrass	13	2202^b^	3706–25,863	8–57	0.002–0.02
Coral reef	17	284300^f^	9135–25,855	2597–7351	0.736–2.08
Saltmarsh	10	3510^b^	5338–11,717	19–41	0.01–0.01
Mudflats^#^	16	127900^c^	6499–49,743	831–6362	0.2–1.8
Beaches*	152	17205^d^	14,635–87,910	252–1512	0.07–0.43
Coastal sediments (< 100 m depth)	132	32242540^e^	14,462–90,883	466,292–2,930,299	137–836
Offshore sediments (> 100 m depth)	32	329640970^e^	14,702–36,863	4,846,284–12,151,439	1368–3437
Coastal water	78	32242540^e^	871–21,308	28,086–687,023	8–195
Offshore water	40	329640970^e^	289–2649	95,328–873,346	27–247
	524			5,444,106–16,673,980	1543–4724

^#^Assumed to be synonymous with tidal flat area. *Assumed a mean beach width of 50 m multiplied by the beach length. **Using average MP particle weight of 0.283 mg particle^−1^ for particles with a size of 0.063–5 mm (Klein et al. 2015). ***MP abundance × Area. ^a^Spalding and Leal (2021). ^b^Himes-Cornell et al. (2018). ^c^Murray et al. (2022). ^d^Luijendijk et al. (2018). ^e^Harris et al. (2014)

We used a random effects meta-analysis to examine differences in the mean MP densities found in the targeted coastal and offshore habitats using the *metamean* function in *meta* package (Balduzzi et al. 2019) in R Version 4.2.2 (R Core Team 2022), this function weighs studies using the inverse variance method for pooling the data so that larger studies are given more weight than smaller ones (Schwarzer 2023). As “metamean” ignores zero values thereby possibly inflating the resulting overall means, for the two studies where no MP were recorded in a habitat, a mean value of 0.0001 ± 0.0001 MP m^−2^ was used. We think this is a reasonable balance between the sampling effects where small sample volumes (a liter of seawater or a few grams of dry sediment) may not capture low MP densities and the bias caused by the exclusion of studies where no MP were found.

Although global differences in MP density may exist between different Blue Carbon habitats, these might be driven by the different geographical distributions of different habitats (for example saltmarsh replaces mangrove at temperate latitudes, and temperate latitudes on average have more effective waste management). We therefore also evaluated if MP density was higher in Blue Carbon (BC) than in adjacent non-BC habitats in studies that compared this, through a meta-analysis, using the log(BC MP density/nonBC MP density) as our response variable (lnRR). We only included studies that directly compared MP densities in BC habitats with bordering non-BC habitats. We used a weighted linear mixed-effects model (*rma.uni* function in R package *metafor*, (Viechtbauer 2010)) with restricted maximum-likelihood (REML) estimator for three different analyses.

Different MP studies use different sieve sizes and detection methods, we examine how the detection limit of minimum MP particle size influenced the reported MP densities. An initial examination of the patterns in MP densities suggested that densities were higher in studies that reported smaller MP sizes. We examine the relationship of the minimum reported MP particle size on MP density using a linear regression and used this regression to predict the MP densities for each study where minimum MP particle size was reported. To conform with the assumptions of linear regression, the MP abundance data was log transformed. Subsequently, this relationship was used to standardize the observed MP density per study to a minimum particle size of 0.01 mm. It was not possible to correct the MP weight estimates for particle size because particle weight distributions are not routinely reported. We also conducted sensitivity analysis on the impact of site selection, and visualization method of MP. Studies were classified as urban, rural, or mixed as described by the papers authors or by proximity to urban areas (< or > 20 km) and MP identification method whether or not the polymer types were identified using techniques such as pyrolysis-GC–MS, FTIR, RAMAN, or other spectroscopic technique compared to studies where MP were identified visually through microscopic techniques. We omitted studies that only sampled in urban areas and those using only visual techniques and compared the resulting MP densities with the mean MP values for all studies to examine the impact of biased site selection and misidentification of MP might have on the MP densities.

We assessed the potential impact of the environmental MP concentrations by reanalysing the data of Mason et al (2022). The 72 studies in the meta-analysis of Mason et al (2022) examined the impact of a range of MP concentrations in seawater (g l^−1^) and sediments (g kg^−1^) on the biological processes of benthic fauna under controlled conditions in the laboratory. To obtain current mean environmental MP concentrations in grams we reran “metamean” for the studies that gave MP densities in seawater per unit volume (standardised to MP l^−1^ to get mean MP l^−1^ values of 13.0 ± 12.0 and 2.4 ± 1.8) for coastal and offshore water, respectively, and used the mean marine sediment MP abundance of 1005 ± 398 MP kg^−1^ (Fig. 2b). These mean values were multiplied by an average MP particle weight of 0.28 mg, derived from the mean weight of 9042 MP particles with a size range of 0.063 to 5 mm extracted from a series of river sediment samples (Klein et al. 2015) to give MP densities expressed as g l^−1^ and kg^−1^. This resulted in MP concentrations of 0.67 and 3.68 mg of MP l^−1^ in offshore and coastal waters, respectively, and 0.28 g of MP kg^−1^ in sediments. We estimated the biological effects for subsets of studies from Mason et al. (2022) selecting studies where organisms were exposed to concentrations of MPs that were equivalent to, or less than,the mean sediment and water MP concentrations from our meta-analysis. From the Mason et al. (2022) data the effect size of the experimental treatment relative to the control was calculated using Hedge’s *g* effect size (Borenstein et al. 2009) and interpreted using thresholds suggested by Cohen (2013): 0.2 indicated a small effect, ~ 0.5 indicated a moderate effect, and > 0.8 indicated a strong effect. A negative g represents a negative impact on the organism. For each biological process, a linear regression was used to look for a relationship between MP exposure concentration and Hedge’s *g* effect size. We also calculated the biological effects of the mean MP concentration for offshore and coastal water and marine sediments estimated by our meta-analysis. For each analysis, a pooled effect size and effect size per biological process was calculated using a random effects model with the “rma.mv” function in the “metafor” package (Viechtbauer 2010) in R Version 4.2.2 (R Core Team 2022), including study ID as a random effect. Effect sizes were visualized using forest plots, where confidence intervals which did not overlap with zero this represents a significant impact.

## Results

**Fig. 1:**
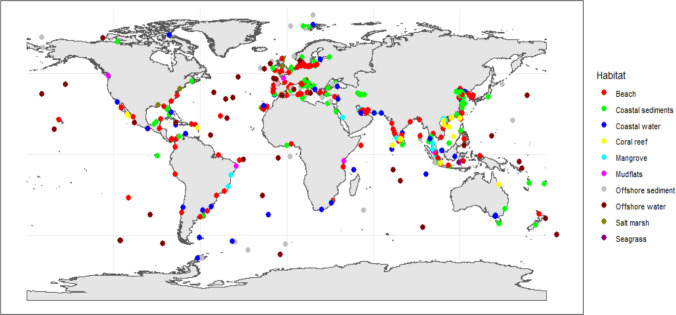
Location of the 524 studies used in the global analysis on microplastic densities in the ten marine habitats

A very large body of studies reporting microplastic (MP) in marine habitats exists, and 334 studies containing 524 datasets passed our selection criteria. The research effort differed widely between habitats with 30% of the 528 datasets used concentrating on beaches and 25% on subtidal coastal sediments. 11% of the datasets focused on Blue Carbon habitats with mangrove attracting the most attention (7%). Research was geographically uneven (Fig. 1) with most effort concentrated in Asia (47%) and Europe (22%) and least in Africa (5%), Oceania (2%), and Antarctica (1%) (Table 1). China and India produced 18% and 6% of the datasets

### Geographic and habitat variation in microplastic density

**Fig. 2:**
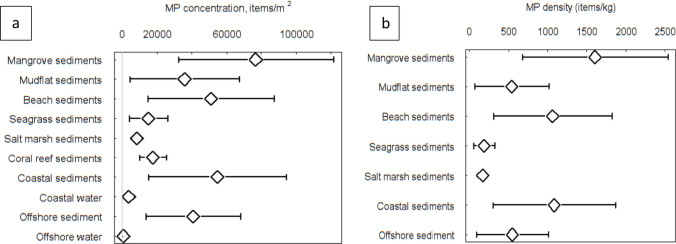
Mean (and 95% confidence levels) microplastic (MP) density in each habitat, expressed as **a** (items m^−2^) and **b** (items kg^−1^). See SI Tables 3 and 4 for values and sample numbers

**Fig. 3:**
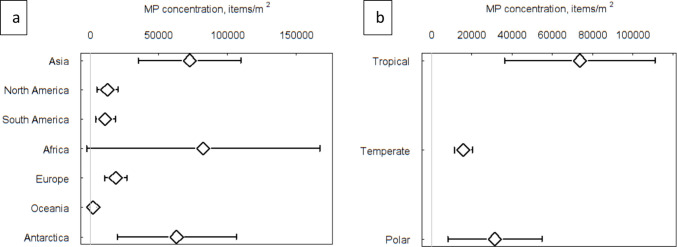
Mean (and 95% confidence levels) microplastic (MP) density in sediments (items m^−2^) per continent (**a**) and climatic region (**b**). See SI Tables 5 and 6 for values and sample number

Microplastics have a global, widespread distribution and were present in all sediment and water habitats studied with the exception of two studies that found no MP in four coastal sediment samples off Southern Portugal (Lechthaler et al. 2020) and three offshore sediment samples in the Congo Canyon (Van Cauwenberghe et al. 2013). The mean (± 95%CI) MP density in all habitats studied was 37,921 (± 13,925) items m^−2^, with significant differences between habitats (*Q* = 81.3, df = 9, *p* < 0.0001). Mean MP sediment density was 47,250 (± 18,298) items m^−2^ was significantly greater (*Q* = 24.18, *p* = 0.0001) than mean seawater MP density 7681 (± 5907) items m^−2^. Mangrove sediments had the highest mean density of MP (77,123 items m^−2^) but were only significantly greater than the other Blue Carbon sediments in seagrass, saltmarsh and coral reef (Fig. 2a and SI Table 3). Mean MP abundance in sediments was highest in Asia (71,827 MP m^−2^), which was significantly greater than North and South America, Europe, and Oceania (indicated by confidence intervals that did not overlap). Tropical regions were found to have higher sediment MP densities (72,780 MP m^−2^) than temperate regions (14,322 MP m^−2^) (Fig. 3b and SI Table 6). Of the 228 data sets where MP sediment densities were expressed as MP particles kg^−1^, the relative distributions of abundance over the habitats were very similar to those estimated per unit area (MP m^2^) (Fig. 2b). Mean MP (± 95%CI) abundance in sediments was 1005 ± 398 (MP kg^−1^), there were significant differences between habitats (*Q* = 25.6, df = 6, *p* = 0.0006), and mangrove sediment had the greatest mean densities (1605 ± 931 MP kg^−1^) and had significantly greater MP density than the other Blue Carbon habitats as indicated by confidence intervals (Fig. 2b and SI Tables 4). During screening, we omitted a mixed habitat study, as the habitat at the sampling point was not recorded either as the seagrass and muddy sediments. This study of the Great Bay estuary, Gulf of Maine reported mean densities of 6.9 million MP m^−2^ (Cheng et al. 2021) and was 173 times higher than the mean for all other studies together. Had this study been included, it would have increased mean coastal sediment MP densities by 2.2 times, and if applied to seagrass it would have increased our estimates of MP in seagrass habitats by an order of magnitude.

### Sensitivity analysis of the experimental factors that cause bias to overall mean MP densities

Only 150 of the 332 papers reported the minimum detected size of MP. Minimum particle size ranged from 1.5 to 0.001 mm, with an average of 0.3 mm. Offshore water samples were omitted as low MP abundance in this habitat regardless of MP size caused significant deviation from normality. The minimum size of MP particles detected had a strong and significant effect on the reported abundance of MP m^−2^ (*R*^2^ adj. = 14.2, *F* = 34.48, *p* < 0.001).

1 $$Log_{10}\;(MPm^{-2}+1)=3.92-1.72\times minimum\;MP\;particle\;size(mm)$$

**Fig. 4:**
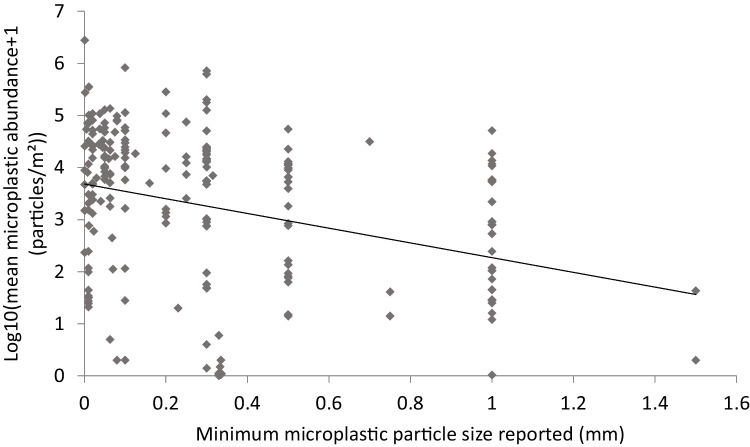
Effect of minimum particle particle size on the density of microplastic particles counted

MP size explains a relatively large amount of variation (14%), given that the large amount of remaining variation relating to the differences in habitats and geography is expected (Fig. 4).

**Fig. 5:**
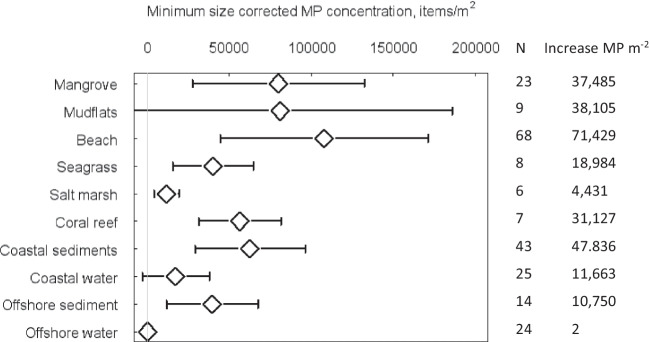
Comparison of the microplastic (MP) abundance and 95% confidence intervals corrected to a minimum particle size of 0.01 mm using the regression slope from Fig. 4 in each of the habitats (*N* = the number of studies that reported a minimum detectable MP particle size, Increase MP m^−2^ represents the increase in the amount of MP resulting from the corrected MP abundance relative to the mean MP abundance for those studies that reported minimum MP particle size)

When we rescaled MP abundance in each study for a minimum particle size to 0.01 mm to reduce the bias of studies that had large minimum detectable particle size, this more than doubled estimated of mean global MP density to 57,953 ± 17,441 particles m^−2^ (SI Table 7) from an uncorrected density of 22,697 ± 8318 particles m^−2^ for those studies that reported the minimum particle size. It was not possible to rescale all samples as not all studies reported minimum particle size, and we therefore report both the corrected and uncorrected estimates in this paper. The effect on different habitats varied (*Q* = 86.3, df = 9, *p* < 0.0001), with smallest increase in MP densities in saltmarsh that had the smallest mean minimum particles size of 0.13 mm. The largest increase in MP abundance resulting from the correction is especially noticeable in beaches where sampling methods such as hand picking (Edo et al. 2019) and sieving of sediments (Koongolla et al. 2018) results in only larger MP particles with a mean size of 0.46 mm being detected (Fig. 5).

**Fig. 6:**
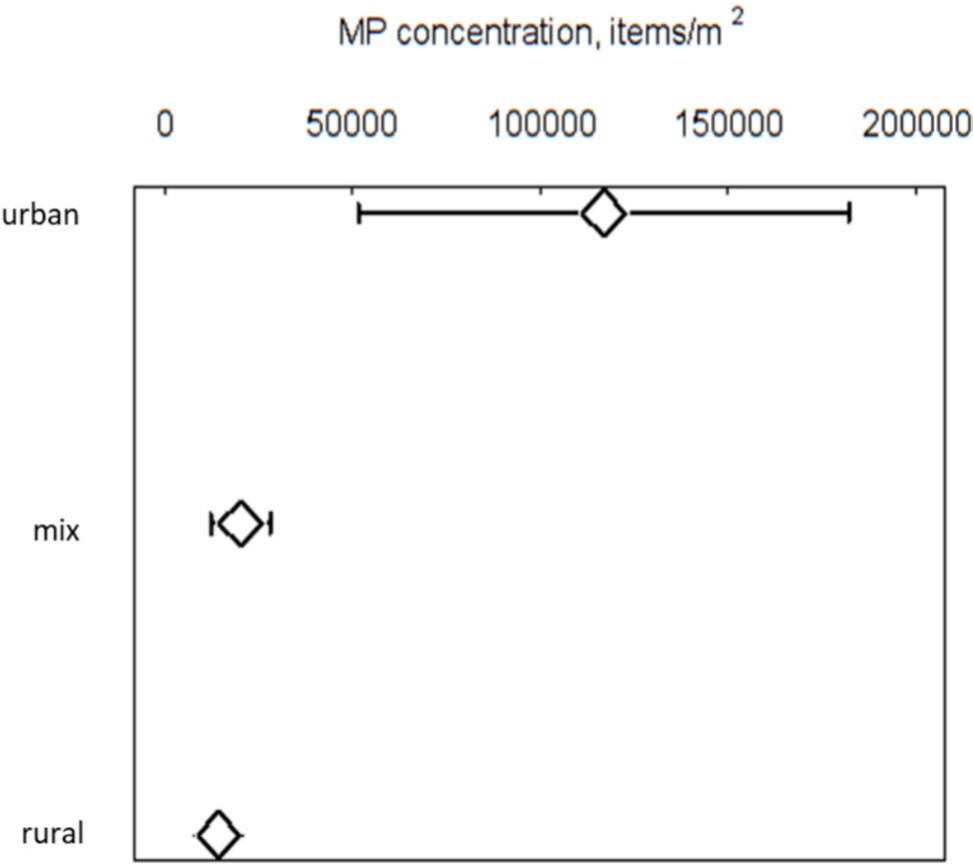
Mean microplastic (MP) concentrations and 95% confidence intervals in studies where the sample sites were situated in urban, rural and a mix of urban and rural environments

Site selection was found to have significant impact on the resulting MP densities, studies that sampled only in urban locations resulted in reported MP densities that were significantly greater than those reported from studies sampled in either a mix of urban and rural sites or in rural sites only (Fig. 6). Sensitivity analysis omitting the urban studies resulted in mean MP (± 95%CI) that were significantly less (17,447 ± 5149 MP m^−2^) compared with when included (37,921 ± 13,925 MP m^−2^). Tropical coastal sediments and beaches were particularly affected by the proximity to urban centers (SI Figs. 2 and 4), although removing these habitats did not significantly alter the overall mean MP densities or those in these habitats. Reported MP densities of (36,465 ± 16,813 MP m^−2^) confirmed through analytical techniques (Raman, FTIR, etc.) were not significantly greater (Metamean *Q* = 0.07, *p* = 0.79) that those reported by visual techniques (39,864 ± 18,277 MP m^−2^) and omission of studies using visually detected MP did not significantly alter the reported MP densities.

### Do Blue Carbon habitats have higher MP densities than adjacent non-Blue Carbon habitats?

**Fig. 7:**
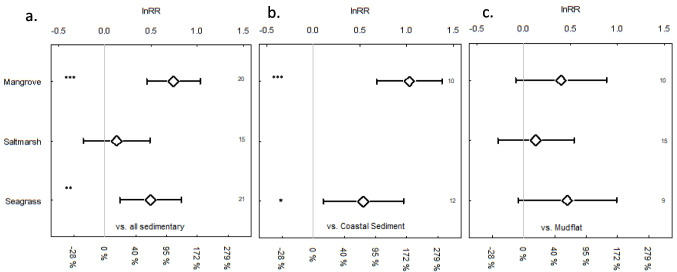
Comparison of the microplastic abundance and 95% confidence intervals (lnRR- ln relative ratios of microplastic abundance (items m^−2^)) in Blue Carbon habitats (mangrove forests, seagrass beds and saltmarsh areas) with adjacent **a** sedimentary habitats, or **b** subtidal coast sediments or **c** intertidal mudflats. Numbers at the left of the plots represent the number of compared areas and significance levels are indicated by stars where *p < 0.05, **p < 0.01, and ***p < 0.001. Confidence levels that overlap with lnRR values of 0 are not significantly different

In the 18 studies that directly compared MP densities between Blue Carbon habitats and adjacent sedimentary habitats (mudflat and coastal sediment), meta-analysis found significantly more MP m^−2^ in seagrass (*z* = 2.93, *p* = 0.003) and mangroves (*z* = 5.0487, *p* < 0.0001) than adjacent sedimentary habitats (coastal sediment and mud flats combined, Fig. 7a). This appeared to be driven by differences between the BC habitats and subtidal coastal sediment (Fig. 7b), as no significant differences were reported between any of the BC habitats and adjacent intertidal mudflats (Fig. 7c).

### Global MP stocks by habitat type

We estimated the global stocks of MP by habitat type by multiplying mean MP densities with the global area of each habitat (Table 2). This analysis suggests that between 5 × 10^18^ and 16 × 10^18^ MP particles weighing between 1.5 and 4.7 billion tonnes of MPs are currently present in marine environment, with surface seawater only responsible for 35–442 million tonnes. Blue Carbon habitats hold less than 0.28% of the MPs found in the sediments of all coastal habitats (excluding offshore sediments). Coastal and offshore sediment hold 99.9% of all sedimentary MP due to the much larger global areas of these habitats.

### Effects of environmental concentrations of MP on the fauna

**Fig. 8:**
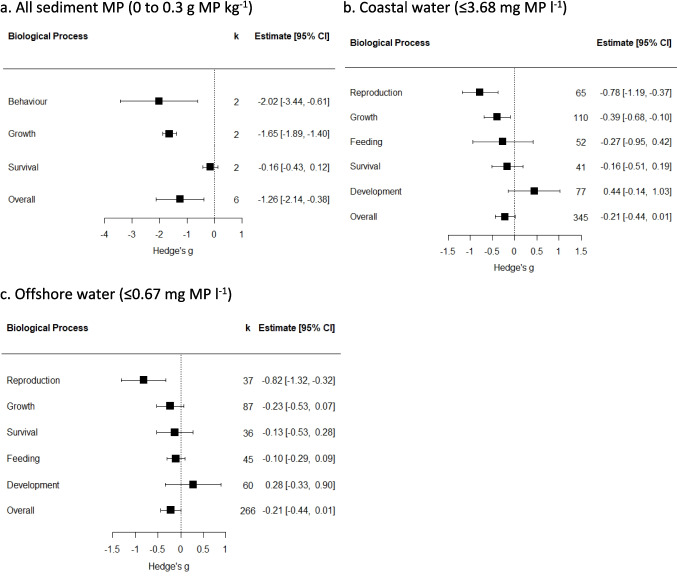
The effects of environmental concentrations of microplastic (MP) in marine sediment (**a**), coastal water (**b**), and offshore water (**c**) on biological processes of marine benthic fauna as demonstrated in laboratory experiments. Effects on each of the processes and overall, as indicated from random-effects modelling. Boxes and error bars represent pooled Hedge’s *g* values and 95% confidence intervals, respectively. K represents the number of case studies, or independent experiments within the same study. Overlap of confidence intervals with 0 indicates non-significance

Our analysis estimated that globally the mean MP concentration was 0.28 g MP kg^−1^ in coastal sediment, 3.68 mg MP l^−1^ in coastal water and 0–0.67 mg MP l^−1^ in offshore water. In our re-analysis of data from Mason et al. (2022), these mean environmental concentrations were found to have significant biological effects on fauna in laboratory exposure experiments (Fig. 8). In sediment this concentration had significantly negative effects on biological processes (Hedge’s *g* value = −1.26 [−1.26 to −0.38], *p* < 0.001) (Fig. 8a). The mean MP concentrations in found in coastal water resulted in mostly negative effects with significant impacts on reproduction and growth in laboratory trials (Fig. 8b). While the lower MP concentrations found in offshore water resulted in only significantly negative impacts on reproduction (Fig. 8c).

**Fig. 9:**
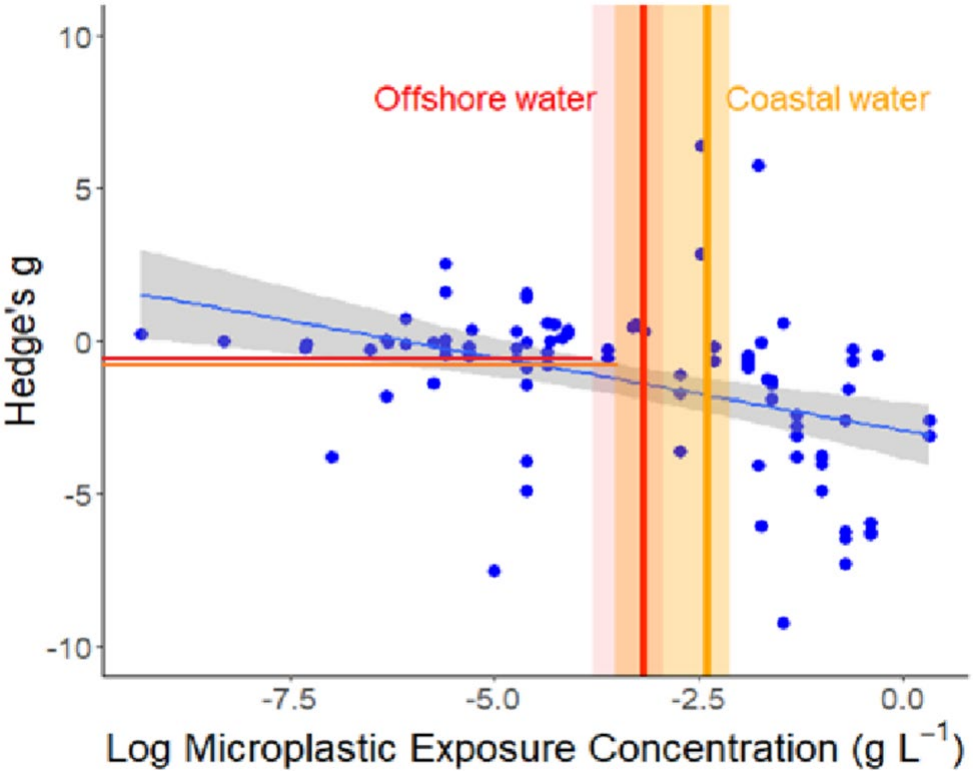
The effects of microplastic exposure concentration on the feeding of marine benthic fauna. Hedge’s *g* effect size with (log) exposure concentration of microplastic particles in sediment or the water column. Grey area shows 95% confidence interval. The orange and red vertical lines and the shading around them indicate the mean and 95% confidence intervals of the MP concentration in coastal and offshore waters estimated from the meta-analysis. The orange and red horizontal line show the intersection point of the 95% confidence interval of the MP concentrations in the coastal and offshore water with the 95% upper confidence level of the regression line

Re-analysis of data from Mason et al. (2022) suggested that increasing MP exposure only resulted in a significant negative impact on feeding (Fig. 9) but not on the other six biological processes (SI Fig. 1). Feeding was significantly negatively impacted by increasing concentrations of MP (Adj. *R*^2^ = 0.17, *f* = 19.13, *p* < 0.001) as indicated by the increasing negative Hedge’s *g* values with increasing Log MP exposure. The negative Hedge’s *g* values at the intersection point of the confidence interval of coastal and offshore water MP concentrations with the upper confidence interval of the regression line indicate that feeding is already being significantly negatively impacted by the current mean environmental concentrations of MP.

## Discussion

We found an average marine microplastic (MP) density of 37,921 ± 13,925 MP m^−2^ (mean ± 95%CI), with sediments (47,250 MP m^−2^) more impacted that seawater (7681 MP m^−2^). These numbers confirm that marine habitats are highly contaminated with MP particles, especially in near-shore environments with higher densities found in coastal water than offshore regions. In contrast Chen et al. (2023) reported modelled MP densities to be higher in the open ocean than along coasts. Our meta-analysis provides the first comparison of MP densities across marine environments and highlights the vulnerability of three Blue Carbon habitats (mangroves, seagrasses and saltmarshes), with mangrove forests especially efficient at trapping MPs. Across the globe the highest mean densities of MP were found in Asia, Africa and Antarctica. The large variation in MP abundance and limited number of studies from both Africa and Antarctica indicate that current estimates of MP concentrations might be subject to bias. To improve the reliability of the estimated global distribution of MP, increased sampling efforts in these underrepresented areas are essential. Furthermore, research from other geographical regions should not be overlooked as current estimates are likely biased by non-random site selection compromising the representativeness of global pollution assessments.

MP densities are highest in tropical regions with Asian MP densities being significantly higher than Europe, Oceania, and North and South America, and are much lower in the water column than in sediments. Riverine outputs of MP agree with these global patterns with Asia responsible for 86% of the global riverine plastic input and 17 out of the 20 worst polluting rivers occurring within the tropics (Lebreton et al. 2017) and 17 of the 20 worst plastic polluting countries also in the tropics (Meijer et al. 2021). These patterns might be driven by socio-economic factors like rapid industrialization and population growth and the fact that a large proportion of the plastic waste from Europe and North America is shipped to poorer countries where waste management may be less rigorous (Brooks et al. 2018; Zhao et al. 2022).

Among sedimentary habitats, mangroves stand out with particularly high MP densities (Wedinger 2021) showing the highest MP concentration and density of all habitats assessed. The complex root structures and reduced hydrodynamics in vegetated coastal habitats such as mangroves and seagrasses further enhance sedimentation and retention of plastic debris, creating localized hotspots of MP deposition (Cordova et al. 2023). This pattern is likely reinforced by the slow degradation rates typical of low-energy, anaerobic environments like mangrove sediments, where plastics are shielded from UV radiation and mechanical abrasion (Chamas et al. 2020), which permit long-term accumulation. Interestingly, the other Blue Carbon habitats: saltmarshes and seagrasses did not show elevated MP densities compared with other sedimentary habitats in this global comparison. However, when studies compared adjacent Blue Carbon and non-Blue Carbon habitats, mangroves and seagrass were found to accumulate significantly more MPs than the adjacent coastal sediments, but not mudflats. The impact of vegetation on slowing water movement and hence increasing sedimentation correlates to the deposition of MP, but not in saltmarshes. Saltmarshes which replace mangroves in temperate zones like Europe and North America, are exposed to significantly less plastic pollution than tropical mangroves. This disparity is underscored by the regional differences in plastic litter contributions from rivers, with Asian waterways, especially in the tropics identified as major sources of marine plastic litter (Mai et al. 2023). Further research is needed to explore how the proximity to MP sources, the magnitude of MP release, and habitat retention factors such as hydrodynamics, and vegetation density and type, affect MP distribution in the marine environment.

The large variance around our mean estimates of MP densities reflects the great differences in the amount of plastic waste that is deposited in the adjacent coastal strip (Meijer et al. 2021). Urbanization, economic development, industrial and tourism activities, sewage discharge, river proximity have all been cited as driving increases in MPs density in coastal and offshore habitats (Browne et al. 2011; Frere et al. 2017; Meijer et al. 2021; Tata et al. 2020). We found that studies that only sampled urban locations that are sources of MPs reported significantly greater mean MP densities than those that sampled in mixed and rural locations, with urban beaches, mangroves and coastal sediments showing particularly elevated MP densities (SI Fig. 4). The number of study sites was approximately balanced between urban and rural, with the majority of studies including both types. However omitting studies that only samples in sites classified as urban, halved mean global microplastic density, suggesting that site selection has a significant impact on our analysis.

Our estimates of global plastic stocks by habitat seem high, relative to the total amount of plastic waste that is expected to be present in the oceans. There is an estimated 6,200 million tonnes of plastic waste in landfills and the natural environment (extrapolated from Geyer et al. 2017), while our meta-analysis suggests the marine environment could be holding between 1543 and 4724 million tonnes of MP. These are very high estimates compared to the global plastic waste production, and our upper estimate seems unlikely to be correct. Our seawater MP estimates of 35 to 442 million tonnes are also much higher than previous assessments of world’s oceans that appear to increasing over time, 7–35 thousand tonnes (Cózar et al. 2014), 93–236 thousand tonnes (Van Sebille et al. 2015), and more recently 1.1–4.9 million tonnes (Eriksen et al. 2023). However our estimates of MP density are conservative (coastal water-12,995 MP m^−3^, offshore water – 2365 MP m^−3^) compared to a recent study that reported global mean MP densities in seawater of 20,463 MP^−3^ (Tang et al. 2023). Moreover, our estimates of the mass of MP may be more representative as they include MP concentrations in 100’s of seawater samples filtered through a 0.45-µm mesh taken around the globe during the Adventure Scientists’ Global Microplastics Initiative (https://www.adventurescientists.org/microplastics.html) and described in Lima et al. (2021) and our estimate weighs each studies by study size. These previous studies used plankton nets with mesh sizes of 200 µm (Cózar et al. 2014), 330 µm (Van Sebille et al. 2015) and 320 µm (Eriksen et al. 2023 (95% of the included studies)) which have been shown to underestimate MP abundance (McEachern et al. 2019). If we accept that between 1.2% and 4% of the plastic waste generated enters the ocean within a year (Meijer et al. 2021) indicating that at least 7.6 to 25.2 million tonnes of plastic are currently in the ocean, then the question is how much has degraded to MP? Plastic degradation times vary (Chamas et al. 2020), but much of the plastic waste is fast degrading packaging that is discarded within a year (Geyer 2020). However, even though a large proportion of the discarded plastics will have degraded to become MP it is not likely that all these have reached the sea, which shows that our upper estimate is undoubtedly overestimating global MP stocks in marine habitats. The vast majority (78 to 89%) of global marine MPs are estimated to be held in offshore sediments based on relatively few studies (only 6% of the studies) indicating that this could be a major source of bias. The global overestimation suggests that MP studies have been targeting particularly polluted locations that are not necessarily representative of the MP loads in the wider environment. Many studies tend to target habitats affected by the extremes of plastic pollution from heavily polluted Tokyo Bay sediments with > 5 million MP m^−2^ in (Wang et al. 2021) to the pristine Faafu Atoll, Maldives with 22 MP m^−2^ (Saliu et al. 2018). This has important implications for using sampled MP densities when assessing the possible impacts of MP on the health of the habitat, because it is likely to result in an overestimate of such impacts. Our sensitivity analysis indicated that sampling in only urban environments can influence subsequent global estimates for coastal habitats such as beaches and coastal sediments, but does not impact offshore estimates and as such does not significantly reduce the overall global estimate (SI Table 8).

Despite the current evidence of marine habitats acting as plastic sinks, our global estimates must be regarded with caution as the sampling methods and units used varied greatly between studies. We showed how both sampling and subsequent detection methods have a very large impact on the resulting estimates of MP abundance, in particular when this results in the omission of the smaller MP down to 0.01 mm. For example, most estimates of MP densities in water are based on towed plankton, bongo or manta nets (mesh size 330 µm) (e.g., Eriksen et al. 2014). Comparison of net-based samples with discrete samples, taken in water bottles or autosamplers that are vacuum filtered (pore size 1.2 µm), resulted in a MP density that were 230 times greater for discrete samples than plankton net estimates (McEachern et al. 2019). Minimum particle size also varied with MP detection method, visual detection using a microscope can only differentiate MP from other materials when particles are greater than 500 µm (Lv et al. 2021), staining with Nile Red and counterstaining with DAPI (Stanton et al. 2019) in conjunction with a fluorescing microscope can distinguish MP particles > 1 µm (Hu et al. 2023). While analytical techniques such as Fourier transform infrared (FTIR) spectroscopy can detect plastic type in particles > 10 µm, Raman spectroscopy can detect particles down 1 µm, but the cost of these analytical methods are prohibitive for most researchers (Hu et al. 2023). Our sensitivity analysis of bias caused by the identification method found that our estimated mean MP abundance was similar regardless of whether MP were identified visually with a microscope or by methods that analysed polymer composition such as FTIR and Ramen. However, we found that the ability to detect smaller MP particles, results in an increase in the total MP counted in agreement with Cózar et al. (2014) and Hale et al. (2020) who suggested that due to fragmentation of plastic litter, the number of particles in the marine environment increases exponentially with decreasing particle size. For the 150 studies that reported a minimum particle size, we used the relationship of MP abundance to minimum particle size to adjust the MP abundance as if all studies had been able to detect particles as small as 10 µm; we found that the mean MP abundance in some habitats increase significantly. As microplastic (MP) particle size decreases, individual particle weight declines exponentially. Consequently, improved detection of smaller MPs will dramatically inflate global particle counts and perceived abundance, while contributing relatively little to the total mass of marine MPs. This disparity means that estimates based on particle number may overstate ecological exposure, whereas weight-based assessments may underrepresent the ubiquity and potential biological impact of smaller MPs (Thacharodi et al. 2024). To facilitate comparison between studies, we therefore urge researchers to report the minimum particle size and MP abundance both in terms of area (MP particles m^−2^) and mass (MP particles kg^−1^ or m^−3^).

In this study we found mean concentrations (± 95%CI) of MP per kilo of dry sediments varied from 1605 ± 931 MP kg^−1^ in mangrove forests to 169 ± 74 MP kg^−1^ in saltmarshes, which is equivalent to 0.45g kg^−1^ and 0.05 g kg^−1^, respectively. In coastal waters the concentration is much less at 13.4 ± 12.4 MP l^−1^ or 0.004g l^−1^ (SI Table 4). While concentrations in the sediments seem high, a critical question remains; Have MP levels reached a threshold that impact resident fauna?

Recent reviews of the effects of MP when exposed to marine organisms in the water under experimental conditions have found conclusive evidence to suggest that MP impact all aspects of biological performance in these organisms resulting in decreased growth, survival and reproductive output (Berlino et al. 2021; Salerno et al. 2021). However, none mention at what MP concentration these negative impacts occur, and teasing out these data appears complicated by toxicity that changes with MP particle size (Berlino et al. 2023), MP shape, chemical composition, faunal life stage, feeding strategy and phylum (Mason et al. 2022). Both, Berlino et al. (2023) and Mason et al. (2022) showed that increasing MP concentration did not consistently amplify negative effects. Nevertheless, individual studies have shown that lugworm oxygen consumption increased and worm cast production decreased with increasing MP concentrations (0 to 20 g of PVC MP kg^−1^) alongside reduced microalgal productivity (Green et al. 2016). Our meta-analysis indicates that mean MP densities in marine sediments, coastal waters and offshore waters have reached levels at which laboratory experiments have demonstrated significant biological effects. This suggests that current marine microplastic loads, especially those urban-influenced habitats, are sufficient to disrupt key biological processes in fauna such as growth, feeding, survival and reproduction. Our re-analysis of Mason et al (2022) further supports this, revealing a decline in feeding activity associated with increasing MP concentrations. The variability in MP toxicity likely reflects the susceptibility of the different organisms as well as differences in MP toxicity. However, we acknowledge that the controlled conditions of laboratory experiments may not reflect the variability and complexity of real-world exposure scenarios (Grattagliano et al. 2025; Palmer and Herat 2021). Factors such as particle aging, biofilm formation, and environmental dilution can alter MP toxicity in situ (Thacharodi et al. 2024). Moreover, species-specific responses and trophic interactions in natural habitats may buffer or amplify observed effects (Palmer and Herat 2021).

Blue Carbon habitats, in particular mangroves where the highest MP densities are found, are biodiversity hotspots and crucial nursery grounds for important commercial species (Deegan et al. 2000; Laegdsgaard and Johnson 1995). Evidence suggests that the MP accumulated in these habitats are ingested by resident fauna, with multiple studies detecting MPs across a range of mangrove species (Deng et al. 2021). As MP concentration in most coastal habitats, and in particular mangroves, have reached levels at which biological processes like growth and reproduction are being impacted, it is likely that productivity is being compromised. This could have serious consequences for fisheries yields and the health of local populations that are exposed to this contaminated seafood (Huang et al. 2020).

The persistence of MP in marine habitats of several hundred years (Ajith et al. 2020) mean that even with a global moratorium on plastic use, high MP densities will remain in the marine environment for decades. While our global averages may be influenced by non-random sampling, they reflect widespread contamination across diverse habitats. The biological impacts of these concentrations are likely to cascade through ecosystems reducing productivity and increasing health risks associated with seafood consumption. The projected increases in MP concentrations are particularly concerning given the observed correlation between MP load and biological harm.

These findings emphasize the urgent need for standardized global protocols in MP sampling and detection. Without harmonized methodologies and representative site selection, global MP estimates risk misinforming environmental risk assessments and policy decisions. Policymakers must prioritize investment in inclusive, geographically balanced monitoring programs, particularly in data-poor regions such as Africa and Antarctica. Integrating MP metrics into coastal management plans, waste governance frameworks, and international agreements will be essential to mitigate plastic pollution and safeguard vulnerable marine ecosystems. Blue Carbon habitats, especially mangroves and seagrasses, should be recognized not only for their ecological value but also for their susceptibility to MP retention, and thus incorporated into targeted conservation and pollution mitigation strategies. To prevent further ecological degradation, urgent improvements in plastic waste management are essential. Current MP concentrations in marine habitats, particularly in urban and tropical regions, are already sufficient to impair biological function. Without intervention, rising MP loads will further compromise coastal productivity and food security.

## Supplementary Information

Below is the link to the electronic supplementary material.

ESM1 (DOCX 1.05 MB)

## Data Availability

Data will be made available on request.

## References

[CR1] Ajith N, Arumugam S, Parthasarathy S, Manupoori S, Janakiraman S (2020) Global distribution of microplastics and its impact on marine environment—a review. Environ Sci Pollut Res Int 27(21):25970–25986. 10.1007/s11356-020-09015-5 32382901 10.1007/s11356-020-09015-5

[CR2] Andrady AL (2017) The plastic in microplastics: a review. Mar Pollut Bull 119(1):12–22 28449819 10.1016/j.marpolbul.2017.01.082

[CR3] Balduzzi S, Rücker G, Schwarzer G (2019) How to perform a meta-analysis with R: a practical tutorial. Evid Based Ment Health 22(4):153–160 31563865 10.1136/ebmental-2019-300117 PMC10231495

[CR4] Bergmann M, Gutow L, Klages M (2015) Marine anthropogenic litter. Springer Nature. p. 447. 10.1007/978-3-319-16510-3

[CR5] Berlino M, Mangano MC, De Vittor C, Sarà G (2021) Effects of microplastics on the functional traits of aquatic benthic organisms: a global-scale meta-analysis. Environ Pollut 285:117174. 10.1016/j.envpol.2021.117174 33957511 10.1016/j.envpol.2021.117174

[CR6] Berlino M, Sarà G, Mangano M (2023) Functional trait-based evidence of microplastic effects on aquatic species. Biology 12(6):811 37372096 10.3390/biology12060811 PMC10294819

[CR7] Borenstein M, Hedges L, Higgins J, Rothstein H (2009) Introduction to meta-analysis. Wiley, Chichester (UK)

[CR8] Brooks AL, Wang S, Jambeck JR (2018) The Chinese import ban and its impact on global plastic waste trade. Sci Adv 4(6):eaat0131. 10.1126/sciadv.aat0131 29938223 10.1126/sciadv.aat0131 PMC6010324

[CR9] Browne MA, Crump P, Niven SJ, Teuten E, Tonkin A, Galloway T, Thompson R (2011) Accumulation of microplastic on shorelines woldwide: sources and sinks. Environ Sci Technol 45(21):9175–9179 21894925 10.1021/es201811s

[CR10] Cappa P, Walton ME, Paler MKO, Taboada EB, Hiddink JG, Skov MW (2023) Impact of mangrove forest structure and landscape on macroplastics capture. Mar Pollut Bull 194:115434 37634347 10.1016/j.marpolbul.2023.115434

[CR11] Carmen S (2021) Microbial capability for the degradation of chemical additives present in petroleum-based plastic products: a review on current status and perspectives. J Hazard Mater 402:123534 33254737 10.1016/j.jhazmat.2020.123534

[CR12] Chamas A, Moon H, Zheng J, Qiu Y, Tabassum T, Jang JH, Abu-Omar M, Scott SL, Suh S (2020) Degradation rates of plastics in the environment. ACS Sustain Chem Eng 8(9):3494–3511

[CR13] Chandra S, Lahiri D, Nag M, Bhattacharya D, Pandit C, Pandit S, Sharma K, Rajeev M, Gill HS, Rustagi S (2025) A review on microbial-biofilm mediated mechanisms in marine microplastics degradation. Antonie Van Leeuwenhoek 118(10):152. 10.1007/s10482-025-02163-z 40952525 10.1007/s10482-025-02163-z

[CR14] Chen B, Zhang Z, Wang T, Hu H, Qin G, Lu T, Hong W, Hu J, Penuelas J, Qian H (2023) Global distribution of marine microplastics and potential for biodegradation. J Hazard Mater 451:131198. 10.1016/j.jhazmat.2023.131198 36921415 10.1016/j.jhazmat.2023.131198

[CR15] Cheng ML, Lippmann TC, Dijkstra JA, Bradt G, Cook S, Choi J-G, Brown BL (2021) A baseline for microplastic particle occurrence and distribution in Great Bay Estuary. Mar Pollut Bull 170:112653 34198152 10.1016/j.marpolbul.2021.112653

[CR16] Cohen J (2013) Statistical power analysis for the behavioral sciences. Routledge, New York. p. 567

[CR17] Cordova MR, Ulumuddin YI, Lubis AA, Kaisupy MT, Wibowo SPA, Subandi R, Yogaswara D, Purbonegoro T, Renyaan J, Nurdiansah D (2023) Microplastics leaving a trace in mangrove sediments ever since they were first manufactured: a study from Indonesia mangroves. Mar Pollut Bull 195:115517 37690405 10.1016/j.marpolbul.2023.115517

[CR18] Cózar A, Echevarría F, González-Gordillo JI, Irigoien X, Úbeda B, Hernández-León S, Palma ÁT, Navarro S, García-de-Lomas J, Ruiz A (2014) Plastic debris in the open ocean. Proc Natl Acad Sci U S A 111(28):10239–10244 24982135 10.1073/pnas.1314705111 PMC4104848

[CR19] Cozzolino L, Nicastro KR, Zardi GI, de Los Santos CB (2020) Species-specific plastic accumulation in the sediment and canopy of coastal vegetated habitats. Sci Total Environ 723:138018 32213414 10.1016/j.scitotenv.2020.138018

[CR20] Deegan LA, Hughes JE, Rountree RA (2000) Salt marsh ecosystem support of marine transient species. Concepts and controversies in tidal marsh ecology. (ed. by M.P. Weinstein and D.A. Kreeger), pp. 333-365. Kluwer Academic Publishers, Dordrecht, The Netherlands.

[CR21] Deng H, He J, Feng D, Zhao Y, Sun W, Yu H, Ge C (2021) Microplastics pollution in mangrove ecosystems: a critical review of current knowledge and future directions. Sci Total Environ 753:142041. 10.1016/j.scitotenv.2020.142041 32906050 10.1016/j.scitotenv.2020.142041

[CR22] Edo C, Tamayo-Belda M, Martínez-Campos S, Martín-Betancor K, González-Pleiter M, Pulido-Reyes G, García-Ruiz C, Zapata F, Leganés F, Fernández-Piñas F (2019) Occurrence and identification of microplastics along a beach in the biosphere reserve of Lanzarote. Mar Pollut Bull 143:220–227 31789157 10.1016/j.marpolbul.2019.04.061

[CR23] Eriksen M, Lebreton LC, Carson HS, Thiel M, Moore CJ, Borerro JC, Galgani F, Ryan PG, Reisser J (2014) Plastic pollution in the world’s oceans: more than 5 trillion plastic pieces weighing over 250,000 tons afloat at sea. PLoS ONE 9(12):e111913 25494041 10.1371/journal.pone.0111913 PMC4262196

[CR24] Eriksen M, Cowger W, Erdle LM, Coffin S, Villarrubia-Gómez P, Moore CJ, Carpenter EJ, Day RH, Thiel M, Wilcox C (2023) A growing plastic smog, now estimated to be over 170 trillion plastic particles afloat in the world’s oceans—urgent solutions required. PLoS ONE 18(3):e0281596. 10.1371/journal.pone.0281596 36888681 10.1371/journal.pone.0281596 PMC9994742

[CR25] Field AP, Gillett R (2010) How to do a meta-analysis. Br J Math Stat Psychol 63(3):665–694 20497626 10.1348/000711010X502733

[CR26] Frere L, Paul-Pont I, Rinnert E, Petton S, Jaffré J, Bihannic I, Soudant P, Lambert C, Huvet A (2017) Influence of environmental and anthropogenic factors on the composition, concentration and spatial distribution of microplastics: a case study of the Bay of Brest (Brittany, France). Environ Pollut 225:211–222 28371735 10.1016/j.envpol.2017.03.023

[CR27] Frère L, Maignien L, Chalopin M, Huvet A, Rinnert E, Morrison H, Kerninon S, Cassone A-L, Lambert C, Reveillaud J (2018) Microplastic bacterial communities in the Bay of Brest: influence of polymer type and size. Environ Pollut 242:614–625 30014939 10.1016/j.envpol.2018.07.023

[CR28] Galgani F, Brien AS-O, Weis J, Ioakeimidis C, Schuyler Q, Makarenko I, Griffiths H, Bondareff J, Vethaak D, Deidun A (2021) Are litter, plastic and microplastic quantities increasing in the ocean? Microplastics and Nanoplastics 1(1):1–4

[CR29] Gaylarde CC, de Almeida MP, Neves CV, Neto JAB, da Fonseca EM (2023) The importance of biofilms on microplastic particles in their sinking behavior and the transfer of invasive organisms between ecosystems. In: Micro, vol 3. MDPI, pp 320–337. 10.3390/micro3010022

[CR30] Geyer R (2020) Production, use, and fate of synthetic polymers Plastic waste and recycling. Elsevier, pp 13–32

[CR31] Geyer R, Jambeck JR, Law KL (2017) Production, use, and fate of all plastics ever made. Sci Adv 3(7):e1700782 28776036 10.1126/sciadv.1700782 PMC5517107

[CR32] Grattagliano A, Grattagliano Z, Manfra L, Libralato G, Biandolino F, Prato E (2025) An overview on microplastics hazards to the marine ecosystem and humans’ health. Water 17(7):916

[CR33] Green DS, Boots B, Sigwart J, Jiang S, Rocha C (2016) Effects of conventional and biodegradable microplastics on a marine ecosystem engineer (*Arenicola marina*) and sediment nutrient cycling. Environ Pollut 208:426–434 26552519 10.1016/j.envpol.2015.10.010

[CR34] Gusenbauer M, Haddaway NR (2020) Which academic search systems are suitable for systematic reviews or meta-analyses? Evaluating retrieval qualities of Google Scholar, PubMed, and 26 other resources. Res Synth Methods 11(2):181–217 31614060 10.1002/jrsm.1378 PMC7079055

[CR35] Hale RC, Seeley ME, La Guardia MJ, Mai L, Zeng EY (2020) A global perspective on microplastics. J Geophys Res Oceans 125(1):e2018JC014719

[CR36] Harris PT (2020) The fate of microplastic in marine sedimentary environments: a review and synthesis. Mar Pollut Bull 158:111398. 10.1016/j.marpolbul.2020.111398 32753183 10.1016/j.marpolbul.2020.111398

[CR37] Harris PT, Macmillan-Lawler M, Rupp J, Baker EK (2014) Geomorphology of the oceans. Mar Geol 352:4–24. 10.1016/j.margeo.2014.01.011

[CR38] Harrison JP, Hoellein TJ, Sapp M, Tagg AS, Ju-Nam Y, Ojeda JJ (2018) Microplastic-associated biofilms: a comparison of freshwater and marine environments. In: Wagner M, Lambert S (eds) Freshwater microplastics : emerging environmental contaminants? Springer International Publishing, Cham, pp 181–201

[CR39] Haward M (2018) Plastic pollution of the world’s seas and oceans as a contemporary challenge in ocean governance. Nat Commun 9(1):667 29445166 10.1038/s41467-018-03104-3 PMC5812987

[CR40] Hidalgo-Ruz V, Gutow L, Thompson RC, Thiel M (2012) Microplastics in the marine environment: a review of the methods used for identification and quantification. Environ Sci Technol 46(6):3060–3075 22321064 10.1021/es2031505

[CR41] Himes-Cornell A, Pendleton L, Atiyah P (2018) Valuing ecosystem services from blue forests: a systematic review of the valuation of salt marshes, sea grass beds and mangrove forests. Ecosystem Serv 30:36–48. 10.1016/j.ecoser.2018.01.006

[CR42] Horton AA, Dixon SJ (2018) Microplastics: an introduction to environmental transport processes. Wires Water 5(2):e1268

[CR43] Hu W, Tang R, Yuan S, Gong M, Shi P, Wang W, Hu Z-H (2023) Modification of fluorescence staining method for small-sized microplastic quantification: focus on the interference exclusion and exposure time optimization. Environ Sci Pollut Res Int 30(19):56330. 10.1007/s11356-023-26226-8 36917381 10.1007/s11356-023-26226-8

[CR44] Huang J-S, Koongolla JB, Li H-X, Lin L, Pan Y-F, Liu S, He W-H, Maharana D, Xu X-R (2020) Microplastic accumulation in fish from Zhanjiang mangrove wetland, South China. Sci Total Environ 708:134839 31785901 10.1016/j.scitotenv.2019.134839

[CR45] Plastics_Europe (2018) Plastics – the Facts (2018). In: 2023. https://plasticseurope.org/wp-content/uploads/2021/10/2018-Plastics-the-facts.pdf. Accessed 4 Oct 2023

[CR46] Plastics_Europe (2022) Plastics – the Facts 2022. In: 2023. https://plasticseurope.org/knowledge-hub/plastics-the-facts-2022/. Accessed 4 Oct 2023

[CR47] Islam MS, Tanaka M (2004) Impacts of pollution on coastal and marine ecosystems including coastal and marine fisheries and approach for management: a review and synthesis. Mar Pollut Bull 48(7–8):624–649 15041420 10.1016/j.marpolbul.2003.12.004

[CR48] Klein S, Worch E, Knepper TP (2015) Occurrence and spatial distribution of microplastics in river shore sediments of the Rhine-Main area in Germany. Environ Sci Technol 49(10):6070–6076 25901760 10.1021/acs.est.5b00492

[CR49] Koongolla JB, Andrady A, Kumara PTP, Gangabadage C (2018) Evidence of microplastics pollution in coastal beaches and waters in southern Sri Lanka. Mar Pollut Bull 137:277–284 30503436 10.1016/j.marpolbul.2018.10.031

[CR50] Laegdsgaard P, Johnson CR (1995) Mangrove habitats as nurseries: unique assemblages of juvenile fish in subtropical mangroves in eastern Australia. Mar Ecol Prog Ser 126:67–81

[CR51] Lebreton L, Van Der Zwet J, Damsteeg J, Slat B, Andrady A, Reisser J (2017) River plastic emissions to the world’s oceans. Nat Commun 8:15611 28589961 10.1038/ncomms15611 PMC5467230

[CR52] Lechthaler S, Schwarzbauer J, Reicherter K, Stauch G, Schüttrumpf H (2020) Regional study of microplastics in surface waters and deep sea sediments south of the Algarve Coast. Reg Stud Mar Sci 40:101488

[CR53] Lima AR, Ferreira GV, Barrows AP, Christiansen KS, Treinish G, Toshack MC (2021) Global patterns for the spatial distribution of floating microfibers: Arctic Ocean as a potential accumulation zone. J Hazard Mater 403:123796 33264901 10.1016/j.jhazmat.2020.123796

[CR54] Luijendijk A, Hagenaars G, Ranasinghe R, Baart F, Donchyts G, Aarninkhof S (2018) The state of the world’s beaches. Sci Rep 8(1):1–11 29703960 10.1038/s41598-018-24630-6 PMC5923213

[CR55] Lv L, Yan X, Feng L, Jiang S, Lu Z, Xie H, Sun S, Chen J, Li C (2021) Challenge for the detection of microplastics in the environment. Water Environ Res 93(1):5–15 31799785 10.1002/wer.1281

[CR56] Mai L, Sun X, Zeng EY (2023) Country-specific riverine contributions to marine plastic pollution. Sci Total Environ 874:162552. 10.1016/j.scitotenv.2023.162552 36870495 10.1016/j.scitotenv.2023.162552

[CR57] Mason VG, Skov MW, Hiddink JG, Walton M (2022) Microplastics alter multiple biological processes of marine benthic fauna. Sci Total Environ. 10.1016/j.scitotenv.2022.157362 10.1016/j.scitotenv.2022.157362 35843327

[CR58] McEachern K, Alegria H, Kalagher AL, Hansen C, Morrison S, Hastings D (2019) Microplastics in Tampa Bay, Florida: abundance and variability in estuarine waters and sediments. Mar Pollut Bull 148:97–106 31422308 10.1016/j.marpolbul.2019.07.068

[CR59] Meijer LJJ, van Emmerik T, van der Ent R, Schmidt C, Lebreton L (2021) More than 1000 rivers account for 80% of global riverine plastic emissions into the ocean. Sci Adv 7(18):eaaz5803. 10.1126/sciadv.aaz5803 33931460 10.1126/sciadv.aaz5803 PMC8087412

[CR60] Murray NJ, Worthington TA, Bunting P, Duce S, Hagger V, Lovelock CE, Lucas R, Saunders MI, Sheaves M, Spalding M (2022) High-resolution mapping of losses and gains of Earth’s tidal wetlands. Science 376(6594):744–749 35549414 10.1126/science.abm9583

[CR61] Napper IE, Thompson RC (2020) Plastic debris in the marine environment: history and future challenges. Glob Chall 4(6):1900081 32685195 10.1002/gch2.201900081 PMC7268196

[CR62] Paler MKO, Tabañag IDF, Siacor FDC, Geraldino PJL, Walton MEM, Dunn C, Skov MW, Hiddink JG, Taboada EB (2022) Elucidating the surface macroplastic load, types and distribution in mangrove areas around Cebu Island, Philippines and its policy implications. Sci Total Environ 838:156408 35660612 10.1016/j.scitotenv.2022.156408

[CR63] Palmer J, Herat S (2021) Ecotoxicity of microplastic pollutants to marine organisms: a systematic review. Water Air Soil Pollut 232(5):195

[CR64] Pinet PR (2003) Invitation to Oceanography, 3rd edn. Jones and Bartlett Publishers Inc, Massachusetts, USA. p, p 555

[CR65] Pullin AS, Stewart GB (2006) Guidelines for systematic review in conservation and environmental management. Conserv Biol 20(6):1647–1656 17181800 10.1111/j.1523-1739.2006.00485.x

[CR67] R Core Team (2022) R: A language and environment for statistical computing. R foundation for statistical computing, Vienna, Austria. https://www.R-project.org/

[CR68] Reisser J, Shaw J, Wilcox C, Hardesty BD, Proietti M, Thums M, Pattiaratchi C (2013) Marine plastic pollution in waters around Australia: characteristics, concentrations, and pathways. PLoS ONE 8(11):e80466 24312224 10.1371/journal.pone.0080466 PMC3842337

[CR69] Salerno M, Berlino M, Mangano M, Sarà G (2021) Microplastics and the functional traits of fishes: a global meta-analysis. Glob Change Biol. 10.1111/gcb.15570 10.1111/gcb.15570 33638211

[CR70] Saliu F, Montano S, Garavaglia MG, Lasagni M, Seveso D, Galli P (2018) Microplastic and charred microplastic in the Faafu Atoll, Maldives. Mar Pollut Bull 136:464–471 30509830 10.1016/j.marpolbul.2018.09.023

[CR71] Schwarz A, Ligthart T, Boukris E, Van Harmelen T (2019) Sources, transport, and accumulation of different types of plastic litter in aquatic environments: a review study. Mar Pollut Bull 143:92–100 31789171 10.1016/j.marpolbul.2019.04.029

[CR72] Schwarzer G (2023) Metamean: meta-analysis of single means, In: meta: General Package for Meta-Analysis. (https://rdrr.io/cran/meta/man/metamean.html). Accessed 15 Sept 2023

[CR73] Spalding M & Leal M (eds) (2021). The state of the world’s mangroves 2021. Global Mangrove Alliance. p.136 . 10.5479/10088/119867

[CR74] Stanton T, Johnson M, Nathanail P, Gomes RL, Needham T, Burson A (2019) Exploring the efficacy of Nile red in microplastic quantification: a costaining approach. Environ Sci Technol Lett 6(10):606–611

[CR75] Tang L, Feng J-C, Li C, Liang J, Zhang S, Yang Z (2023) Global occurrence, drivers, and environmental risks of microplastics in marine environments. J Environ Manage 329:116961. 10.1016/j.jenvman.2022.116961 36542885 10.1016/j.jenvman.2022.116961

[CR76] Tata T, Belabed BE, Bououdina M, Bellucci S (2020) Occurrence and characterization of surface sediment microplastics and litter from North African coasts of Mediterranean Sea: preliminary research and first evidence. Sci Total Environ 713:136664 32019027 10.1016/j.scitotenv.2020.136664

[CR77] Thacharodi A, Meenatchi R, Hassan S, Hussain N, Bhat MA, Arockiaraj J, Ngo HH, Le QH, Pugazhendhi A (2024) Microplastics in the environment: a critical overview on its fate, toxicity, implications, management, and bioremediation strategies. J Environ Manage 349:119433. 10.1016/j.jenvman.2023.119433 10.1016/j.jenvman.2023.119433 39492398

[CR78] Thushari GGN, Senevirathna JDM (2020) Plastic pollution in the marine environment. Heliyon. 10.1016/j.heliyon.2020.e04709 10.1016/j.heliyon.2020.e04709 PMC7475234 32923712

[CR79] Van Cauwenberghe L, Vanreusel A, Mees J, Janssen CR (2013) Microplastic pollution in deep-sea sediments. Environ Pollut 182:495–499 24035457 10.1016/j.envpol.2013.08.013

[CR80] Van Sebille E, Wilcox C, Lebreton L, Maximenko N, Hardesty BD, Van Franeker JA, Eriksen M, Siegel D, Galgani F, Law KL (2015) A global inventory of small floating plastic debris. Environ Res Lett 10(12):124006

[CR81] Viechtbauer W (2010) Conducting meta-analyses in R with the metafor package. J Stat Softw 36:1–48

[CR82] Villarrubia-Gómez P, Cornell SE, Fabres J (2018) Marine plastic pollution as a planetary boundary threat–the drifting piece in the sustainability puzzle. Mar Policy 96:213–220

[CR83] Walton MEM, Samonte-Tan GPB, Primavera JH, Edwards-Jones G, Le Vay L (2006) Are mangroves worth replanting? The direct economic benefits of a community-based reforestation project. Environ Conserv 33(4):335–343. 10.1017/s0376892906003341

[CR84] Walton MEM, Al-Maslamani I, Chatting M, Smyth D, Castillo A, Skov MW, Le Vay L (2021) Faunal mediated carbon export from mangroves in an arid area. Sci Total Environ. 10.1016/j.scitotenv.2020.142677 10.1016/j.scitotenv.2020.142677 33077211

[CR85] Wang Y, Nakano H, Xu H, Arakawa H (2021) Contamination of seabed sediments in Tokyo Bay by small microplastic particles. Estuar Coast Shelf Sci 261:107552

[CR86] Webb HK, Arnott J, Crawford RJ, Ivanova EP (2012) Plastic degradation and its environmental implications with special reference to poly (ethylene terephthalate). Polymers 5(1):1–18

[CR87] Wedinger M (2021) Global plastic pollution in blue carbon coastal systems – a systematic review. MSc Thesis

[CR88] Worm B, Lotze HK, Jubinville I, Wilcox C, Jambeck J (2017) Plastic as a persistent marine pollutant. Annu Rev Environ Resour 42:1–26

[CR89] Wright SL, Kelly FJ (2017) Plastic and human health: a micro issue? Environ Sci Technol 51(12):6634–6647 28531345 10.1021/acs.est.7b00423

[CR90] Yu S, Lu X, Lu H (2025) Marine microbial biofilms on diverse abiotic surfaces. Front Mar Sci. 10.3389/fmars.2025.1482946

[CR91] Zhao C, Qi X, Wang J, Du F, Shi X (2022) Predicting possible new links to future global plastic waste trade networks. Sustainability 14(8):4692

