# Supplementary material for: Global microplastic pollution at levels harmful to marine life
Source: Environ Sci Pollut Res Int. 2025 Nov 24;32(47):27226–41. doi: 10.1007/s11356-025-37149-x (PMC12675771; doi:10.1007/s11356-025-37149-x)
Supplement: Supplementary file 1 — (DOCX 1.05 MB) [file 11356_2025_37149_MOESM1_ESM.docx]

**Supplementary Information**

SI Table 1: The 5 key peer-reviewed papers utilised to quality check the search string

| Habitat | Reference |
| --- | --- |
| Salt marsh & Seagrass | Cozzolino *et al.,* 2020 |
| Seagrass  Mangrove | Jones *et al.,* 2020  Martin *et al.,* 2020 |
| Salt marsh | Yao *et al.,* 2019 |
| Mangrove & Seagrass | Huang *et al.,* 2021 |

SI Table 2: Number of studies resulting estimates of mean dry bulk density (DBD) for each habitat type. DBD references below

| **Habitat** |  | N | mean DBD (g/cm3) | StDev DBD | min | max |
| --- | --- | --- | --- | --- | --- | --- |
| Mangrove |  | 32 | 0.87 | 0.49 | 0.15 | 1.63 |
| Mangrove (Rhizophora sp) |  | 16 | 0.62 | 0.44 | 0.15 | 1.4 |
| Mangrove (other species) |  | 16 | 1.13 | 0.40 | 0.4 | 1.63 |
| Seagrass |  | 21 | 1.15 | 0.37 | 0.2 | 1.8 |
| Saltmarsh |  | 22 | 0.78 | 0.44 | 0.25 | 1.80 |
| Mudflats |  | 10 | 0.73 | 0.35 | 0.31 | 1.25 |
| Beach |  | 5 | 1.56 | 0.15 | 1.41 | 1.78 |
| Coastal Sediment |  | 15 | 1.12 | 0.49 | 0.1 | 1.7 |
| Offshore Sediment |  | 12 | 1.50 | 0.76 | 0.5 | 2.71 |
|  |  | 117 |  |  |  |  |

SI Table 3: Mean, 95% Confidence Interval, and study number (k) for microplastic (MP) density expressed as the number of MP particles per m^2^.

|  | All Locations | | |  | Urban studies exlcuded | | |
| --- | --- | --- | --- | --- | --- | --- | --- |
| **Habitat** | k | mean | ± 95% CI |  | k | mean | ±95%CI |
| Mangrove sediment | 35 | 77123 | ± 44668 |  | 24 | 65157 | ± 52833 |
| Mudflat sediment | 16 | 28121 | ± 21622 |  | 8 | 7486 | ± 12035 |
| Coastal sediments | 132 | 53267 | ± 38230 |  | 84 | 10974 | ± 9782 |
| Coastal water | 78 | 11090 | ± 10218 |  | 58 | 1923 | ± 9825 |
| Beach sediment | 152 | 51272 | ± 36637 |  | 122 | 21381 | ± 7389 |
| Seagrass sediment | 13 | 14784 | ± 11079 |  | 11 | 13065 | ± 13544 |
| Saltmarsh sediment | 10 | 8528 | ± 3189 |  | 2 | 5399 | ± 2665 |
| Coral sediments | 17 | 17495 | ± 8360 |  | 15 | 17793 | ± 11078 |
| Offshore sediment | 32 | 25730 | ± 11078 |  | 32 | 25730 | ± 901 |
| Offshore water | 40 | 1469 | ± 1180 |  | 40 | 1469 | ± 1180 |

SI Table 4: Mean, 95% Confidence Interval, and study number (k) for microplastic (MP) density expressed as the number of MP particles per kg of dried sediment and converted to MP density (g kg^-1^) using the MP weight conversion factor in Table 2 ([Klein et al. 2015](#_ENREF_46)).

| **Habitat** | k | Mean ± 95%CI (MP kg^-1^) | | Mean±95% CI (g kg^-1^) | |
| --- | --- | --- | --- | --- | --- |
| Mangrove sediment | 30 | 1605 | ± 931 | 0.45 | ± 0.19 |
| Mudflat sediment | 11 | 543 | ± 475 | 0.15 | ± 0.02 |
| Coastal sediment | 117 | 1081 | ± 2069 | 0.05 | ± 0.01 |
| Seagrass sediment | 12 | 187 | ± 137 | 0.30 | ± 0.09 |
| Beach sediment | 97 | 1063 | ± 754 | 0.05 | ± 0.03 |
| Saltmarsh sediment | 7 | 169 | ± 74 | 0.31 | ± 0.08 |
| Offshore sediments | 21 | 548 | ± 482 | 0.16 | ± 0.03 |
| All sediments sediment | 295 | 1005 | ±398 | 0.28 | ± 0.17 |
| **Habitat** | k | Mean ± 95% CI (MP l^-1^) | | Mean±95% CI (g l^-1^) | |
| Coastal water | 66 | 13.4 | ± 12.4 | 3.81 | ± 3.51 |
| Offshore water | 29 | 2.4 | ± 1.8 | 0.67 | ± 0.51 |

SI Table 5: Mean, 95% Confidence Interval, and study number (K) for sediment microplastic (MP) density expressed as the number of MP particles per m^2^ for each continent.

|  | k | mean | 95%-CI | |
| --- | --- | --- | --- | --- |
| Asia | 252 | 62566 | 32490 | 92642 |
| North America | 39 | 10164 | 4324 | 16005 |
| South America | 42 | 8051 | 2951 | 13150 |
| Africa | 25 | 56867 | -13244 | 126978 |
| Europe | 116 | 14091 | 9023 | 19159 |
| Oceania | 10 | 2291 | 507 | 4075 |
| Antarctica | 6 | 45603 | 11128 | 80079 |
| Transboundary | 34 | 1612 | 210 | 3015 |

SI Table 6: Mean, 95% Confidence Interval, and study number (K) for microplastic (MP) density expressed as the number of MP particles per m^2^ for each temperature zone.

|  | K | mean | 95%-CI | |
| --- | --- | --- | --- | --- |
| Tropical | 238 | 64677 | 33422 | 95932 |
| Temperate | 227 | 12255 | 9328 | 15181 |
| Polar | 11 | 27159 | 5697 | 48621 |
| Transboundary | 48 | 2082 | 1000 | 3165 |

SI Table 7: Mean, 95% Confidence Interval corrected to a minimum particle size of 0.01mm using the regression slope from Fig.4, and study number (K) for microplastic (MP) density expressed as the number of MP particles per m^2^.

|  | K | mean | 95%-CI | |
| --- | --- | --- | --- | --- |
| Mangrove | 23 | 79941 | 27547 | 132335 |
| Mudflats | 9 | 80981 | -23905 | 185867 |
| Coastal sediments | 43 | 62625 | 29069 | 96181 |
| Coastal water | 25 | 17257 | -3255 | 37770 |
| Beach | 68 | 107864 | 44575 | 171153 |
| Seagrass | 8 | 40134 | 15450 | 64819 |
| Saltmarsh | 6 | 11665 | 4192 | 19137 |
| Coral reef | 7 | 56436 | 31265 | 81606 |
| Offshore sediment | 14 | 39478 | 11578 | 67377 |
| Offshore water | 25 | 8 | 4 | 13 |

SI Table 8: Mean, 95% Confidence Interval, and study number (K) for microplastic (MP) density expressed as the number of MP particles per m^2^ for each temperature zone and degree of urbanization. Superscripts indicate significant differences at the 95% confidence level and “Transboundary” indicates oceanic sampling that crosses between climatic regions.

|  | Urban | | | | Rural | | | | Mix | | | |
| --- | --- | --- | --- | --- | --- | --- | --- | --- | --- | --- | --- | --- |
|  | K | Mean | ± | 95%CI | K | mean | ± | 95%CI | K | mean | ± | 95%CI |
| Polar |  |  |  |  | 10 | 27518 | ± | 23835^bcd^ | 1 | 25000 | ± | 9800^c^ |
| Temperate | 61 | 14114 | ± | 5666^bc^ | 60 | 14040 | ± | 8975^bc^ | 106 | 9920 | ± | 2527^b^ |
| Tropical | 68 | 151319 | ± | 10369^d^ | 74 | 18096 | ± | 13562^bc^ | 96 | 35299 | ± | 20584^cd^ |
| Transboundary |  |  |  |  | 26 | 26 | ± | 11^a^ | 22 | 6451 |  | 2647^b^ |

SI Table 9: Estimated upper and lower 95% confidence intervals of the weight and number of microplastic (MP) particles in each of the marine habitats if the studies that only sampled urban habitats are excluded.

| Ecosystem | Data sets | Area (km^2^) | MP abundance (m^-2^) | | | Global MP particles (10^12^MP) | | | Total Weight (million tonnes) | | |
| --- | --- | --- | --- | --- | --- | --- | --- | --- | --- | --- | --- |
| Mangrove | 24 | 135882^a^ | 12,324 | - | 117,990 | 1,675 | - | 16,033 | 0.5 | - | 4.5 |
| Seagrass | 11 | 2202^b^ | 1,031 | - | 25,100 | 2 | - | 55 | 0.001 | - | 0.02 |
| Coral reef | 15 | 284300^f^ | 8,010 | - | 27,575 | 2,277 | - | 7,840 | 0.645 |  | 2.22 |
| Saltmarsh | 2 | 3510 ^b^ | -4,426 | - | 15,224 | -16 | - | 53 | -0.004 | - | 0.02 |
| Mudflats^#^ | 8 | 127900^c^ | 97 | - | 14,874 | 12 | - | 1,902 | 0.004 | - | 0.5 |
| Beaches* | 122 | 17205^d^ | 7,837 | - | 34,925 | 135 | - | 601 | 0.04 | - | 0.17 |
| Coastal sediments (<100m depth) | 84 | 32242540^e^ | 8,309 | - | 13,638 | 267,909 | - | 439,740 | 76 | - | 125 |
| Offshore sediments (>100m depth) | 32 | 329640970^e^ | 14,652 | - | 36,809 | 4,829,874 | - | 12,133,610 | 1,368 | - | 3,437 |
| Coastal water | 58 | 32242540^e^ | 1,022 | - | 2,825 | 32,959 | - | 91,072 | 9 | - | 26 |
| Offshore water | 40 | 329640970^e^ | 289 | - | 2,649 | 95,328 | - | 873,346 | 27 | - | 247 |
|  | 395 |  |  |  |  | 5,230,155 | - | 13,564,252 | 1,482 | - | 3,843 |

^#^ assumed to be synonymous with tidal flat area

*assumed a mean beach width of 50m

** using average MP particle weight of 0.283 mg particle^-1^ for particles with a size of 0.063-5 mm ([Klein et al. 2015](#_ENREF_46))

^a^ ([Spalding and Leal 2021](#_ENREF_71)); ^b^ ([Himes-Cornell et al. 2018](#_ENREF_41)); ^c^ ([Murray et al. 2022](#_ENREF_58)); ^d^ ([Luijendijk et al. 2018](#_ENREF_52)); ^e^ ([Harris et al. 2014](#_ENREF_37))


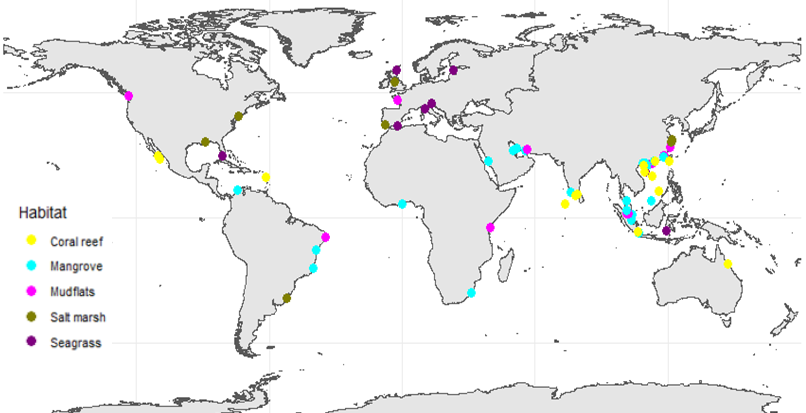


Si Figure 0: Mid points of studies used in in global microplastic estimations for each habitat (omitting locations of beaches, coastal water & sediment, and offshore water & sediment for clarity, all locations are viewable in Figure 1).


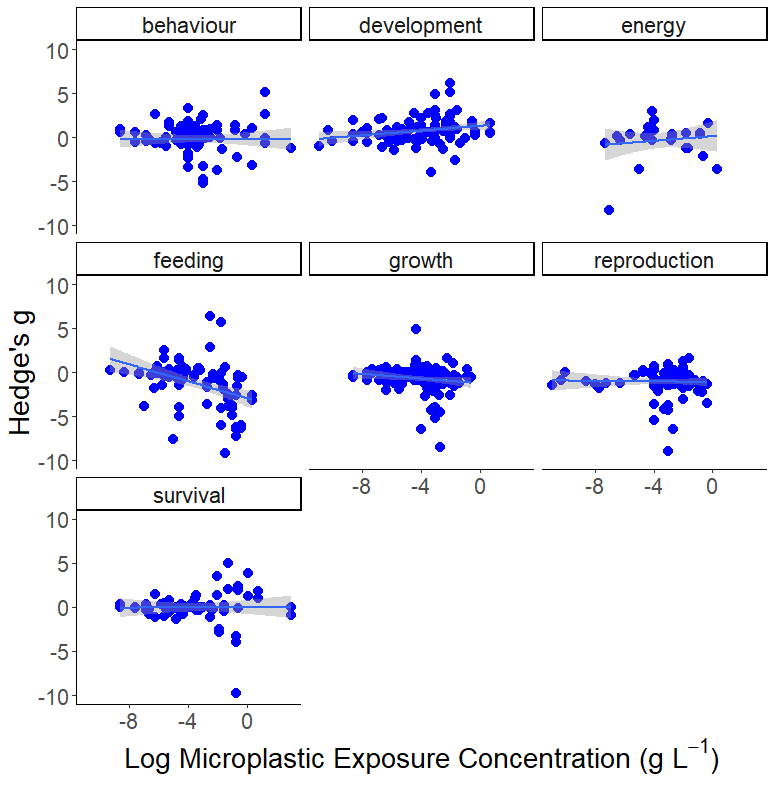


SI Figure 1: The effects of microplastic exposure concentration on 7 biological processes of marine benthic fauna. Hedge’s g effect size with (log) exposure concentration of microplastic particles in sediment or the water column. Grey area shows 95% confidence interval.


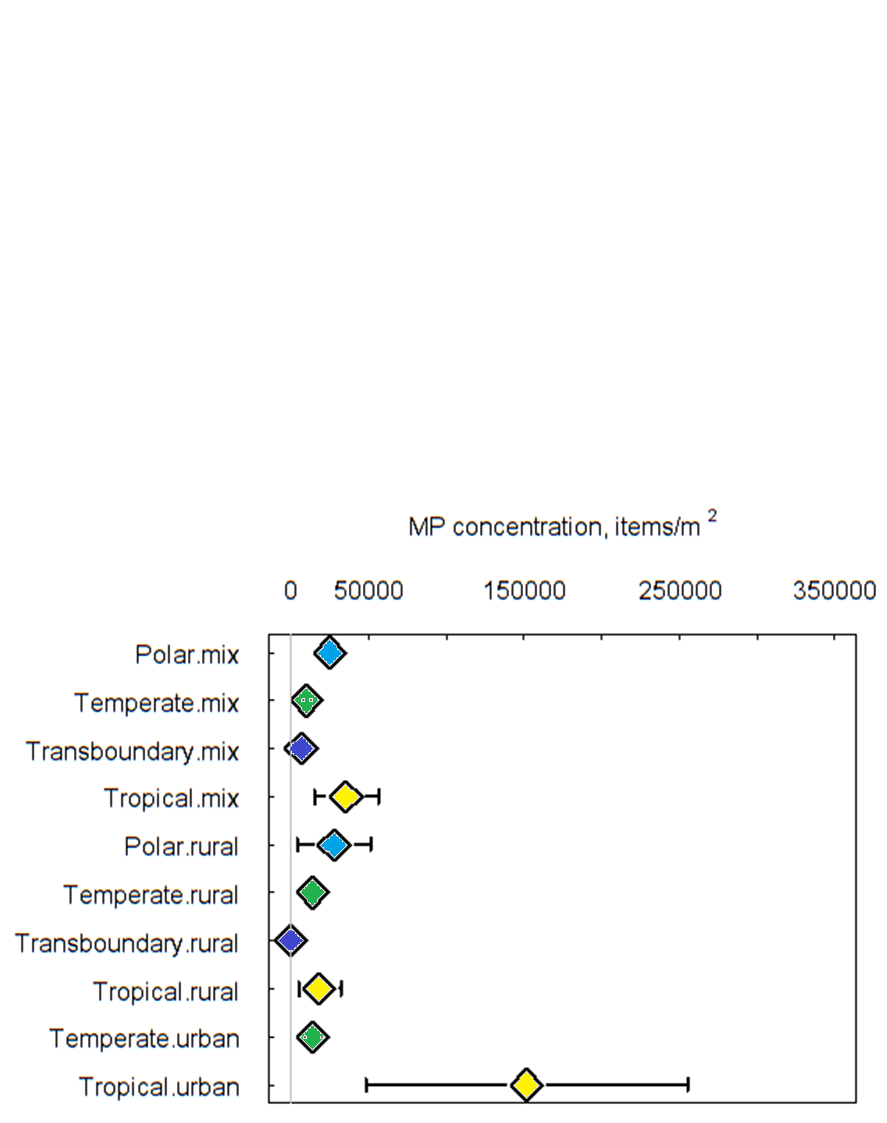


| K |
| --- |
| 1 |
| 106 |
| 22 |
| 96 |
| 10 |
| 60 |
| 26 |
| 74 |
| 61 |
| 68 |

SI Figure 2: Impact of degree on urbanization and climatic region on microplastic densities (MP number m^-2^). Transboundary label indicates ocean sampling that crosses between climatic regions. “K” is the number of studies in each category.


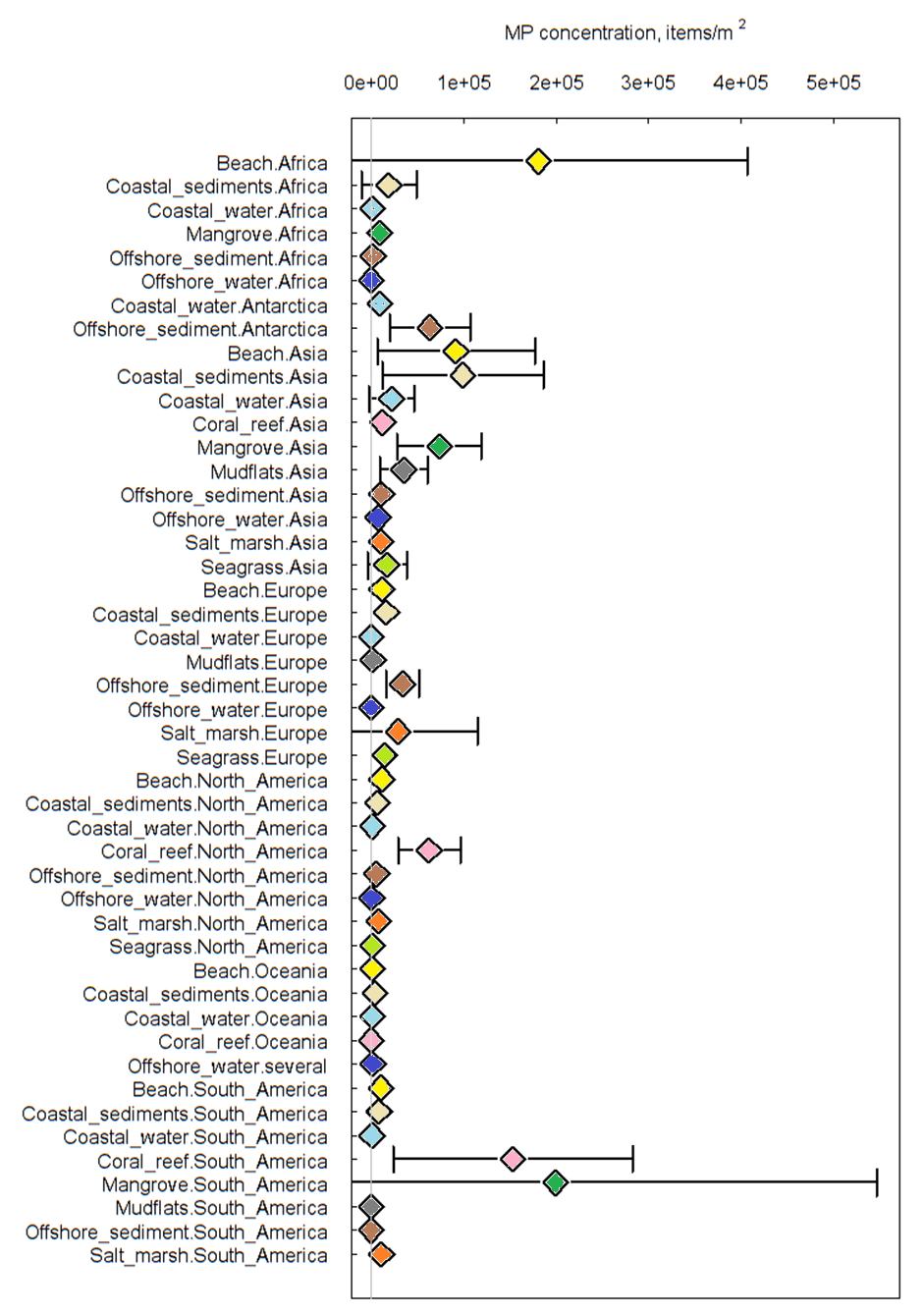


| K |
| --- |
| 8 |
| 6 |
| 5 |
| 2 |
| 3 |
| 1 |
| 2 |
| 4 |
| 65 |
| 71 |
| 41 |
| 13 |
| 30 |
| 13 |
| 8 |
| 1 |
| 4 |
| 6 |
| 45 |
| 35 |
| 10 |
| 2 |
| 14 |
| 3 |
| 2 |
| 6 |
| 14 |
| 8 |
| 8 |
| 2 |
| 2 |
| 1 |
| 3 |
| 1 |
| 2 |
| 5 |
| 2 |
| 1 |
| 34 |
| 18 |
| 7 |
| 10 |
| 1 |
| 3 |
| 1 |
| 1 |
| 1 |
| 524 |

SI Figure 3: Interaction of continent and habitat on the reported mean and 95% confidence intervals of microplastic density. “K” is the number of studies in each category.


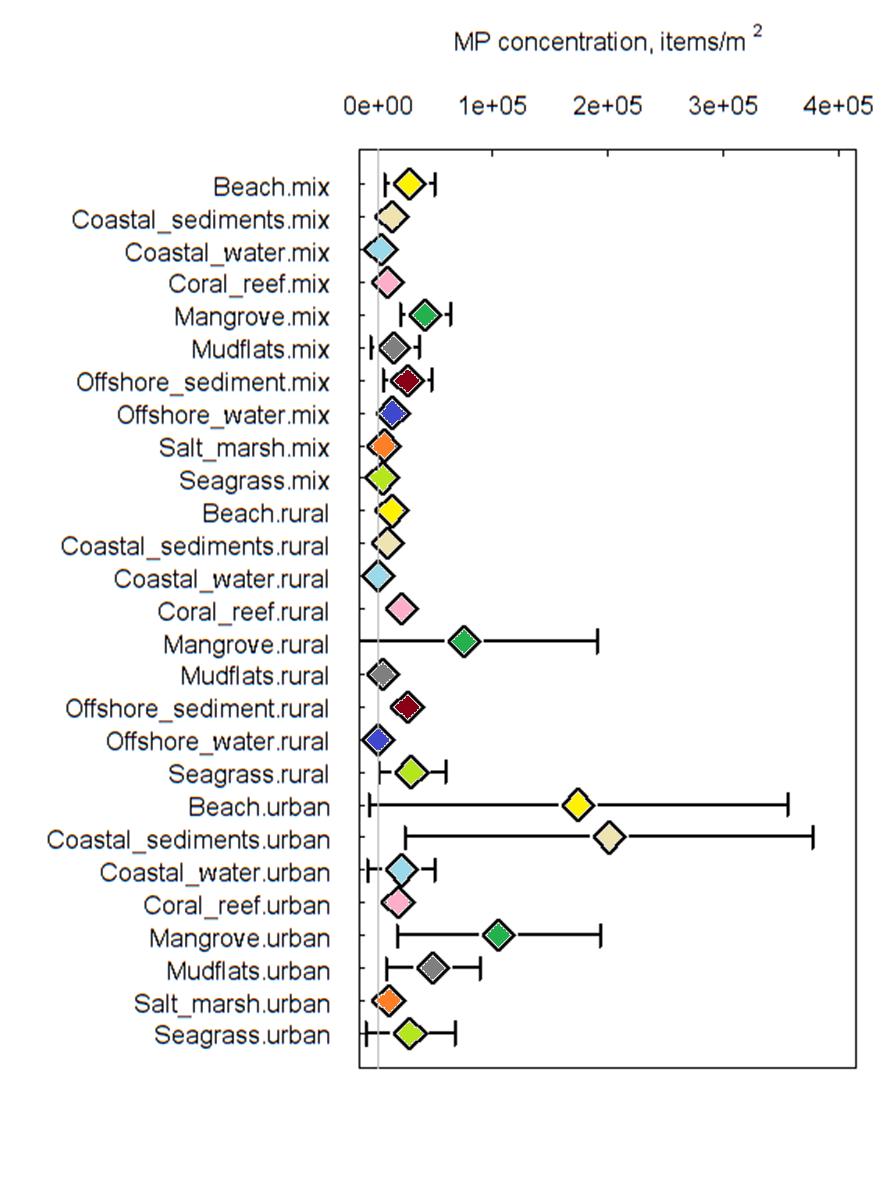


| K |
| --- |
| 74 |
| 59 |
| 44 |
| 5 |
| 15 |
| 4 |
| 7 |
| 8 |
| 2 |
| 7 |
| 48 |
| 25 |
| 14 |
| 10 |
| 9 |
| 4 |
| 24 |
| 32 |
| 4 |
| 30 |
| 48 |
| 20 |
| 2 |
| 11 |
| 8 |
| 8 |
| 2 |

SI Figure 4: Interaction of habitat and degree of urbanisation on the reported mean and 95% confidence intervals of microplastic density. “K” is the number of studies in each category.

**Data references**

| \| Abayomi, O.A., Range, P., Al-Ghouti, M.A., Obbard, J.P., Almeer, S.H. and Ben-Hamadou, R., 2017. Microplastics in coastal environments of the Arabian Gulf. Marine pollution bulletin, 124(1), pp.181-188. \| \| --- \| \| Abidli, S., Antunes, J.C., Ferreira, J.L., Lahbib, Y., Sobral, P. and El Menif, N.T., 2018. Microplastics in sediments from the littoral zone of the north Tunisian coast (Mediterranean Sea). Estuarine, Coastal and Shelf Science, 205, pp.1-9. \| \| Abidli, S., Toumi, H., Lahbib, Y. and El Menif, N.T., 2017. The first evaluation of microplastics in sediments from the complex lagoon-channel of Bizerte (Northern Tunisia). Water, Air, & Soil Pollution, 228(7), pp.1-10. \| \| Adams, J.K., Dean, B.Y., Athey, S.N., Jantunen, L.M., Bernstein, S., Stern, G., Diamond, M.L. and Finkelstein, S.A., 2021. Anthropogenic particles (including microfibers and microplastics) in marine sediments of the Canadian Arctic. Science of The Total Environment, 784, p.147155. \| \| Agharokh, A., S Taleshi, M., Bibak, M., Rasta, M., Torabi Jafroudi, H. and Rubio Armesto, B., 2021. Assessing the relationship between the abundance of microplastics in sediments, surface waters, and fish in the Iran southern shores. Environmental Science and Pollution Research, pp.1-13. \| \| Ahmad, S.W., Yanti, N.A. and Safitri, A.N., 2021, May. Distribution and mitigation efforts for microplastic pollution in Kendari bay as the mainstay coastal tourism area of Southeast Sulawesi. In Journal of Physics: Conference Series (Vol. 1899, No. 1, p. 012012). IOP Publishing. \| \| Ahmed, Q., Ali, Q.M., Bat, L., Öztekin, A., Memon, S. and Baloch, A., 2021. Preliminary Study on Abundance of Microplastic in Sediments and Water Samples Along the Coast of Pakistan (Sindh and Balochistan)-Northern Arabian Sea. Turkish Journal of Fisheries and Aquatic Sciences, 22(1). \| \| Akkajit, P., Tipmanee, D., Cherdsukjai, P., Suteerasak, T. and Thongnonghin, S., 2021. Occurrence and distribution of microplastics in beach sediments along Phuket coastline. Marine Pollution Bulletin, 169, p.112496. \| \| Aliabad, M. K., Nassiri, M., & Kor, K. (2019). Microplastics in the surface seawaters of Chabahar Bay, Gulf of Oman (Makran coasts). Marine pollution bulletin, 143, 125-133. \| \| Alomar, C., Estarellas, F. and Deudero, S., 2016. Microplastics in the Mediterranean Sea: deposition in coastal shallow sediments, spatial variation and preferential grain size. Marine environmental research, 115, pp.1-10. \| \| Álvarez-Hernández, C., Cairós, C., López-Darias, J., Mazzetti, E., Hernández-Sánchez, C., González-Sálamo, J. and Hernández-Borges, J., 2019. Microplastic debris in beaches of Tenerife (Canary Islands, Spain). Marine pollution bulletin, 146, pp.26-32. \| \| Alvarez-Zeferino, J.C., Ojeda-Benítez, S., Cruz-Salas, A.A., Martínez-Salvador, C. and Vázquez-Morillas, A., 2020. Microplastics in Mexican beaches. Resources, Conservation and Recycling, 155, p.104633. \| \| Alves, V.E. and Figueiredo, G.M., 2019. Microplastic in the sediments of a highly eutrophic tropical estuary. Marine pollution bulletin, 146, pp.326-335. \| \| Amin, B., Febriani, I.S., Nurrachmi, I. and Fauzi, M., 2021, March. The Occurrence and Distribution of Microplastic in Sediment of the Coastal Waters of Bengkalis Island Riau Province. In IOP Conference Series: Earth and Environmental Science (Vol. 695, No. 1, p. 012041). IOP Publishing. \| \| Amin, B., Galib, M. and Setiawan, F., 2020. Preliminary Investigation on the Type and Ditribution of Microplastics in the West Coast of Karimun Besar Island. In IOP Conference Series: Earth and Environmental Science (Vol. 430, No. 1, p. 012011). IOP Publishing. \| \| Antunes, J., Frias, J. and Sobral, P., 2018. Microplastics on the Portuguese coast. Marine pollution bulletin, 131, pp.294-302. \| \| Arini, A., Venel, Z., Tabuteau, H., Gigault, J., & Baudrimont, M. 2022). "Early molecular responses of mangrove oysters to nanoplastics using a microfluidic device to mimic environmental exposure." Journal of Hazardous Materials 436. \| \| Arreola-Alarcón, I. M., Reyes-Bonilla, H., Sakthi, J. S., Rodríguez-González, F., & Jonathan, M. P. (2022). Seasonal tendencies of microplastics around coral reefs in selected Marine Protected National Parks of Gulf of California, Mexico. *Marine Pollution Bulletin*, *175*, 113333. \| \| Asadi, M.A., Hertika, A.M.S., Iranawati, F. and Yuwandita, A.Y., 2019. Microplastics in the sediment of intertidal areas of Lamongan, Indonesia. Aquaculture, Aquarium, Conservation & Legislation, 12(4), pp.1065-1073. \| \| Aslam, H., Ali, T., Mortula, M.M. and Attaelmanan, A.G., 2020. Evaluation of microplastics in beach sediments along the coast of Dubai, UAE. Marine pollution bulletin, 150, p.110739. \| \| Azizi, A., Setyowati, W.N., Fairus, S., Puspito, D.A. and Irawan, D.S., 2021, December. Microplastic pollution in the sediment of Jakarta Bay, Indonesia. In IOP Conference Series: Earth and Environmental Science (Vol. 930, No. 1, p. 012010). IOP Publishing. \| \| Bagheri, T., Gholizadeh, M., Abarghouei, S., Zakeri, M., Hedayati, A., Rabaniha, M., Aghaeimoghadam, A. and Hafezieh, M., 2020. Microplastics distribution, abundance and composition in sediment, fishes and benthic organisms of the Gorgan Bay, Caspian sea. Chemosphere, 257, p.127201. \| \| Bakir, A., Desender, M., Wilkinson, T., Van Hoytema, N., Amos, R., Airahui, S., Graham, J. and Maes, T., 2020. Occurrence and abundance of meso and microplastics in sediment, surface waters, and marine biota from the South Pacific region. Marine Pollution Bulletin, 160, p.111572. \| \| Ball, H., 2019. Microplastics in saltmarshes: developing extraction methods and examining past accumulation (Doctoral dissertation, Liverpool John Moores University (United Kingdom)). \| \| Barasarathi, J., Agamuthu, P., Emenike, C. U., & Fauziah, S. H. (2014, August). Microplastic abundance in selected mangrove forest in Malaysia. In *Proceeding of the ASEAN Conference on Science and Technology* (Vol. 5) \| \| Bayo, J., Rojo, D. and Olmos, S., 2019. Abundance, morphology and chemical composition of microplastics in sand and sediments from a protected coastal area: The Mar Menor lagoon (SE Spain). Environmental Pollution, 252, pp.1357-1366. \| \| Baysal, A., Saygin, H. and Ustabasi, G.S., 2020. Microplastic Occurrences in Sediments Collected from Marmara Sea-Istanbul, Turkey. Bulletin of Environmental Contamination and Toxicology, 105(4), pp.522-529. \| \| Beckwith, V.K. and Fuentes, M.M., 2018. Microplastic at nesting grounds used by the northern Gulf of Mexico loggerhead recovery unit. Marine pollution bulletin, 131, pp.32-37. \| \| Bergmann, M., Wirzberger, V., Krumpen, T., Lorenz, C., Primpke, S., Tekman, M.B. and Gerdts, G., 2017. High quantities of microplastic in Arctic deep-sea sediments from the HAUSGARTEN observatory. Environmental science & technology, 51(19), pp.11000-11010. \| \| Bissen, R. and Chawchai, S., 2020. Microplastics on beaches along the eastern Gulf of Thailand–a preliminary study. Marine Pollution Bulletin, 157, p.111345. \| \| Blašković, A., Fastelli, P., Čižmek, H., Guerranti, C. and Renzi, M., 2017. Plastic litter in sediments from the Croatian marine protected area of the natural park of Telaščica bay (Adriatic Sea). Marine pollution bulletin, 114(1), pp.583-586. \| \| Blašković, A., Guerranti, C., Fastelli, P., Anselmi, S. and Renzi, M., 2018. Plastic levels in sediments closed to Cecina river estuary (Tuscany, Italy). Marine pollution bulletin, 135, pp.105-109. \| \| Blumenröder, J., Sechet, P., Kakkonen, J.E. and Hartl, M.G., 2017. Microplastic contamination of intertidal sediments of Scapa Flow, Orkney: a first assessment. Marine Pollution Bulletin, 124(1), pp.112-120. \| \| Bosker, T., Guaita, L. and Behrens, P., 2018. Microplastic pollution on Caribbean beaches in the Lesser Antilles. Marine pollution bulletin, 133, pp.442-447. \| \| Bošković, N., Joksimović, D., Peković, M., Perošević-Bajčeta, A. and Bajt, O., 2021. Microplastics in Surface Sediments along the Montenegrin Coast, Adriatic Sea: Types, Occurrence, and Distribution. Journal of Marine Science and Engineering, 9(8), p.841. \| \| Bridson, J.H., Patel, M., Lewis, A., Gaw, S. and Parker, K., 2020. Microplastic contamination in Auckland (New Zealand) beach sediments. Marine pollution bulletin, 151, p.110867. \| \| Bronzo, L., Lusher, A.L., Schøyen, M. and Morigi, C., 2021. Accumulation and distribution of microplastics in coastal sediments from the inner Oslofjord, Norway. Marine pollution bulletin, 173, p.113076. \| \| Bucol, L.A., Romano, E.F., Cabcaban, S.M., Siplon, L.M.D., Madrid, G.C., Bucol, A.A. and Polidoro, B., 2020. Microplastics in marine sediments and rabbitfish (Siganus fuscescens) from selected coastal areas of Negros Oriental, Philippines. Marine pollution bulletin, 150, p.110685. \| \| Bulleri, F., Ravaglioli, C., Anselmi, S. and Renzi, M., 2021. The sea cucumber Holothuria tubulosa does not reduce the size of microplastics but enhances their resuspension in the water column. Science of The Total Environment, 781, p.146650. \| \| Cannas, S., Fastelli, P., Guerranti, C. and Renzi, M., 2017. Plastic litter in sediments from the coasts of south Tuscany (Tyrrhenian Sea). Marine pollution bulletin, 119(1), pp.372-375. \| \| Carlsson, P., Singdahl-Larsen, C. and Lusher, A.L., 2021. Understanding the occurrence and fate of microplastics in coastal Arctic ecosystems: the case of surface waters, sediments and walrus (Odobenus rosmarus). Science of The Total Environment, p.148308. \| \| Carvalho, J.P., Silva, T.S. and Costa, M.F., 2021. Distribution, characteristics and short-term variability of microplastics in beach sediment of Fernando de Noronha Archipelago, Brazil. Marine Pollution Bulletin, 166, p.112212. \| \| Castro, R.O., da Silva, M.L., Marques, M.R. and de Araújo, F.V., 2020. Spatio-temporal evaluation of macro, meso and microplastics in surface waters, bottom and beach sediments of two embayments in Niterói, RJ, Brazil. Marine Pollution Bulletin, 160, p.111537. \| \| Celis-Hernández, O., Ávila, E., Ward, R.D., Rodríguez-Santiago, M.A. and Aguirre-Téllez, J.A., 2021. Microplastic distribution in urban vs pristine mangroves: Using marine sponges as bioindicators of environmental pollution. Environmental Pollution, 284, p.117391. \| \| Cheang, C. C., Ma, Y., & Fok, L. (2018). Occurrence and composition of microplastics in the seabed sediments of the coral communities in proximity of a metropolitan area. *International journal of environmental research and public health*, *15*(10), 2270. \| \| Chen, C.F., Ju, Y.R., Lim, Y.C., Chen, C.W. and Dong, C.D., 2021. Seasonal variation of diversity, weathering, and inventory of microplastics in coast and harbor sediments. Science of The Total Environment, 781, p.146610. \| \| Chen, M. M., Nie, F. H., Qamar, A., Zhu, D. H., Hu, Y., Zhang, M., Song, Q,L, Lin, H.Y., Chen, ZB.. & Chen, J. J. (2022). "Effects of Microplastics on Microbial Community in Zhanjiang Mangrove Sediments." Bulletin of Environmental contamination and Toxicology 108(5): 867-877. \| \| Chen, M.C. and Chen, T.H., 2020. Spatial and seasonal distribution of microplastics on sandy beaches along the coast of the Hengchun Peninsula, Taiwan. Marine pollution bulletin, 151, p.110861. \| \| Cheng, M.L., Lippmann, T.C., Dijkstra, J.A., Bradt, G., Cook, S., Choi, J.G. and Brown, B.L., 2021. A baseline for microplastic particle occurrence and distribution in Great Bay Estuary. Marine Pollution Bulletin, 170, p.112653. \| \| Chico-Ortiz, N., Mahu, E., Crane, R., Gordon, C. and Marchant, R., 2020. Microplastics in Ghanaian coastal lagoon sediments: Their occurrence and spatial distribution. Regional Studies in Marine Science, 40, p.101509. \| \| Chinfak, N., Sompongchaiyakul, P., Charoenpong, C., Shi, H., Yeemin, T. and Zhang, J., 2021. Abundance, composition, and fate of microplastics in water, sediment, and shellfish in the Tapi-Phumduang River system and Bandon Bay, Thailand. Science of The Total Environment, 781, p.146700. \| \| Chouchene, K., da Costa, J.P., Wali, A., Girão, A.V., Hentati, O., Duarte, A.C., Rocha-Santos, T. and Ksibi, M., 2019. Microplastic pollution in the sediments of Sidi Mansour Harbor in Southeast Tunisia. Marine pollution bulletin, 146, pp.92-99. \| \| Chouchene, K., Prata, J.C., da Costa, J., Duarte, A.C., Rocha-Santos, T. and Ksibi, M., 2021. Microplastics on Barra beach sediments in Aveiro, Portugal. Marine Pollution Bulletin, 167, p.112264. \| \| Chouchene, K., Rocha-Santos, T. and Ksibi, M., 2021. Types, occurrence, and distribution of microplastics and metals contamination in sediments from south west of Kerkennah archipelago, Tunisia. Environmental Science and Pollution Research, 28(34), pp.46477-46487. \| \| Chubarenko, I., Esiukova, E., Khatmullina, L., Lobchuk, O., Grave, A., Kileso, A. and Haseler, M., 2020. From macro to micro, from patchy to uniform: analyzing plastic contamination along and across a sandy tide-less coast. Marine Pollution Bulletin, 156, p.111198. \| \| Cincinelli, A., Scopetani, C., Chelazzi, D., Martellini, T., Pogojeva, M. and Slobodnik, J., 2021. Microplastics in the Black Sea sediments. Science of the Total Environment, 760, p.143898. \| \| Claessens, M., De Meester, S., Van Landuyt, L., De Clerck, K. and Janssen, C.R., 2011. Occurrence and distribution of microplastics in marine sediments along the Belgian coast. Marine pollution bulletin, 62(10), pp.2199-2204. \| \| Clunies-Ross, P.J., Smith, G.P.S., Gordon, K.C. and Gaw, S., 2016. Synthetic shorelines in New Zealand? Quantification and characterisation of microplastic pollution on Canterbury's coastlines. New Zealand Journal of Marine and Freshwater Research, 50(2), pp.317-325. \| \| Constant, M., Kerhervé, P., Mino-Vercellio-Verollet, M., Dumontier, M., Vidal, A.S., Canals, M. and Heussner, S., 2019. Beached microplastics in the northwestern Mediterranean Sea. Marine pollution bulletin, 142, pp.263-273. \| \| Cordova, M.R. and Wahyudi, A., 2016. Microplastic in the deep-sea sediment of Southwestern Sumatran Waters. Marine Research in Indonesia, 41(1), pp.27-35. \| \| Cordova, M.R., Ulumuddin, Y.I., Purbonegoro, T. and Shiomoto, A., 2021. Characterization of microplastics in mangrove sediment of Muara Angke Wildlife Reserve, Indonesia. *Marine Pollution Bulletin*, *163*, p.112012. \| \| Costa, M.F., Silva-Cavalcanti, J.S., Barbosa, C.C., Portugal, J.L. and Barletta, M., 2011. Plastics buried in the inter-tidal plain of a tropical estuarine ecosystem. Journal of Coastal Research, pp.339-343. \| \| Courtene-Jones, W., Maddalene, T., James, M.K., Smith, N.S., Youngblood, K., Jambeck, J.R., Earthrowl, S., Delvalle-Borrero, D., Penn, E. and Thompson, R.C., 2021. Source, sea and sink—A holistic approach to understanding plastic pollution in the Southern Caribbean. Science of The Total Environment, 797, p.149098. \| \| Courtene-Jones, W., Quinn, B., Ewins, C., Gary, S.F. and Narayanaswamy, B.E., 2020. Microplastic accumulation in deep-sea sediments from the Rockall Trough. Marine pollution bulletin, 154, p.111092. \| \| Cozzolino, L., Nicastro, K.R., Zardi, G.I. and Carmen, B., 2020. Species-specific plastic accumulation in the sediment and canopy of coastal vegetated habitats. Science of The Total Environment, 723, p.138018. \| \| Cunningham, E.M., Ehlers, S.M., Dick, J.T., Sigwart, J.D., Linse, K., Dick, J.J. and Kiriakoulakis, K., 2020. High abundances of microplastic pollution in deep-sea sediments: evidence from Antarctica and the Southern Ocean. Environmental Science & Technology, 54(21), pp.13661-13671. \| \| Dahl, M., Bergman, S., Björk, M., Diaz-Almela, E., Granberg, M., Gullström, M., Leiva-Dueñas, C., Magnusson, K., Marco-Méndez, C., Piñeiro-Juncal, N. and Mateo, M.Á., 2021. A temporal record of microplastic pollution in Mediterranean seagrass soils. *Environmental Pollution*, *273*, p.116451. \| \| Dai, Z., Zhang, H., Zhou, Q., Tian, Y., Chen, T., Tu, C., Fu, C. and Luo, Y., 2018. Occurrence of microplastics in the water column and sediment in an inland sea affected by intensive anthropogenic activities. Environmental pollution, 242, pp.1557-1565. \| \| de Carvalho, D.G. and Neto, J.A.B., 2016. Microplastic pollution of the beaches of Guanabara Bay, Southeast Brazil. Ocean & coastal management, 128, pp.10-17. \| \| de Jesus Piñon-Colin, T., Rodriguez-Jimenez, R., Pastrana-Corral, M.A., Rogel-Hernandez, E. and Wakida, F.T., 2018. Microplastics on sandy beaches of the Baja California Peninsula, Mexico. Marine pollution bulletin, 131, pp.63-71. \| \| De Ruijter, V.N., Milou, A. and Costa, V., 2019. Assessment of microplastics distribution and stratification in the shallow marine sediments of Samos island, Eastern Mediterranean sea, Greece. Mediterranean Marine Science, 20(4), pp.736-744. \| \| Dekiff, J.H., Remy, D., Klasmeier, J. and Fries, E., 2014. Occurrence and spatial distribution of microplastics in sediments from Norderney. Environmental Pollution, 186, pp.248-256. \| \| De-la-Torre, G.E., Dioses-Salinas, D.C., Castro, J.M., Antay, R., Fernández, N.Y., Espinoza-Morriberón, D. and Saldaña-Serrano, M., 2020. Abundance and distribution of microplastics on sandy beaches of Lima, Peru. Marine pollution bulletin, 151, p.110877. \| \| Delvalle de Borrero, D., Fábrega Duque, J., Olmos, J., Garcés-Ordóñez, O., Amaral, S.S.G.D., Vezzone, M., de Sá Felizardo, J.P. and Meigikos dos Anjos, R., 2020. Distribution of plastic debris in the Pacific and Caribbean beaches of Panama. Air, Soil and Water Research, 13, p.1178622120920268. \| \| Deng, J., Guo, P., Zhang, X., Su, H., Zhang, Y., Wu, Y. and Li, Y., 2020. Microplastics and accumulated heavy metals in restored mangrove wetland surface sediments at Jinjiang Estuary (Fujian, China). Marine Pollution Bulletin, 159, p.111482. \| \| Díaz-Jaramillo, M., Islas, M.S. and Gonzalez, M., 2021. Spatial distribution patterns and identification of microplastics on intertidal sediments from urban and semi-natural SW Atlantic estuaries. Environmental Pollution, 273, p.116398. \| \| Dodson, G.Z., Shotorban, A.K., Hatcher, P.G., Waggoner, D.C., Ghosal, S. and Noffke, N., 2020. Microplastic fragment and fiber contamination of beach sediments from selected sites in Virginia and North Carolina, USA. Marine pollution bulletin, 151, p.110869. \| \| Doğruyol, P., Şener, M. and Balkaya, N., 2019. Determination of microplastics and large plastics in the sediments of the Golden Horn Estuary (Halic), Istanbul, Turkey. Desalin Water Treat, 172, pp.344-350. \| \| Dou, P.C., Mai, L., Bao, L.J. and Zeng, E.Y., 2021. Microplastics on beaches and mangrove sediments along the coast of South China. Marine Pollution Bulletin, 172, p.112806. \| \| Dowarah, K. and Devipriya, S.P., 2019. Microplastic prevalence in the beaches of Puducherry, India and its correlation with fishing and tourism/recreational activities. Marine pollution bulletin, 148, pp.123-133. \| \| Doyen, P., Hermabessiere, L., Dehaut, A., Himber, C., Decodts, M., Degraeve, T., Delord, L., Gaboriaud, M., Moné, P., Sacco, J. and Tavernier, E., 2019. Occurrence and identification of microplastics in beach sediments from the Hauts-de-France region. Environmental Science and Pollution Research, 26(27), pp.28010-28021. \| \| Duan, J., Han, J., Cheung, S.G., Chong, R.K.Y., Lo, C.M., Lee, F.W.F., Xu, S.J.L., Yang, Y., Tam, N.F.Y. and Zhou, H.C., 2021. How mangrove plants affect microplastic distribution in sediments of coastal wetlands: Case study in Shenzhen Bay, South China. Science of the Total Environment, 767, p.144695. \| \| Duan, J., Han, J., Zhou, H., Lau, Y. L., An, W., Wei, P., ... & Tam, N. F. Y. (2020). Development of a digestion method for determining microplastic pollution in vegetal-rich clayey mangrove sediments. Science of the Total Environment, 707, 136030. \| \| Duncan, E.M., Arrowsmith, J., Bain, C., Broderick, A.C., Lee, J., Metcalfe, K., Pikesley, S.K., Snape, R.T., van Sebille, E. and Godley, B.J., 2018. The true depth of the Mediterranean plastic problem: Extreme microplastic pollution on marine turtle nesting beaches in Cyprus. Marine pollution bulletin, 136, pp.334-340. \| \| Edo, C., Tamayo-Belda, M., Martínez-Campos, S., Martín-Betancor, K., González-Pleiter, M., Pulido-Reyes, G., García-Ruiz, C., Zapata, F., Leganés, F., Fernández-Piñas, F. and Rosal, R., 2019. Occurrence and identification of microplastics along a beach in the Biosphere Reserve of Lanzarote. Marine pollution bulletin, 143, pp.220-227. \| \| Eo, S., Hong, S.H., Song, Y.K., Lee, J., Lee, J. and Shim, W.J., 2018. Abundance, composition, and distribution of microplastics larger than 20 μm in sand beaches of South Korea. Environmental pollution, 238, pp.894-902. \| \| Eriksen M, Lebreton LCM, Carson HS, Thiel M, Moore CJ, Borerro JC, J.C., Galgani, F., Ryan, P.G. & Reisser, J. (2014) Plastic Pollution in the World's Oceans: More than 5 Trillion Plastic Pieces Weighing over 250,000 Tons Afloat at Sea. PLoS ONE 9(12): e111913. https://doi.org/10.1371/journal.pone.0111913 \| \| Esiukova, E., 2017. Plastic pollution on the Baltic beaches of Kaliningrad region, Russia. Marine pollution bulletin, 114(2), pp.1072-1080. \| \| Esiukova, E., Khatmullina, L., Lobchuk, O., Grave, A., Kileso, A., Haseler, M., Zyubin, A. and Chubarenko, I., 2020. From macro to micro: dataset on plastic contamination along and across a sandy tide-less coast (the Curonian Spit, the Baltic Sea). Data in brief, 30, p.105635. \| \| Esiukova, E., Lobchuk, O., Haseler, M. and Chubarenko, I., 2021. Microplastic contamination of sandy beaches of national parks, protected and recreational areas in southern parts of the Baltic Sea. Marine Pollution Bulletin, 173, p.113002. \| \| Esiukova, E., Zobkov, M. and Chubarenko, I., 2020. Data on microplastic contamination of the Baltic Sea bottom sediment samples in 2015–2016. Data in brief, 28, p.104887. \| \| Expósito, N., Rovira, J., Sierra, J., Folch, J. and Schuhmacher, M., 2021. Microplastics levels, size, morphology and composition in marine water, sediments and sand beaches. Case study of Tarragona coast (western Mediterranean). Science of The Total Environment, 786, p.147453. \| \| Falahudin, D., Cordova, M.R., Sun, X., Yogaswara, D., Wulandari, I., Hindarti, D. and Arifin, Z., 2020. The first occurrence, spatial distribution and characteristics of microplastic particles in sediments from Banten Bay, Indonesia. Science of the Total Environment, 705, p.135304. \| \| Fang, C., Zhang, Y., Zheng, R., Hong, F., Zhang, M., Zhang, R., Mou, J., Mu, J., Lin, L. and Bo, J., 2022. Spatio-temporal variation of microplastic pollution in the sediment from the Chukchi Sea over five years. Science of The Total Environment, 806, p.150530. \| \| Fastelli, P., Blašković, A., Bernardi, G., Romeo, T., Čižmek, H., Andaloro, F., Russo, G.F., Guerranti, C. and Renzi, M., 2016. Plastic litter in sediments from a marine area likely to become protected (Aeolian Archipelago's islands, Tyrrhenian sea). Marine pollution bulletin, 113(1-2), pp.526-529. \| \| Ferreira, M., Thompson, J., Paris, A., Rohindra, D. and Rico, C., 2020. Presence of microplastics in water, sediments and fish species in an urban coastal environment of Fiji, a Pacific small island developing state. Marine pollution bulletin, 153, p.110991. \| \| Filgueiras, A.V., Gago, J., Campillo, J.A. and León, V.M., 2019. Microplastic distribution in surface sediments along the Spanish Mediterranean continental shelf. Environmental Science and Pollution Research, 26(21), pp.21264-21273. \| \| Firdaus, M., Trihadiningrum, Y. and Lestari, P., 2020. Microplastic pollution in the sediment of Jagir Estuary, Surabaya City, Indonesia. Marine pollution bulletin, 150, p.110790. \| \| Fischer, V., Elsner, N.O., Brenke, N., Schwabe, E. and Brandt, A., 2015. Plastic pollution of the Kuril–Kamchatka Trench area (NW pacific). *Deep Sea Research Part II: Topical Studies in Oceanography*, *111*, pp.399-405. \| \| Fitri, S. and Patria, M.P., 2019, July. Microplastic contamination on anadara granosa linnaeus 1758 in pangkal babu mangrove forest area, tanjung jabung barat district, jambi. In Journal of Physics: Conference Series (Vol. 1282, No. 1, p. 012109). IOP Publishing. \| \| Fitri, S. and Patria, M.P., 2019, November. Microplastic contamination on Cerithidea obtusa (Lamarck 1822) in Pangkal Babu Mangrove Forest Area, Tanjung Jabung Barat District, Jambi. In AIP Conference Proceedings (Vol. 2168, No. 1, p. 020075). AIP Publishing LLC. \| \| Fok, L. and Cheung, P.K., 2015. Hong Kong at the Pearl River Estuary: A hotspot of microplastic pollution. Marine pollution bulletin, 99(1-2), pp.112-118. \| \| Fok, L., Cheung, P.K., Tang, G. and Li, W.C., 2017. Size distribution of stranded small plastic debris on the coast of Guangdong, South China. Environmental Pollution, 220, pp.407-412. \| \| Forsythe, C., 2017. The Quantification of Microplastics in Intertidal Sediments in the Bay of Fundy, Canada (Doctoral dissertation, Royal Roads University (Canada)). \| \| Foshtomi, M.Y., Oryan, S., Taheri, M., Bastami, K.D. and Zahed, M.A., 2019. Composition and abundance of microplastics in surface sediments and their interaction with sedimentary heavy metals, PAHs and TPH (total petroleum hydrocarbons). Marine Pollution Bulletin, 149, p.110655. \| \| Fraser, M.A., Chen, L., Ashar, M., Huang, W., Zeng, J., Zhang, C. and Zhang, D., 2020. Occurrence and distribution of microplastics and polychlorinated biphenyls in sediments from the Qiantang River and Hangzhou Bay, China. Ecotoxicology and environmental safety, 196, p.110536. \| \| Fred-Ahmadu, O.H., Ayejuyo, O.O. and Benson, N.U., 2020. Microplastics distribution and characterization in epipsammic sediments of tropical Atlantic Ocean, Nigeria. Regional Studies in Marine Science, 38, p.101365. \| \| Frias, J.P., Gago, J., Otero, V. and Sobral, P., 2016. Microplastics in coastal sediments from Southern Portuguese shelf waters. Marine environmental research, 114, pp.24-30. \| \| Gao, F., Li, J., Hu, J., Sui, B., Wang, C., Sun, C., Li, X. and Ju, P., 2021. The seasonal distribution characteristics of microplastics on bathing beaches along the coast of Qingdao, China. *Science of The Total Environment*, *783*, p.146969. \| \| Garcés-Ordóñez, O., Castillo-Olaya, V.A., Granados-Briceño, A.F., García, L.M.B. and Díaz, L.F.E., 2019. Marine litter and microplastic pollution on mangrove soils of the Ciénaga Grande de Santa Marta, Colombian Caribbean. Marine pollution bulletin, 145, pp.455-462. \| \| Garcés-Ordóñez, O., Espinosa, L.F., Cardoso, R.P., Cardozo, B.B.I. and Dos Anjos, R.M., 2020. Plastic litter pollution along sandy beaches in the Caribbean and Pacific coast of Colombia. Environmental Pollution, 267, p.115495. \| \| Garces-Ordonez, O., Saldarriaga-Velez, J.F., Espinosa-Diaz, L.F., Patino, A.D., Cusba, J., Canals, M., Mejia-Esquivia, K., Fragozo-Velasquez, L., Saenz-Arias, S., Cordoba-Meza, T. & Thiel, M. (2022). "Microplastic pollution in water, sediments and commercial fish species from Cienaga Grande de Santa Marta lagoon complex, Colombian Caribbean." Science of The Total Environment 829. \| \| Ghayebzadeh, M., Aslani, H., Taghipour, H. and Mousavi, S., 2020. Contamination of the Caspian Sea Southern coast sediments with microplastics: A marine environmental problem. Marine Pollution Bulletin, 160, p.111620. \| \| Godoy, V., Prata, J.C., Blázquez, G., Almendros, A.I., Duarte, A.C., Rocha-Santos, T., Calero, M. and Martín-Lara, M.Á., 2020. Effects of distance to the sea and geomorphological characteristics on the quantity and distribution of microplastics in beach sediments of Granada (Spain). Science of The Total Environment, 746, p.142023. \| \| González-Hernández, M., Hernandez-Sanchez, C., González-Sálamo, J., López-Darias, J. and Hernandez-Borges, J., 2020. Monitoring of meso and microplastic debris in Playa Grande beach (Tenerife, Canary Islands, Spain) during a moon cycle. Marine pollution bulletin, 150, p.110757. \| \| Goswami, P., Vinithkumar, N.V. and Dharani, G., 2021. Microplastics particles in seafloor sediments along the Arabian Sea and the Andaman Sea continental shelves: First insight on the occurrence, identification, and characterization. Marine Pollution Bulletin, 167, p.112311. \| \| Govender, J., Naidoo, T., Rajkaran, A., Cebekhulu, S. and Bhugeloo, A., 2020. Towards Characterising Microplastic Abundance, Typology and Retention in Mangrove-Dominated Estuaries. Water, 12(10), p.2802. \| \| Graca, B., Szewc, K., Zakrzewska, D., Dołęga, A. and Szczerbowska-Boruchowska, M., 2017. Sources and fate of microplastics in marine and beach sediments of the Southern Baltic Sea—a preliminary study. Environmental Science and Pollution Research, 24(8), pp.7650-7661. \| \| Gray, A.D., Wertz, H., Leads, R.R. and Weinstein, J.E., 2018. Microplastic in two South Carolina Estuaries: Occurrence, distribution, and composition. Marine pollution bulletin, 128, pp.223-233. \| \| Gupta, P., Saha, M., Rathore, C., Suneel, V., Ray, D., Naik, A., Unnikrishnan, K., Dhivya, M. and Daga, K., 2021. Spatial and seasonal variation of microplastics and possible sources in the estuarine system from central west coast of India. Environmental Pollution, 288, p.117665. \| \| Haave, M., Lorenz, C., Primpke, S. and Gerdts, G., 2019. Different stories told by small and large microplastics in sediment-first report of microplastic concentrations in an urban recipient in Norway. Marine pollution bulletin, 141, pp.501-513. \| \| Hamid, F.S., Jia, W. and Zakaria, R.M., 2020. Microplastics Abundance and Uptake by Meretrix lyrata (Hard Clam) in Mangrove Forest. Journal of Engineering & Technological Sciences, 52(3). \| \| Hansen, R.A. and Gross, A., 2019. Determination of microplastics in coastal beach sediments along Kattegat Sea, Denmark. Теоретическая и прикладная экология, (2), pp.75-82. \| \| Hengstmann, E., Tamminga, M., Vom Bruch, C. and Fischer, E.K., 2018. Microplastic in beach sediments of the Isle of Rügen (Baltic Sea)-Implementing a novel glass elutriation column. Marine pollution bulletin, 126, pp.263-274. \| \| Hidalgo-Ruz, V.,  Thiel, M. (2013) Distribution and abundance of small plastic debris on beaches in the SE Pacific (Chile): a study supported by a citizen science project Mar. Environ. Res., 87–88 , pp. 12-18 \| \| Hien, T.T., Nhon, N.T.T., Thu, V.T.M. and Nguyen, N.T., 2020. The Distribution of Microplastics in Beach Sand in Tien Giang Province and Vung Tau City, Vietnam. Journal of Engineering & Technological Sciences, 52(2). \| \| Hope, J.A., Coco, G., Ladewig, S.M. and Thrush, S.F., 2021. The distribution and ecological effects of microplastics in an estuarine ecosystem. Environmental Pollution, 288, p.117731. \| \| Hossain, M.B., Banik, P., Nur, A.A.U. and Rahman, T., 2021. Abundance and characteristics of microplastics in sediments from the world's longest natural beach, Cox's Bazar, Bangladesh. Marine Pollution Bulletin, 163, p.111956. \| \| Hosseini, R., Sayadi, M.H., Aazami, J. and Savabieasfehani, M., 2020. Accumulation and distribution of microplastics in the sediment and coastal water samples of Chabahar Bay in the Oman Sea, Iran. Marine Pollution Bulletin, 160, p.111682. \| \| Huang, Y., Xiao, X., Effiong, K., Xu, C., Su, Z., Hu, J., Jiao, S. and Holmer, M., 2021. New Insights into the Microplastic Enrichment in the Blue Carbon Ecosystem: Evidence from Seagrass Meadows and Mangrove Forests in Coastal South China Sea. Environmental Science & Technology, 55(8), pp.4804-4812. \| \| Huang, Y., Xiao, X., Xu, C., Perianen, Y.D., Hu, J. and Holmer, M., 2020. Seagrass beds acting as a trap of microplastics-Emerging hotspot in the coastal region?. Environmental Pollution, 257, p.113450. \| \| Jahan, S., Strezov, V., Weldekidan, H., Kumar, R., Kan, T., Sarkodie, S.A., He, J., Dastjerdi, B. and Wilson, S.P., 2019. Interrelationship of microplastic pollution in sediments and oysters in a seaport environment of the eastern coast of Australia. Science of the Total Environment, 695, p.133924. \| \| Jang, M., Shim, W.J., Cho, Y., Han, G.M., Song, Y.K. and Hong, S.H., 2020. A close relationship between microplastic contamination and coastal area use pattern. Water research, 171, p.115400. \| \| Jaubet, M.L., Hines, E., Elías, R. and Garaffo, G.V., 2021. Factors driving the abundance and distribution of microplastics on sandy beaches in a Southwest Atlantic seaside resort. Marine Environmental Research, 171, p.105472. \| \| Jayasiri, H.B., Purushothaman, C.S. and Vennila, A., 2013. Quantitative analysis of plastic debris on recreational beaches in Mumbai, India. Marine pollution bulletin, 77(1-2), pp.107-112. \| \| Jeyasanta, K. I., Patterson, J., Grimsditch, G., & Edward, J. P. (2020). Occurrence and characteristics of microplastics in the coral reef, sea grass and near shore habitats of Rameswaram Island, India. *Marine Pollution Bulletin*, *160*, 111674. \| \| Jeyasanta, K.I., Sathish, N., Patterson, J. and Edward, J.P., 2020. Macro-, meso-and microplastic debris in the beaches of Tuticorin district, Southeast coast of India. Marine pollution bulletin, 154, p.111055. \| \| Jiao, M., Ren, L., Wang, Y.J., Ding, C.Y., Li, T.Z., Cao, S.H., Li, R.L. & Wang, Y.H. (2022). "Mangrove forest: An important coastal ecosystem to intercept river microplastics." Environmental Research 210. \| \| Jiwarungrueangkul, T., Phaksopa, J., Sompongchaiyakul, P. and Tipmanee, D., 2021. Seasonal microplastic variations in estuarine sediments from urban canal on the west coast of Thailand: A case study in Phuket province. Marine Pollution Bulletin, 168, p.112452. \| \| Jones, J.S., Porter, A., Muñoz-Pérez, J.P., Alarcón-Ruales, D., Galloway, T.S., Godley, B.J., Santillo, D., Vagg, J. and Lewis, C., 2021. Plastic contamination of a Galapagos Island (Ecuador) and the relative risks to native marine species. Science of The Total Environment, 789, p.147704. \| \| Jones, K.L., Hartl, M.G., Bell, M.C. and Capper, A., 2020. Microplastic accumulation in a Zostera marina L. bed at Deerness Sound, Orkney, Scotland. Marine pollution bulletin, 152, p.110883. \| \| Kane, I.A., Clare, M.A., Miramontes, E., Wogelius, R., Rothwell, J.J., Garreau, P. and Pohl, F., 2020. Seafloor microplastic hotspots controlled by deep-sea circulation. Science, 368(6495), pp.1140-1145. \| \| Kanhai, D.K., Officer, R., Lyashevska, O., Thompson, R.C., O’Connor, I., 2017. Microplastic abundance, distribution and composition along a latitudinal gradient in the Atlantic ocean. Mar. Pollut. Bull. 115 (1–2), 307–314 \| \| Kannankai, M. P., Alex, R.K., Muralidharan, V.V., Nazeerkhan, N.P., Radhakrishnan, A. & Devipriya, S.P. (2022) "Urban mangrove ecosystems are under severe threat from microplastic pollution: a case study from Mangalavanam, Kerala, India." Environmental Science and Pollution Research. \| \| Karkanorachaki, K., Kiparissis, S., Kalogerakis, G.C., Yiantzi, E., Psillakis, E. and Kalogerakis, N., 2018. Plastic pellets, meso-and microplastics on the coastline of Northern Crete: Distribution and organic pollution. Marine pollution bulletin, 133, pp.578-589. \| \| Karthik, R., Robin, R.S., Purvaja, R., Ganguly, D., Anandavelu, I., Raghuraman, R., Hariharan, G., Ramakrishna, A. and Ramesh, R., 2018. Microplastics along the beaches of southeast coast of India. Science of The Total Environment, 645, pp.1388-1399. \| \| Kazour, M., Jemaa, S., Issa, C., Khalaf, G. and Amara, R., 2019. Microplastics pollution along the Lebanese coast (Eastern Mediterranean Basin): Occurrence in surface water, sediments and biota samples. Science of the Total Environment, 696, p.133933. \| \| Kerubo, J.O., Muthumbi, A.W.N., Onyari, J.M., Robertson-Andersson, D. and Kimani, E., 2021. Microplastics pollution in the sediments of creeks and estuaries of Kenya, western Indian Ocean. African Journal of Marine Science, 43(3), pp.337-352. \| \| Khan, M.B. and Prezant, R.S., 2018. Microplastic abundances in a mussel bed and ingestion by the ribbed marsh mussel Geukensia demissa. Marine pollution bulletin, 130, pp.67-75. \| \| Khuyen, V.T.K., Le, D.V., Fischer, A.R. and Dornack, C., 2021. Comparison of Microplastic Pollution in Beach Sediment and Seawater at UNESCO Can Gio Mangrove Biosphere Reserve. Global Challenges, 5(11), p.2100044. \| \| Kim, I.S., Chae, D.H., Kim, S.K., Choi, S. and Woo, S.B., 2015. Factors influencing the spatial variation of microplastics on high-tidal coastal beaches in Korea. Archives of environmental contamination and toxicology, 69(3), pp.299-309. \| \| Koongolla, J.B., Andrady, A.L., Kumara, P.T.P. and Gangabadage, C.S., 2018. Evidence of microplastics pollution in coastal beaches and waters in southern Sri Lanka. Marine pollution bulletin, 137, pp.277-284. \| \| Kor, K., Ghazilou, A. and Ershadifar, H., 2020. Microplastic pollution in the littoral sediments of the northern part of the Oman Sea. Marine pollution bulletin, 155, p.111166. \| \| Korez, Š., Gutow, L. and Saborowski, R., 2019. Microplastics at the strandlines of Slovenian beaches. Marine pollution bulletin, 145, pp.334-342. \| \| Kreitsberg, R., Raudna-Kristoffersen, M., Heinlaan, M., Ward, R., Visnapuu, M., Kisand, V., Meitern, R., Kotta, J. and Tuvikene, A., 2021. Seagrass beds reveal high abundance of microplastic in sediments: A case study in the Baltic Sea. *Marine Pollution Bulletin*, *168*, p.112417. \| \| Kumar, A.S. and Varghese, G.K., 2021. Microplastic pollution of Calicut beach-Contributing factors and possible impacts. Marine Pollution Bulletin, 169, p.112492. \| \| Kunz, A., Walther, B.A., Löwemark, L. and Lee, Y.C., 2016. Distribution and quantity of microplastic on sandy beaches along the northern coast of Taiwan. Marine Pollution Bulletin, 111(1-2), pp.126-135. \| \| L. Zhang, S. Zhang, Y. Wang, K. Yu, R. Li, The spatial distribution of microplastic in the sands of a coral reef island in the South China Sea: comparisons of the fringing reef and atoll. Sci. Total Environ., 688 (2019), pp. 780-786 \| \| La Daana, K. K., Johansson, C., Frias, J. P. G. L., Gardfeldt, K., Thompson, R. C., & O’Connor, I. (2019). Deep sea sediments of the Arctic Central Basin: a potential sink for microplastics. Deep Sea Research Part I: Oceanographic Research Papers, 145, 137-142. \| \| Laglbauer, B.J., Franco-Santos, R.M., Andreu-Cazenave, M., Brunelli, L., Papadatou, M., Palatinus, A., Grego, M. and Deprez, T., 2014. Macrodebris and microplastics from beaches in Slovenia. Marine pollution bulletin, 89(1-2), pp.356-366. \| \| Lechthaler, S., Schwarzbauer, J., Reicherter, K., Stauch, G. and Schüttrumpf, H., 2020. Regional study of microplastics in surface waters and deep sea sediments south of the Algarve Coast. Regional Studies in Marine Science, 40, p.101488. \| \| Lee, J., Hong, S., Song, Y.K., Hong, S.H., Jang, Y.C., Jang, M., Heo, N.W., Han, G.M., Lee, M.J., Kang, D. and Shim, W.J., 2013. Relationships among the abundances of plastic debris in different size classes on beaches in South Korea. Marine pollution bulletin, 77(1-2), pp.349-354. \| \| Lee, J., Lee, J.S., Jang, Y.C., Hong, S.Y., Shim, W.J., Song, Y.K., Hong, S.H., Jang, M., Han, G.M., Kang, D. and Hong, S., 2015. Distribution and size relationships of plastic marine debris on beaches in South Korea. Archives of environmental contamination and toxicology, 69(3), pp.288-298. \| \| Lefebvre, C., Rojas, I.J., Lasserre, J., Villette, S., Lecomte, S., Cachot, J. and Morin, B., 2021. Stranded in the high tide line: Spatial and temporal variability of beached microplastics in a semi-enclosed embayment (Arcachon, France). Science of The Total Environment, 797, p.149144. \| \| Li, J., Huang, W., Xu, Y., Jin, A., Zhang, D. and Zhang, C., 2020. Microplastics in sediment cores as indicators of temporal trends in microplastic pollution in Andong salt marsh, Hangzhou Bay, China. Regional Studies in Marine Science, 35, p.101149. \| \| Li, R. L., Wei, C., Jiao, M., Wang, Y. & Sun, H. (2022). "Mangrove leaves: An undeniably important sink of MPs from tidal water and air." Journal of Hazardous Materials 426. \| \| Li, R., Yu, L., Chai, M., Wu, H. and Zhu, X., 2020. The distribution, characteristics and ecological risks of microplastics in the mangroves of Southern China. Science of the Total Environment, 708, p.135025. \| \| Li, R., Zhang, L., Xue, B. and Wang, Y., 2019. Abundance and characteristics of microplastics in the mangrove sediment of the semi-enclosed Maowei Sea of the south China sea: New implications for location, rhizosphere, and sediment compositions. Environmental Pollution, 244, pp.685-692. \| \| Li, R., Zhang, S., Zhang, L., Yu, K., Wang, S. and Wang, Y., 2020. Field study of the microplastic pollution in sea snails (Ellobium chinense) from mangrove forest and their relationships with microplastics in water/sediment located on the north of Beibu Gulf. Environmental Pollution, 263, p.114368. \| \| Li, Y., Lu, Z., Zheng, H., Wang, J. and Chen, C., 2020. Microplastics in surface water and sediments of Chongming Island in the Yangtze Estuary, China. Environmental Sciences Europe, 32(1), pp.1-12. \| \| Li, Y., Zhang, Y., Chen, G., Xu, K., Gong, H., Huang, K., Yan, M. and Wang, J., 2021. Microplastics in Surface Waters and Sediments from Guangdong Coastal Areas, South China. Sustainability, 13(5), p.2691. \| \| Lim, Y. C., Chen, C. W., Cheng, Y. R., Chen, C. F., & Dong, C. D. (2022). Impacts of microplastics on scleractinian corals nearshore Liuqiu Island southwestern Taiwan. *Environmental Pollution*, *306*, 119371. \| \| Lima, A. R., Ferreira, G. V., Barrows, A. P., Christiansen, K. S., Treinish, G., & Toshack, M. C. (2021). Global patterns for the spatial distribution of floating microfibers: Arctic Ocean as a potential accumulation zone. Journal of Hazardous Materials, 403, 123796. \| \| Lin, J., Xu, X.M., Yue, B.Y., Xu, X.P., Liu, J.Z., Zhu, Q. and Wang, J.H., 2021. Multidecadal records of microplastic accumulation in the coastal sediments of the East China Sea. Chemosphere, 270, p.128658. \| \| Liu, K., Zhang, Z., Wu, H., Wang, J., Wang, R., Zhang, T., Feng, Z. and Li, D., 2021. Accumulation of microplastics in a downstream area of a semi-enclosed bay: Implications of input from coastal currents. Science of The Total Environment, p.148280. \| \| Liu, X., Liu, H., Chen, L. and Wang, X., 2022. Ecological interception effect of mangroves on microplastics. Journal of Hazardous Materials, 423, p.127231. \| \| Lo, H.S., Xu, X., Wong, C.Y. and Cheung, S.G., 2018. Comparisons of microplastic pollution between mudflats and sandy beaches in Hong Kong. Environmental Pollution, 236, pp.208-217. \| \| Lorenz, C., Roscher, L., Meyer, M.S., Hildebrandt, L., Prume, J., Löder, M.G., Primpke, S. and Gerdts, G., 2019. Spatial distribution of microplastics in sediments and surface waters of the southern North Sea. Environmental Pollution, 252, pp.1719-1729. \| \| Lots, F.A., Behrens, P., Vijver, M.G., Horton, A.A. and Bosker, T., 2017. A large-scale investigation of microplastic contamination: abundance and characteristics of microplastics in European beach sediment. Marine pollution bulletin, 123(1-2), pp.219-226. \| \| Lozoya, J.P., de Mello, F.T., Carrizo, D., Weinstein, F., Olivera, Y., Cedrés, F., Pereira, M. and Fossati, M., 2016. Plastics and microplastics on recreational beaches in Punta del Este (Uruguay): unseen critical residents?. Environmental Pollution, 218, pp.931-941. \| \| Lusher, A.L., Burke, A., O’Connor, I., Officer, R., 2014. Microplastic pollution in the Northeast Atlantic Ocean: validated and opportunistic sampling. Mar. Pollut. Bull. 88 (1–2), 325–333 \| \| Maes, T., Van der Meulen, M.D., Devriese, L.I., Leslie, H.A., Huvet, A., Frère, L., Robbens, J. and Vethaak, A.D., 2017. Microplastics baseline surveys at the water surface and in sediments of the North-East Atlantic. Frontiers in Marine Science, 4, p.135. \| \| Maghsodian, Z., Sanati, A.M., Ramavandi, B., Ghasemi, A. and Sorial, G.A., 2021. Microplastics accumulation in sediments and Periophthalmus waltoni fish, mangrove forests in southern Iran. Chemosphere, 264, p.128543. \| \| Maharana, D., Saha, M., Dar, J.Y., Rathore, C., Sreepada, R.A., Xu, X.R., Koongolla, J.B. and Li, H.X., 2020. Assessment of micro and macroplastics along the west coast of India: abundance, distribution, polymer type and toxicity. Chemosphere, 246, p.125708. \| \| Manbohi, A., Mehdinia, A., Rahnama, R., Dehbandi, R. and Hamzehpour, A., 2021. Spatial distribution of microplastics in sandy beach and inshore-offshore sediments of the southern Caspian Sea. Marine Pollution Bulletin, 169, p.112578. \| \| Marei, I.C.A., Saleh, F.I.E., Manullang, C.Y., Soamole, A. and Rehalat, I., 2021, December. Occurrence and distribution of microplastics in the beach sediment of Anday Beach, West Papua (Indonesia). In IOP Conference Series: Earth and Environmental Science (Vol. 944, No. 1, p. 012070). IOP Publishing. \| \| Martin, C., Baalkhuyur, F., Valluzzi, L., Saderne, V., Cusack, M., Almahasheer, H., Krishnakumar, P.K., Rabaoui, L., Qurban, M.A., Arias-Ortiz, A. and Masqué, P., 2020. Exponential increase of plastic burial in mangrove sediments as a major plastic sink. Science advances, 6(44), p.eaaz5593. \| \| Martinelli Filho, J.E. and Monteiro, R.C.P., 2019. Widespread microplastics distribution at an Amazon macrotidal sandy beach. Marine pollution bulletin, 145, pp.219-223. \| \| Martins, J., & Sobral, P. (2011). Plastic marine debris on the Portuguese coastline: a matter of size?. *Marine pollution bulletin*, *62*(12), 2649-2653. \| \| Masiá, P., Ardura, A., Gaitán, M., Gerber, S., Rayon-Viña, F. and Garcia-Vazquez, E., 2021. Maritime ports and beach management as sources of coastal macro-, meso-, and microplastic pollution. Environmental Science and Pollution Research, pp.1-10. \| \| Mataji, A., Taleshi, M.S. and Balimoghaddas, E., 2020. Distribution and characterization of microplastics in surface waters and the Southern Caspian Sea Coasts sediments. Archives of environmental contamination and toxicology, 78(1), pp.86-93. \| \| Matsuguma, Y., Takada, H., Kumata, H., Kanke, H., Sakurai, S., Suzuki, T., Itoh, M., Okazaki, Y., Boonyatumanond, R., Zakaria, M.P. and Weerts, S., 2017. Microplastics in sediment cores from Asia and Africa as indicators of temporal trends in plastic pollution. Archives of environmental contamination and toxicology, 73(2), pp.230-239. \| \| Mayoma, B.S., Sørensen, C., Shashoua, Y. and Khan, F.R., 2020. Microplastics in beach sediments and cockles (Anadara antiquata) along the Tanzanian coastline. Bulletin of Environmental Contamination and Toxicology, 105(4), pp.513-521. \| \| Mbedzi, R., Cuthbert, R.N., Wasserman, R.J., Murungweni, F.M. and Dalu, T., 2020. Spatiotemporal variation in microplastic contamination along a subtropical reservoir shoreline. Environmental Science and Pollution Research, 27(19), pp.23880-23887. \| \| McEachern, K., Alegria, H., Kalagher, A.L., Hansen, C., Morrison, S. and Hastings, D., 2019. Microplastics in Tampa Bay, Florida: abundance and variability in estuarine waters and sediments. Marine pollution bulletin, 148, pp.97-106. \| \| Mendes, A.M., Golden, N., Bermejo, R. and Morrison, L., 2021. Distribution and abundance of microplastics in coastal sediments depends on grain size and distance from sources. Marine Pollution Bulletin, 172, p.112802. \| \| Mengatto, M. F. and R. H. Nagai (2022). "A first assessment of microplastic abundance in sandy beach sediments of the Paranagu ' a Estuarine Complex, South Brazil (RAMSAR site)." Marine Pollution Bulletin 177. \| \| Miller, M. E., Motti, C. A., Hamann, M., & Kroon, F. J. (2023). Assessment of microplastic bioconcentration, bioaccumulation and biomagnification in a simple coral reef food web. *Science of The Total Environment*, *858*, 159615. \| \| Missawi, O., Bousserrhine, N., Belbekhouche, S., Zitouni, N., Alphonse, V., Boughattas, I. and Banni, M., 2020. Abundance and distribution of small microplastics (≤ 3 μm) in sediments and seaworms from the Southern Mediterranean coasts and characterisation of their potential harmful effects. Environmental Pollution, 263, p.114634. \| \| Mistri, M., Infantini, V., Scoponi, M., Granata, T., Moruzzi, L., Massara, F., De Donati, M. and Munari, C., 2017. Small plastic debris in sediments from the Central Adriatic Sea: Types, occurrence and distribution. Marine pollution bulletin, 124(1), pp.435-440. \| \| Mistri, M., Scoponi, M., Granata, T., Moruzzi, L., Massara, F. and Munari, C., 2020. Types, occurrence and distribution of microplastics in sediments from the northern Tyrrhenian Sea. Marine pollution bulletin, 153, p.111016. \| \| Mistri, M., Scoponi, M., Sfriso, A.A., Munari, C., Curiotto, M., Sfriso, A., Orlando-Bonaca, M. and Lipej, L., 2021. Microplastic Contamination in Protected Areas of the Gulf of Venice. Water, Air, & Soil Pollution, 232(9), pp.1-9. \| \| Monteiro, R.C.P., Sul, J.A.I.D. and Costa, M.F., 2020. Small microplastics on beaches of Fernando de Noronha Island, Tropical Atlantic Ocean. Ocean and Coastal Research, 68. \| \| Mu, J., Qu, L., Jin, F., Zhang, S., Fang, C., Ma, X., Zhang, W., Huo, C., Cong, Y. and Wang, J., 2019. Abundance and distribution of microplastics in the surface sediments from the northern Bering and Chukchi Seas. Environmental Pollution, 245, pp.122-130. \| \| Munari, C., Scoponi, M. and Mistri, M., 2017. Plastic debris in the Mediterranean Sea: Types, occurrence and distribution along Adriatic shorelines. Waste Management, 67, pp.385-391. \| \| Nabizadeh, R., Sajadi, M., Rastkari, N. and Yaghmaeian, K., 2019. Microplastic pollution on the Persian Gulf shoreline: A case study of Bandar Abbas city, Hormozgan Province, Iran. Marine pollution bulletin, 145, pp.536-546. \| \| Naidoo, T., Glassom, D. and Smit, A.J., 2015. Plastic pollution in five urban estuaries of KwaZulu-Natal, South Africa. Marine pollution bulletin, 101(1), pp.473-480. \| \| Naji, A., Esmaili, Z. and Khan, F.R., 2017. Plastic debris and microplastics along the beaches of the Strait of Hormuz, Persian Gulf. Marine pollution bulletin, 114(2), pp.1057-1062. \| \| Naji, A., Esmaili, Z., Mason, S.A. and Vethaak, A.D., 2017. The occurrence of microplastic contamination in littoral sediments of the Persian Gulf, Iran. Environmental Science and pollution research, 24(25), pp.20459-20468. \| \| Naji, A., Nuri, M., Amiri, P. and Niyogi, S., 2019. Small microplastic particles (S-MPPs) in sediments of mangrove ecosystem on the northern coast of the Persian Gulf. Marine pollution bulletin, 146, pp.305-311. \| \| Nel, H.A. and Froneman, P.W., 2015. A quantitative analysis of microplastic pollution along the south-eastern coastline of South Africa. Marine pollution bulletin, 101(1), pp.274-279. \| \| Nel, H.A., Smith, G.H.S., Harmer, R., Sykes, R., Schneidewind, U., Lynch, I. and Krause, S., 2020. Citizen science reveals microplastic hotspots within tidal estuaries and the remote Scilly Islands, United Kingdom. Marine Pollution Bulletin, 161, p.111776. \| \| Nematollahi, M.J., Moore, F., Keshavarzi, B., Vogt, R.D., Saravi, H.N. and Busquets, R., 2020. Microplastic particles in sediments and waters, south of Caspian Sea: frequency, distribution, characteristics, and chemical composition. Ecotoxicology and Environmental Safety, 206, p.111137. \| \| Nguyen, Q.A.T., Nguyen, H.N.Y., Strady, E., Nguyen, Q.T. and Trinh-Dang, M., 2020. Characteristics of microplastics in shoreline sediments from a tropical and urbanized beach (Da Nang, Vietnam). Marine pollution bulletin, 161, p.111768. \| \| Nor, N.H.M. and Obbard, J.P., 2014. Microplastics in Singapore’s coastal mangrove ecosystems. Marine pollution bulletin, 79(1-2), pp.278-283. \| \| Osorio, E. D., Tanchuling, M. A. N., & Diola, M. B. L. D. (2021). Microplastics occurrence in surface waters and sediments in five river mouths of Manila Bay. Frontiers in Environmental Science, 9, 719274. \| \| Paes, E. D., Gloaguen, T.V., Silva, H.D.D., Duarte, T.S., de Almeida, M.D., Costa, O.D.V., Bomfim, M.R. & Santos, J.A.G. (2022). "Widespread microplastic pollution in mangrove soils of Todos os Santos Bay, northern Brazil." Environmental Research 210. \| \| Pagter, E., Frias, J., Kavanagh, F. and Nash, R., 2020. Varying levels of microplastics in benthic sediments within a shallow coastal embayment. Estuarine, Coastal and Shelf Science, 243, p.106915. \| \| Paler, M.K.O., Malenab, M.C.T., Maralit, J.R. and Nacorda, H.M., 2019. Plastic waste occurrence on a beach off southwestern Luzon, Philippines. Marine pollution bulletin, 141, pp.416-419. \| \| Patchaiyappan, A., Ahmed, S.Z., Dowarah, K., Jayakumar, S. and Devipriya, S.P., 2020. Occurrence, distribution and composition of microplastics in the sediments of South Andaman beaches. Marine Pollution Bulletin, 156, p.111227. \| \| Patchaiyappan, A., ZakiAhmed, S., Dowarah, K., Khadanga, S.S., Singh, T., Jayakumar, S., Thirunavukkarasu, C. and Devipriya, S.P., 2021. Prevalence of microplastics in the sediments of Odisha beaches, southeastern coast of India. Marine Pollution Bulletin, 167, p.112265. \| \| Patterson, J., Jeyasanta, K. I., Sathish, N., Edward, J. P., & Booth, A. M. (2020). Microplastic and heavy metal distributions in an Indian coral reef ecosystem. *Science of the Total Environment*, *744*, 140706. \| \| Patti, T. B., Fobert, E. K., Reeves, S. E., & da Silva, K. B. (2020). Spatial distribution of microplastics around an inhabited coral island in the Maldives, Indian Ocean. *Science of The Total Environment*, *748*, 141263. \| \| Pazos, R.S., Amalvy, J., Cochero, J., Pecile, A. and Gómez, N., 2021. Temporal patterns in the abundance, type and composition of microplastics on the coast of the Río de la Plata estuary. Marine Pollution Bulletin, 168, p.112382. \| \| Peng, G., Zhu, B., Yang, D., Su, L., Shi, H. and Li, D., 2017. Microplastics in sediments of the Changjiang Estuary, China. Environmental Pollution, 225, pp.283-290. \| \| Peng, X., M. Chen, S. Chen, S. Dasgupta, H. Xu, K. Ta, M. Du, J. Li, Z. Guo, and S. Bai. "Microplastics contaminate the deepest part of the world’s ocean." Geochemical Perspectives Letters 9, no. 1 (2018): 1-5. \| \| Pérez-Alvelo, K.M., Llegus, E.M., Forestier-Babilonia, J.M., Elías-Arroyo, C.V., Pagán-Malavé, K.N., Bird-Rivera, G.J. and Rodríguez-Sierra, C.J., 2021. Microplastic pollution on sandy beaches of Puerto Rico. Marine Pollution Bulletin, 164, p.112010. \| \| Pervez, R., Wang, Y., Mahmood, Q. and Jattak, Z., 2020. Stereomicroscopic and Fourier Transform Infrared (FTIR) Spectroscopic Characterization of the Abundance, Distribution and Composition of Microplastics in the Beaches of Qingdao, China. Analytical Letters, 53(18), pp.2960-2977. \| \| Phuong, N.N., Poirier, L., Lagarde, F., Kamari, A. and Zalouk-Vergnoux, A., 2018. Microplastic abundance and characteristics in French Atlantic coastal sediments using a new extraction method. Environmental Pollution, 243, pp.228-237. \| \| Piazzolla, D., Cafaro, V., de Lucia, G.A., Mancini, E., Scanu, S., Bonamano, S., Piermattei, V., Vianello, A., Della Ventura, G. and Marcelli, M., 2020. Microlitter pollution in coastal sediments of the northern Tyrrhenian Sea, Italy: Microplastics and fly-ash occurrence and distribution. Estuarine, Coastal and Shelf Science, 241, p.106819. \| \| Pinheiro, L. M., Britz, L.M.K., Agostini, V.O., Perez-Parada, A., Garcia-Rodriguez, F., Galloway, T.S. & Pinho, G.L.L. (2022). "Salt marshes as the final watershed fate for meso- and microplastic contamination: A case study from Southern Brazil." Science of The Total Environment 838. \| \| Plata, M.D.C.A., Hernández, C.R., Sempoal, M.A.A., Acosta, A.F.G. and Galindo, J.E.H., 2021. Anthropogenic Microparticles: Coastal Distribution in the Southern Mexican Pacific Coast. Thalassas: An International Journal of Marine Sciences, 37(2), pp.917-926. \| \| Plee, T.A. and Pomory, C.M., 2020. Microplastics in sandy environments in the Florida Keys and the panhandle of Florida, and the ingestion by sea cucumbers (Echinodermata: Holothuroidea) and sand dollars (Echinodermata: Echinoidea). Marine Pollution Bulletin, 158, p.111437. \| \| Pozdnyakov, S.R., Ivanova, E.V., Guzeva, A.V., Shalunova, E.P., Martinson, K.D. and Tikhonova, D.A., 2020. Studying the concentration of microplastic particles in water, bottom sediments and subsoils in the coastal area of the Neva Bay, the Gulf of Finland. Water resources, 47(4), pp.599-607. \| \| Pradit, S., Noppradit, P., Loh, P.S., Nitiratsuwan, T., Le, T.P.Q., Oeurng, C., Mohamed, C.A., Lee, C.W., Lu, X.X., Anshari, G.Z., Kandasamy, S. & Wang, J.J. (2022). "The Occurrence of Microplastics in Sediment Cores from Two Mangrove Areas in Southern Thailand." JOURNAL OF MARINE SCIENCE AND ENGINEERING 10(3). \| \| Prata, J.C., Reis, V., Paço, A., Martins, P., Cruz, A., da Costa, J.P., Duarte, A.C. and Rocha-Santos, T., 2020. Effects of spatial and seasonal factors on the characteristics and carbonyl index of (micro) plastics in a sandy beach in Aveiro, Portugal. Science of The Total Environment, 709, p.135892. \| \| Qi, H., Fu, D., Wang, Z., Gao, M. and Peng, L., 2020. Microplastics occurrence and spatial distribution in seawater and sediment of Haikou Bay in the northern South China Sea. Estuarine, Coastal and Shelf Science, 239, p.106757. \| \| Qiu, Q., Peng, J., Yu, X., Chen, F., Wang, J. and Dong, F., 2015. Occurrence of microplastics in the coastal marine environment: first observation on sediment of China. Marine Pollution Bulletin, 98(1-2), pp.274-280. \| \| Rahman, S.M.A., Robin, G.S., Momotaj, M., Uddin, J. and Siddique, M.A.M., 2020. Occurrence and spatial distribution of microplastics in beach sediments of Cox's Bazar, Bangladesh. Marine Pollution Bulletin, 160, p.111587. \| \| Ramasamy, E.V., Sruthy, S., Harit, A.K., Mohan, M. and Binish, M.B., 2021. Microplastic pollution in the surface sediment of Kongsfjorden, Svalbard, Arctic. Marine Pollution Bulletin, 173, p.112986. \| \| Ramirez, M.M.B., Caamal, R.D. and von Osten, J.R., 2019. Occurrence and seasonal distribution of microplastics and phthalates in sediments from the urban channel of the Ria and coast of Campeche, Mexico. Science of the Total Environment, 672, pp.97-105. \| \| Rangel-Buitrago, N., Arroyo-Olarte, H., Trilleras, J., Arana, V.A., Mantilla-Barbosa, E., Gracia, A., Mendoza, A.V., Neal, W.J., Williams, A.T. and Micallef, A., 2021. Microplastics pollution on colombian Central Caribbean beaches. Marine Pollution Bulletin, 170, p.112685. \| \| Rapp, J., Herrera, A., Martinez, I., Raymond, E., Santana, Á. and Gómez, M., 2020. Study of plastic pollution and its potential sources on Gran Canaria Island beaches (Canary Islands, Spain). Marine pollution bulletin, 153, p.110967. \| \| Rasta, M., Rahimibashar, M.R., Torabi Jafroudi, H., Fakheri, S., Tagheipour Kouhbane, S. and Taridashti, F., 2021. Microplastics in Sediments of Southwest Caspian Sea: Characteristics, Distribution and Seasonal Variability. Soil and Sediment Contamination: An International Journal, pp.1-15. \| \| Renzi, M. and Blašković, A., 2020. Chemical fingerprint of plastic litter in sediments and holothurians from Croatia: assessment & relation to different environmental factors. Marine pollution bulletin, 153, p.110994. \| \| Renzi, M., Blašković, A., Fastelli, P., Marcelli, M., Guerranti, C., Cannas, S., Barone, L. and Massara, F., 2018. Is the microplastic selective according to the habitat? Records in amphioxus sands, Mäerl bed habitats and Cymodocea nodosa habitats. Marine pollution bulletin, 130, pp.179-183. \| \| Retama, I., Jonathan, M.P., Shruti, V.C., Velumani, S., Sarkar, S.K., Roy, P.D. and Rodríguez-Espinosa, P.F., 2016. Microplastics in tourist beaches of Huatulco Bay, Pacific coast of southern Mexico. Marine pollution bulletin, 113(1-2), pp.530-535. \| \| Rey, S.F., Franklin, J. and Rey, S.J., 2021. Microplastic pollution on island beaches, Oahu, Hawaii. Plos one, 16(2), p.e0247224. \| \| Rios-Mendoza, L.M., Ontiveros-Cuadras, J.F., Leon-Vargas, D., Ruiz-Fernández, A.C., Rangel-García, M., Pérez-Bernal, L.H. and Sanchez-Cabeza, J.A., 2021. Microplastic contamination and fluxes in a touristic area at the SE Gulf of California. Marine Pollution Bulletin, 170, p.112638. \| \| Rose, D., & Webber, M. (2019). Characterization of microplastics in the surface waters of Kingston Harbour. Science of the Total Environment, 664, 753-760. \| \| Saliu, F., Montano, S., Garavaglia, M.G., Lasagni, M., Seveso, D. and Galli, P., 2018. Microplastic and charred microplastic in the Faafu Atoll, Maldives. Marine pollution bulletin, 136, pp.464-471. \| \| Sanchez-Vidal, A., Thompson, R.C., Canals, M. and De Haan, W.P., 2018. The imprint of microfibres in southern European deep seas. PloS one, 13(11), p.e0207033. \| \| Sandre, F., Dromard, C. R., Le Menach, K., Bouchon-Navaro, Y., Cordonnier, S., Tapie, N., ... & Bouchon, C. (2019). Detection of adsorbed chlordecone on microplastics in marine sediments in Guadeloupe: a preliminary study. *Gulf and Caribbean Research*, *30*(1), GCFI-8. \| \| Sathish, M.N., Jeyasanta, K.I. and Patterson, J., 2020. Monitoring of microplastics in the clam Donax cuneatus and its habitat in Tuticorin coast of Gulf of Mannar (GoM), India. Environmental Pollution, 266, p.115219. \| \| Sathish, N., Jeyasanta, K.I. and Patterson, J., 2019. Abundance, characteristics and surface degradation features of microplastics in beach sediments of five coastal areas in Tamil Nadu, India. Marine pollution bulletin, 142, pp.112-118. \| \| Sawalman, R., Werorilangi, S., Ukkas, M., Mashoreng, S., Yasir, I. and Tahir, A., 2021, May. Microplastic abundance in sea urchins (Diadema setosum) from seagrass beds of Barranglompo Island, Makassar, Indonesia. In IOP Conference Series: Earth and Environmental Science (Vol. 763, No. 1, p. 012057). IOP Publishing. \| \| Sayed, A.E.D.H., Hamed, M., Badrey, A.E., Ismail, R.F., Osman, Y.A., Osman, A.G. and Soliman, H.A., 2021. Microplastic distribution, abundance, and composition in the sediments, water, and fishes of the Red and Mediterranean seas, Egypt. Marine Pollution Bulletin, 173, p.112966. \| \| Sayogo, B.H., Patria, M.P. and Takarina, N.D., 2020, April. The density of microplastic in sea cucumber (Holothuria sp.) and sediment at Tidung Besar and Bira Besar island, Jakarta. In Journal of Physics: Conference Series (Vol. 1524, No. 1, p. 012064). IOP Publishing. \| \| Schröder, K., Kossel, E. and Lenz, M., 2021. Microplastic abundance in beach sediments of the Kiel Fjord, Western Baltic Sea. Environmental Science and Pollution Research, 28(21), pp.26515-26528. \| \| Scopetani, C., Chelazzi, D., Martellini, T., Pellinen, J., Ugolini, A., Sarti, C. and Cincinelli, A., 2021. Occurrence and characterization of microplastic and mesoplastic pollution in the Migliarino San Rossore, Massaciuccoli Nature Park (Italy). Marine Pollution Bulletin, 171, p.112712. \| \| Scott, N., Porter, A., Santillo, D., Simpson, H., Lloyd-Williams, S. and Lewis, C., 2019. Particle characteristics of microplastics contaminating the mussel Mytilus edulis and their surrounding environments. Marine pollution bulletin, 146, pp.125-133. \| \| Şener, M., Doğruyol, P. and Balkaya, N., 2019. Microplastic pollution in the Black Sea Coast of the Anatolian side of Istanbul, Turkey. \| \| Singh, V., Chakraborty, S. and Chaudhuri, P., 2021. Quantification and polymer characterization of sediment microplastics along the Golden beach, Puri, India. \| \| SK, A. and Varghese, G.K., 2020. Environmental forensic analysis of the microplastic pollution at “Nattika” Beach, Kerala Coast, India. Environmental Forensics, 21(1), pp.21-36. \| \| Stolte, A., Forster, S., Gerdts, G. and Schubert, H., 2015. Microplastic concentrations in beach sediments along the German Baltic coast. Marine Pollution Bulletin, 99(1-2), pp.216-229. \| \| Su, L., Sharp, S.M., Pettigrove, V.J., Craig, N.J., Nan, B., Du, F. and Shi, H., 2020. Superimposed microplastic pollution in a coastal metropolis. Water research, 168, p.115140. \| \| Sui, Q., Zhang, L., Xia, B., Chen, B., Sun, X., Zhu, L., Wang, R. and Qu, K., 2020. Spatiotemporal distribution, source identification and inventory of microplastics in surface sediments from Sanggou Bay, China. Science of the Total Environment, 723, p.138064. \| \| Sun, X., Wang, T., Chen, B., Booth, A.M., Liu, S., Wang, R., Zhu, L., Zhao, X., Qu, K. and Xia, B., 2021. Factors influencing the occurrence and distribution of microplastics in coastal sediments: From source to sink. Journal of Hazardous Materials, 410, p.124982. \| \| Sundar, S., Chokkalingam, L., Roy, P.D. and Usha, T., 2021. Estimation of microplastics in sediments at the southernmost coast of India (Kanyakumari). Environmental Science and Pollution Research, 28(15), pp.18495-18500. \| \| Sunitha, T.G., Monisha, V., Sivanesan, S., Vasanthy, M., Prabhakaran, M., Omine, K., Sivasankar, V. and Darchen, A., 2021. Micro-plastic pollution along the Bay of Bengal coastal stretch of Tamil Nadu, South India. Science of the Total Environment, 756, p.144073. \| \| Suresh, A., Vijayaraghavan, G., Saranya, K.S., Neethu, K.V. and Aneesh, B., 2020. Microplastics distribution and contamination from the Cochin coastal zone, India. Regional Studies in Marine Science, 40, p.101533. \| \| Tahir, A., Samawi, M.F., Sari, K., Hidayat, R., Nimzet, R., Wicaksono, E.A., Asrul, L. and Werorilangi, S., 2019, October. Studies on microplastic contamination in seagrass beds at Spermonde Archipelago of Makassar Strait, Indonesia. In Journal of Physics: Conference Series (Vol. 1341, No. 2, p. 022008). IOP Publishing. \| \| Tahir, A., Soeprapto, D.A., Sari, K., Wicaksono, E.A. and Werorilangi, S., 2020, September. Microplastic assessment in seagrass ecosystem at Kodingareng Lompo Island of Makassar city. In IOP Conference Series: Earth and Environmental Science (Vol. 564, No. 1, p. 012032). IOP Publishing. \| \| Taïbi, N.E., Bentaallah, M.E.A., Alomar, C., Compa, M. and Deudero, S., 2021. Micro-and macro-plastics in beach sediment of the Algerian western coast: First data on distribution, characterization, and source. Marine Pollution Bulletin, 165, p.112168. \| \| Tajwar, M., Gazi, M.Y. and Saha, S.K., 2021. Characterization and Spatial Abundance of Microplastics in the Coastal Regions of Cox’s Bazar, Bangladesh: An Integration of Field, Laboratory, and GIS Techniques. Soil and Sediment Contamination: An International Journal, pp.1-25. \| \| Tang, G., Liu, M., Zhou, Q., He, H., Chen, K., Zhang, H., Hu, J., Huang, Q., Luo, Y., Ke, H. and Chen, B., 2018. Microplastics and polycyclic aromatic hydrocarbons (PAHs) in Xiamen coastal areas: implications for anthropogenic impacts. Science of the Total Environment, 634, pp.811-820. \| \| Tanhua T, Gutekunst SB, Biastoch A (2020) A near-synoptic survey of ocean microplastic concentration along an around-the-world sailing race. PLOS ONE 15(12): e0243203. https://doi.org/10.1371/journal.pone.0243203 \| \| Tata, T., Belabed, B.E., Bououdina, M. and Bellucci, S., 2020. Occurrence and characterization of surface sediment microplastics and litter from North African coasts of Mediterranean Sea: Preliminary research and first evidence. Science of the total environment, 713, p.136664. \| \| Tekman, M.B., Wekerle, C., Lorenz, C., Primpke, S., Hasemann, C., Gerdts, G. and Bergmann, M., 2020. Tying up loose ends of microplastic pollution in the Arctic: distribution from the sea surface through the water column to deep-sea sediments at the HAUSGARTEN Observatory. Environmental science & technology, 54(7), pp.4079-4090. \| \| Terzi, Y., Gedik, K., Eryaşar, A.R., Öztürk, R.Ç., Şahin, A. and Yılmaz, F., 2022. Microplastic contamination and characteristics spatially vary in the southern Black Sea beach sediment and sea surface water. Marine pollution bulletin, 174, p.113228. \| \| Tiwari, M., Rathod, T.D., Ajmal, P.Y., Bhangare, R.C. and Sahu, S.K., 2019. Distribution and characterization of microplastics in beach sand from three different Indian coastal environments. Marine pollution bulletin, 140, pp.262-273. \| \| Truchet, D.M., López, A.F., Ardusso, M.G., Rimondino, G.N., Buzzi, N.S., Malanca, F.E., Spetter, C.V. and Severini, M.F., 2021. Microplastics in bivalves, water and sediments from a touristic sandy beach of Argentina. Marine Pollution Bulletin, 173, p.113023. \| \| Tsang, Y.Y., Mak, C.W., Liebich, C., Lam, S.W., Sze, E.T. and Chan, K.M., 2017. Microplastic pollution in the marine waters and sediments of Hong Kong. Marine Pollution Bulletin, 115(1-2), pp.20-28. \| \| Tsang, Y.Y., Mak, C.W., Liebich, C., Lam, S.W., Sze, E.T.P. and Chan, K.M., 2020. Spatial and temporal variations of coastal microplastic pollution in Hong Kong. Marine Pollution Bulletin, 161, p.111765. \| \| Tsukada, E., Fernandes, E., Vidal, C. and Salla, R.F., 2021. Beach morphodynamics and its relationship with the deposition of plastic particles: A preliminary study in southeastern Brazil. Marine pollution bulletin, 172, p.112809. \| \| Tziourrou, P., Megalovasilis, P., Tsounia, M. and Karapanagioti, H.K., 2019. Characteristics of microplastics on two beaches affected by different land uses in Salamina Island in Saronikos Gulf, east Mediterranean. Marine pollution bulletin, 149, p.110531. \| \| Urban-Malinga, B., Zalewski, M., Jakubowska, A., Wodzinowski, T., Malinga, M., Pałys, B. and Dąbrowska, A., 2020. Microplastics on sandy beaches of the southern Baltic Sea. Marine Pollution Bulletin, 155, p.111170. \| \| Utami, D. A., Reuning, L., Konechnaya, O., & Schwarzbauer, J. (2021). Microplastics as a sedimentary component in reef systems: a case study from the Java Sea. *Sedimentology*, *68*(6), 2270-2292. \| \| van Cauwenberghe, L., Vanreusel, A., Mees, J., Janssen, C.R., 2013. Microplastic pollution in deep-sea sediments. Environ. Pollut. 182, 495–499 \| \| Veerasingam, S., Saha, M., Suneel, V., Vethamony, P., Rodrigues, A.C., Bhattacharyya, S. and Naik, B.G., 2016. Characteristics, seasonal distribution and surface degradation features of microplastic pellets along the Goa coast, India. Chemosphere, 159, pp.496-505. \| \| Veerasingam, S., Vethamony, P., Aboobacker, V.M., Giraldes, A.E., Dib, S. and Al-Khayat, J.A., 2021. Factors influencing the vertical distribution of microplastics in the beach sediments around the Ras Rakan Island, Qatar. Environmental Science and Pollution Research, pp.1-10. \| \| Vetrimurugan, E., Jonathan, M.P., Sarkar, S.K., Rodríguez-González, F., Roy, P.D., Velumani, S. and Sakthi, J.S., 2020. Occurrence, distribution and provenance of micro plastics: A large scale quantitative analysis of beach sediments from southeastern coast of South Africa. Science of the Total Environment, 746, p.141103. \| \| Vianello, A., Boldrin, A., Guerriero, P., Moschino, V., Rella, R., Sturaro, A. and Da Ros, L., 2013. Microplastic particles in sediments of Lagoon of Venice, Italy: First observations on occurrence, spatial patterns and identification. Estuarine, Coastal and Shelf Science, 130, pp.54-61. \| \| Villanova-Solano, C., Díaz-Peña, F.J., Hernández-Sánchez, C., González-Sálamo, J., González-Pleiter, M., Vega-Moreno, D., Fernández-Piñas, F., Fraile-Nuez, E., Machín, F. and Hernández-Borges, J., 2022. Microplastic pollution in sublittoral coastal sediments of a North Atlantic island: The case of La Palma (Canary Islands, Spain). Chemosphere, 288, p.132530. \| \| Wang, D., Su, L., Ruan, H.D., Chen, J., Lu, J., Lee, C.H. and Jiang, S.Y., 2021. Quantitative and qualitative determination of microplastics in oyster, seawater and sediment from the coastal areas in Zhuhai, China. Marine Pollution Bulletin, 164, p.112000. \| \| Wang, J., Wang, M., Ru, S. and Liu, X., 2019. High levels of microplastic pollution in the sediments and benthic organisms of the South Yellow Sea, China. Science of the Total Environment, 651, pp.1661-1669. \| \| Wang, T., Hu, M., Song, L., Yu, J., Liu, R., Wang, S., Wang, Z., Sokolova, I.M., Huang, W. and Wang, Y., 2020. Coastal zone use influences the spatial distribution of microplastics in Hangzhou Bay, China. Environmental Pollution, 266, p.115137. \| \| Wang, T., Li, B., Yu, W. and Zou, X., 2021. Microplastic pollution and quantitative source apportionment in the Jiangsu coastal area, China. Marine Pollution Bulletin, 166, p.112237. \| \| Wang, X. P., Lo Hoi, S., Fu, Y., Wu, Z., Qin, D., Huang, X., Zhu, J., Cheung Siu, G. & Kwan Kit, Y. (2022). "High Microplastic Contamination in Juvenile Tri-Spine Horseshoe Crabs: A Baseline Study of Nursery Habitats in Northern Beibu Gulf, China." JOURNAL OF OCEAN UNIVERSITY OF CHINA 21(3): 521-530. \| \| Wang, Y., Nakano, H., Xu, H. and Arakawa, H., 2021. Contamination of seabed sediments in Tokyo Bay by small microplastic particles. Estuarine, Coastal and Shelf Science, 261, p.107552. \| \| Wang, Y., Zou, X., Peng, C., Qiao, S., Wang, T., Yu, W., Khokiattiwong, S. and Kornkanitnan, N., 2020. Occurrence and distribution of microplastics in surface sediments from the Gulf of Thailand. Marine Pollution Bulletin, 152, p.110916. \| \| Weitzel, S.L., 2020. Wintering Population Estimates and Microplastics Prevalence for Tidal Marsh Birds of Mississippi. Mississippi State University. \| \| Weitzel, S.L., Feura, J.M., Rush, S.A., Iglay, R.B. and Woodrey, M.S., 2021. Availability and assessment of microplastic ingestion by marsh birds in Mississippi Gulf Coast tidal marshes. Marine Pollution Bulletin, 166, p.112187. \| \| Wertz, H., 2015. Marine debris in Charleston Harbor: Characterizing plastic particles in the field and assessing their effects on juvenile clams (Mercenaria mercenaria). College of Charleston. \| \| Willis, K.A., Eriksen, R., Wilcox, C. and Hardesty, B.D., 2017. Microplastic distribution at different sediment depths in an urban estuary. Frontiers in Marine Science, 4, p.419. \| \| Wilson, D.R., Godley, B.J., Haggar, G.L., Santillo, D. and Sheen, K.L., 2021. The influence of depositional environment on the abundance of microplastic pollution on beaches in the Bristol Channel, UK. Marine Pollution Bulletin, 164, p.111997. \| \| Woodall, L.C., Sanchez-Vidal, A., Canals, M., Paterson, G.L., Coppock, R., Sleight, V., Calafat, A., Rogers, A.D., Narayanaswamy, B.E. and Thompson, R.C., 2014. The deep sea is a major sink for microplastic debris. Royal Society open science, 1(4), p.140317. \| \| Wu, F., Pennings, S.C., Tong, C. and Xu, Y., 2020. Variation in microplastics composition at small spatial and temporal scales in a tidal flat of the Yangtze Estuary, China. Science of The Total Environment, 699, p.134252. \| \| Wu, N., Zhang, Y., Li, W., Wang, J., Zhang, X., He, J., Li, J., Ma, Y. and Niu, Z., 2020. Co-effects of biofouling and inorganic matters increased the density of environmental microplastics in the sediments of Bohai Bay coast. Science of The Total Environment, 717, p.134431. \| \| Wu, X., Zhong, C., Wang, T., Zou, X., Zang, Z., Li, Q. and Chen, H., 2021. Occurrence and distribution of microplastics on recreational beaches of Haichow Bay, China. Environmental Science and Pollution Research, 28(5), pp.6132-6145. \| \| Wu, Y. L., Chen, X., Wen, L., Li, Z., Peng, M., Wu, H. & Xie, L. (2022). "Linking human activity to spatial accumulation of microplastics along mangrove coasts." Science of The Total Environment 825. \| \| Xu, L., Cao, L., Huang, W., Liu, J. and Dou, S., 2021. Assessment of plastic pollution in the Bohai Sea: Abundance, distribution, morphological characteristics and chemical components. Environmental Pollution, 278, p.116874. \| \| Xue, B., Zhang, L., Li, R., Wang, Y., Guo, J., Yu, K. and Wang, S., 2020. Underestimated microplastic pollution derived from fishery activities and “hidden” in deep sediment. Environmental science & technology, 54(4), pp.2210-2217. \| \| Yabanlı, M., Yozukmaz, A., Şener, İ. and Ölmez, Ö.T., 2019. Microplastic pollution at the intersection of the Aegean and Mediterranean Seas: A study of the Datça Peninsula (Turkey). Marine pollution bulletin, 145, pp.47-55. \| \| Yao, W., Di, D., Wang, Z., Liao, Z., Huang, H., Mei, K., Dahlgren, R.A., Zhang, M. and Shang, X., 2019. Micro-and macroplastic accumulation in a newly formed Spartina alterniflora colonized estuarine saltmarsh in southeast China. Marine Pollution Bulletin, 149, p.110636. \| \| Yaranal, N.A., Subbiah, S. and Mohanty, K., 2021. Distribution and characterization of microplastics in beach sediments from Karnataka (India) coastal environments. Marine Pollution Bulletin, 169, p.112550. \| \| Yona, D., Sari, S.H.J., Iranawati, F., Bachri, S. and Ayuningtyas, W.C., 2019. Microplastics in the surface sediments from the eastern waters of Java Sea, Indonesia. F1000Research, 8. \| \| Yoswaty, D., Amin, B., Fatwa, E.B. and Pakpahan, D., 2021, February. Identification of microplastic waste in sea water, sediment in the sea waters of Dumai City, Riau Province. In IOP Conference Series: Earth and Environmental Science (Vol. 674, No. 1, p. 012113). IOP Publishing. \| \| Yu, L. Y., Li, R.L., Zhang, Z., Wu, H.L., Chai, M.W., Zhu, X.S. & Guo, W.X. (2022). "Distribution, characteristics, and human exposure to microplastics in mangroves within the Guangdong-Hong Kong-Macao Greater Bay Area." Marine Pollution Bulletin 175. \| \| Yu, X., Ladewig, S., Bao, S., Toline, C.A., Whitmire, S. and Chow, A.T., 2018. Occurrence and distribution of microplastics at selected coastal sites along the southeastern United States. Science of the Total Environment, 613, pp.298-305. \| \| Yu, X., Peng, J., Wang, J., Wang, K. and Bao, S., 2016. Occurrence of microplastics in the beach sand of the Chinese inner sea: the Bohai Sea. Environmental pollution, 214, pp.722-730. \| \| Zamani, N.P. and Ismet, M.S., 2021, May. The distribution of marine debris and microplastic in Tidung Kecil Island, Jakarta Bay and Sembilang National Park, Palembang. In IOP Conference Series: Earth and Environmental Science (Vol. 771, No. 1, p. 012038). IOP Publishing. \| \| Zamprogno, G.C., Caniçali, F.B., dos Reis Cozer, C., Otegui, M.B.P., Graceli, J.B. and da Costa, M.B., 2021. Spatial distribution of microplastics in the superficial sediment of a mangrove in Southeast Brazil: A comparison between fringe and basin. Science of The Total Environment, 784, p.146963. \| \| Zhang, B., Wu, D., Yang, X., Teng, J., Liu, Y., Zhang, C., Zhao, J., Yin, X., You, L., Liu, Y. and Wang, Q., 2019. Microplastic pollution in the surface sediments collected from Sishili Bay, North Yellow Sea, China. Marine pollution bulletin, 141, pp.9-15. \| \| Zhang, C., Zhou, H., Cui, Y., Wang, C., Li, Y. and Zhang, D., 2019. Microplastics in offshore sediment in the yellow Sea and east China Sea, China. Environmental Pollution, 244, pp.827-833. \| \| Zhang, D., Liu, X., Huang, W., Li, J., Wang, C., Zhang, D. and Zhang, C., 2020. Microplastic pollution in deep-sea sediments and organisms of the Western Pacific Ocean. Environmental Pollution, 259, p.113948. \| \| Zhang, L., Zhang, S., Guo, J., Yu, K., Wang, Y. and Li, R., 2020. Dynamic distribution of microplastics in mangrove sediments in Beibu Gulf, South China: Implications of tidal current velocity and tidal range. Journal of Hazardous Materials, 399, p.122849. \| \| Zhang, T., Lin, L., Li, D., Wu, S., Kong, L., Wang, J. and Shi, H., 2021. The microplastic pollution in beaches that served as historical nesting grounds for green turtles on Hainan Island, China. Marine Pollution Bulletin, 173, p.113069. \| \| Zhang, X., Xia, X., Dai, M., Cen, J., Zhou, L. and Xie, J., 2021. Microplastic pollution and its relationship with the bacterial community in coastal sediments near Guangdong Province, South China. Science of The Total Environment, 760, p.144091. \| \| Zhao, J., Ran, W., Teng, J., Liu, Y., Liu, H., Yin, X., Cao, R. and Wang, Q., 2018. Microplastic pollution in sediments from the Bohai Sea and the Yellow Sea, China. Science of the Total Environment, 640, pp.637-645. \| \| Zhao, S., Zhu, L. and Li, D., 2015. Characterization of small plastic debris on tourism beaches around the South China Sea. Regional Studies in Marine Science, 1, pp.55-62. \| \| Zheng, Y., Li, J., Cao, W., Jiang, F., Zhao, C., Ding, H., Wang, M., Gao, F. and Sun, C., 2020. Vertical distribution of microplastics in bay sediment reflecting effects of sedimentation dynamics and anthropogenic activities. Marine Pollution Bulletin, 152, p.110885. \| \| Zheng, Y., Li, J., Cao, W., Liu, X., Jiang, F., Ding, J., Yin, X. and Sun, C., 2019. Distribution characteristics of microplastics in the seawater and sediment: a case study in Jiaozhou Bay, China. Science of the Total Environment, 674, pp.27-35. \| \| Zhou, Q., Tu, C., Fu, C., Li, Y., Zhang, H., Xiong, K., Zhao, X., Li, L., Waniek, J.J. and Luo, Y., 2020. Characteristics and distribution of microplastics in the coastal mangrove sediments of China. *Science of The Total Environment*, *703*, p.134807. \| \| Zhou, Q., Zhang, H., Fu, C., Zhou, Y., Dai, Z., Li, Y., Tu, C. and Luo, Y., 2018. The distribution and morphology of microplastics in coastal soils adjacent to the Bohai Sea and the Yellow Sea. Geoderma, 322, pp.201-208. \| \| Zhu, L., Bai, H., Chen, B., Sun, X., Qu, K. and Xia, B., 2018. Microplastic pollution in North Yellow Sea, China: Observations on occurrence, distribution and identification. Science of the Total Environment, 636, pp.20-29. \| \| Zhu, X., Ran, W., Teng, J., Zhang, C., Zhang, W., Hou, C., Zhao, J., Qi, X. and Wang, Q., 2021. Microplastic pollution in nearshore sediment from the Bohai Sea coastline. Bulletin of Environmental Contamination and Toxicology, 107(4), pp.665-670. \| \| Zobkov, M. and Esiukova, E., 2017. Microplastics in Baltic bottom sediments: quantification procedures and first results. Marine pollution bulletin, 114(2), pp.724-732. \| \| Zuo, L., Sun, Y., Li, H., Hu, Y., Lin, L., Peng, J. and Xu, X., 2020. Microplastics in mangrove sediments of the Pearl River Estuary, South China: Correlation with halogenated flame retardants' levels. Science of The Total Environment, 725, p.138344. \| |
| --- | --- | --- | --- | --- | --- | --- | --- | --- | --- | --- | --- | --- | --- | --- | --- | --- | --- | --- | --- | --- | --- | --- | --- | --- | --- | --- | --- | --- | --- | --- | --- | --- | --- | --- | --- | --- | --- | --- | --- | --- | --- | --- | --- | --- | --- | --- | --- | --- | --- | --- | --- | --- | --- | --- | --- | --- | --- | --- | --- | --- | --- | --- | --- | --- | --- | --- | --- | --- | --- | --- | --- | --- | --- | --- | --- | --- | --- | --- | --- | --- | --- | --- | --- | --- | --- | --- | --- | --- | --- | --- | --- | --- | --- | --- | --- | --- | --- | --- | --- | --- | --- | --- | --- | --- | --- | --- | --- | --- | --- | --- | --- | --- | --- | --- | --- | --- | --- | --- | --- | --- | --- | --- | --- | --- | --- | --- | --- | --- | --- | --- | --- | --- | --- | --- | --- | --- | --- | --- | --- | --- | --- | --- | --- | --- | --- | --- | --- | --- | --- | --- | --- | --- | --- | --- | --- | --- | --- | --- | --- | --- | --- | --- | --- | --- | --- | --- | --- | --- | --- | --- | --- | --- | --- | --- | --- | --- | --- | --- | --- | --- | --- | --- | --- | --- | --- | --- | --- | --- | --- | --- | --- | --- | --- | --- | --- | --- | --- | --- | --- | --- | --- | --- | --- | --- | --- | --- | --- | --- | --- | --- | --- | --- | --- | --- | --- | --- | --- | --- | --- | --- | --- | --- | --- | --- | --- | --- | --- | --- | --- | --- | --- | --- | --- | --- | --- | --- | --- | --- | --- | --- | --- | --- | --- | --- | --- | --- | --- | --- | --- | --- | --- | --- | --- | --- | --- | --- | --- | --- | --- | --- | --- | --- | --- | --- | --- | --- | --- | --- | --- | --- | --- | --- | --- | --- | --- | --- | --- | --- | --- | --- | --- | --- | --- | --- | --- | --- | --- | --- | --- | --- | --- | --- | --- | --- | --- | --- | --- | --- | --- | --- | --- | --- | --- | --- | --- | --- | --- | --- | --- | --- | --- | --- | --- | --- | --- | --- | --- | --- | --- | --- | --- | --- | --- | --- | --- | --- | --- | --- | --- | --- | --- | --- | --- | --- | --- | --- | --- | --- | --- |

**Dry Bulk Density references**

| Andersen, T. J., Lund-Hansen, L. C., Pejrup, M., Jensen, K. T., & Mouritsen, K. N. (2005). Biologically induced differences in erodibility and aggregation of subtidal and intertidal sediments: a possible cause for seasonal changes in sediment deposition. *Journal of Marine Systems*, *55*(3-4), 123-138. |
| --- |
| Arias-Ortiz, A., Masqué, P., Glass, L., Benson, L., Kennedy, H., Duarte, C. M., ... & Lovelock, C. E. (2021). Losses of soil organic carbon with deforestation in mangroves of Madagascar. *Ecosystems*, *24*, 1-19. |
| Aspen, R. J., Vardy, S., Perkins, R. G., Davidson, I. R., Bates, R., & Paterson, D. M. (2004). The effects of clam fishing on the properties of surface sediments in the lagoon of Venice, Italy. *Hydrology and Earth System Sciences*, *8*(2), 160-169. |
| Bai, J., Xiao, R., Zhang, K., & Gao, H. (2012). Arsenic and heavy metal pollution in wetland soils from tidal freshwater and salt marshes before and after the flow-sediment regulation regime in the Yellow River Delta, China. *Journal of Hydrology*, *450*, 244-253. |
| Barry, S. C., Bianchi, T. S., Shields, M. R., Hutchings, J. A., Jacoby, C. A., & Frazer, T. K. (2018). Characterizing blue carbon stocks in Thalassia testudinum meadows subjected to different phosphorus supplies: A lignin biomarker approach. *Limnology and Oceanography*, *63*(6), 2630-2646. |
| Bartholdy, J., Bartholdy, A. T., Kim, D., & Pedersen, J. B. T. (2014). On autochthonous organic production and its implication for the consolidation of temperate salt marshes. *Marine Geology*, *351*, 53-57. |
| Bartholdy, J., Pedersen, J. B., & Bartholdy, A. T. (2010). Autocompaction of shallow silty salt marsh clay. *Sedimentary Geology*, *223*(3-4), 310-319. |
| Berthold, M., Zimmer, D., Reiff, V., & Schumann, R. (2018). Phosphorus contents re-visited after 40 years in muddy and sandy sediments of a temperate lagoon system. *Frontiers in Marine Science*, *5*, 305. |
| Boto, K. G., & Wellington, J. T. (1984). Soil characteristics and nutrient status in a northern Australian mangrove forest. *Estuaries*, *7*, 61-69. |
| Breithaupt, J. L., Smoak, J. M., Rivera-Monroy, V. H., Castañeda-Moya, E., Moyer, R. P., Simard, M., & Sanders, C. J. (2017). Partitioning the relative contributions of organic matter and mineral sediment to accretion rates in carbonate platform mangrove soils. *Marine Geology*, *390*, 170-180. |
| Bryant, J. C., & Chabreck, R. H. (1998). Effects of impoundment on vertical accretion of coastal marsh. *Estuaries*, *21*, 416-422. |
| Cacho, C. V., Conrad, S. R., Brown, D. R., Riggs, A., Gardner, K., Li, L., ... & Sanders, C. J. (2021). Local geomorphological gradients affect sedimentary organic carbon storage: A Blue Carbon case study from sub-tropical Australia. *Regional Studies in Marine Science*, *45*, 101840. |
| Callaway, J. C. (1994). Sedimentation processes in selected coastal wetlands from the Gulf of Mexico and Northern Europe. Louisiana State University and Agricultural & Mechanical College. |
| Cardona, P., & Botero, L. (1998). Soil characteristics and vegetation structure in a heavily deteriorated mangrove forest in the Caribbean coast of Colombia. *Biotropica*, *30*(1), 24-34. |
| Carter, L., Neil, H. L., & McCave, I. N. (2000). Glacial to interglacial changes in non-carbonate and carbonate accumulation in the SW Pacific Ocean, New Zealand. *Palaeogeography, Palaeoclimatology, Palaeoecology*, *162*(3-4), 333-356. |
| Celis-Hernández, O., Ávila, E., Ward, R.D., Rodríguez-Santiago, M.A. and Aguirre-Téllez, J.A., 2021. Microplastic distribution in urban vs pristine mangroves: Using marine sponges as bioindicators of environmental pollution. Environmental Pollution, 284, p.117391. |
| Cozzolino, L., Nicastro, K.R., Zardi, G.I. and Carmen, B., 2020. Species-specific plastic accumulation in the sediment and canopy of coastal vegetated habitats. Science of The Total Environment, 723, p.138018. |
| Dahl, M., Deyanova, D., Gütschow, S., Asplund, M. E., Lyimo, L. D., Karamfilov, V., ... & Gullström, M. (2016). Sediment characteristics as an important factor for revealing carbon storage in Zostera marina meadows: A comparison of four European areas. *Biogeosciences Discussions*, *2016*, 1-30. |
| Delafontaine, M. T., Barrtholomä, A., & Flemming, B. W. (1997). Volume-specific dry POC mass in surficial intertidal sediments: a comparison between biogenic muds and adjacent sand flats. |
| Delafontaine, M. T., Bartholoma, A., Flemming, B. W. & Kurmis, R (1997). Volume-specific dry POC mass in surficial intertidal sediments: a comparison between biogenic muds and adjacent sand flats. Senckenbergiana Maritima |
| Doyle, M. O., & Otte, M. L. (1997). Organism-induced accumulation of iron, zinc and arsenic in wetland soils. *Environmental Pollution*, *96*(1), 1-11. |
| El Hussieny, S. A., Shaltout, K. H., & Alatar, A. A. (2021). Carbon sequestration potential of Avicennia marina (Forssk.) Vierh. and Rhizophora mucronata Lam. along the Western Red Sea Coast of Egypt. *Rendiconti Lincei. Scienze Fisiche e Naturali*, *32*, 599-607. |
| Etemadi, H., Smoak, J. M., & Sanders, C. J. (2018). Forest migration and carbon sources to Iranian mangrove soils. *Journal of Arid Environments*, *157*, 57-65. |
| Fourqurean, J. W., Kendrick, G. A., Collins, L. S., Chambers, R. M., & Vanderklift, M. A. (2012). Carbon, nitrogen and phosphorus storage in subtropical seagrass meadows: examples from Florida Bay and Shark Bay. *Marine and Freshwater Research*, *63*(11), 967-983. |
| Gallagher, J. B., Chew, S. T., Madin, J., & Thorhaug, A. (2020). Valuing carbon stocks across a tropical lagoon after accounting for black and inorganic carbon: bulk density proxies for monitoring. *Journal of Coastal Research*, *36*(5), 1029-1039. |
| Ganguly, D., Singh, G., Ramachandran, P., Selvam, A. P., Banerjee, K., & Ramachandran, R. (2017). Seagrass metabolism and carbon dynamics in a tropical coastal embayment. *Ambio*, *46*, 667-679. |
| Gorsline, D. S. (1992). The geological setting of Santa Monica and San Pedro basins, California continental borderland. *Progress in Oceanography*, *30*(1-4), 1-36. |
| Gwo, W. H., & Yu-Chia, C. (1994). Sedimentation rates on the continental slope off eastern Taiwan. *Marine geology*, *119*(1-2), 99-109. |
| Harttung, S. A., Radabaugh, K. R., Moyer, R. P., Smoak, J. M., & Chambers, L. G. (2021). Coastal riverine wetland biogeochemistry follows soil organic matter distribution along a marsh-to-mangrove gradient (Florida, USA). *Science of The Total Environment*, *797*, 149056. |
| Hay, R., & Flemming, B. W. (1984). On the bulk density of South African marine sands. *South African Journal of Geology*, *87*(3), 233-236. |
| Hien, H. T., Marchand, C., Aime, J., & Cuc, N. T. K. (2018). Seasonal variability of CO2 emissions from sediments in planted mangroves (Northern Viet Nam). *Estuarine, Coastal and Shelf Science*, *213*, 28-39. |
| Huang, L., Bai, J., Chen, B., Zhang, K., Huang, C., & Liu, P. (2012). Two-decade wetland cultivation and its effects on soil properties in salt marshes in the Yellow River Delta, China. *Ecological Informatics*, *10*, 49-55. |
| Huang, X., Wang, X., Li, X., Xin, K., Yan, Z., Sun, Y., & Bellerby, R. (2018). Distribution pattern and influencing factors for soil organic carbon (SOC) in mangrove communities at Dongzhaigang, China. *Journal of Coastal Research*, *34*(2), 434-442. |
| Huang, Y., Xiao, X., Effiong, K., Xu, C., Su, Z., Hu, J., Jiao, S. and Holmer, M., 2021. New Insights into the Microplastic Enrichment in the Blue Carbon Ecosystem: Evidence from Seagrass Meadows and Mangrove Forests in Coastal South China Sea. Environmental Science & Technology, 55(8), pp.4804-4812. |
| Huang, Y., Xiao, X., Xu, C., Perianen, Y.D., Hu, J. and Holmer, M., 2020. Seagrass beds acting as a trap of microplastics-Emerging hotspot in the coastal region?. Environmental Pollution, 257, p.113450. |
| Jiang, Z., Liu, S., Zhang, J., Zhao, C., Wu, Y., Yu, S., ... & Kumar, M. (2017). Newly discovered seagrass beds and their potential for blue carbon in the coastal seas of Hainan Island, South China Sea. Marine pollution bulletin, 125(1-2), 513-521. |
| Joensuu, M., Pilditch, C. A., & Norkko, A. (2020). Temporal variation in resuspension potential and associated nutrient dynamics in shallow coastal environments. *Estuaries and Coasts*, *43*, 1361-1376. |
| Jumprom, N., Rodcharoen, E., & Wichachucherd, B. (2021). The Physicochemical Properties of Sediment and Ground Cover for a Secondary Mangrove System in Thailand. *Journal of Coastal Research*, *37*(4), 784-792. |
| Kaladharan, P., Vijayakumaran, K., Edward, L., Lavanya, R., & Dash, B. (2021). Blue carbon stock of seagrass meadows of Chilika and Pulicat Lakes along the Eastern coast of India. *Fishery Technology*, *58*, 143-146. |
| Keller, J. A., Wilson Grimes, K., Reeve, A. S., & Platenberg, R. (2017). Mangroves buffer marine protected area from impacts of Bovoni Landfill, St. Thomas, United States Virgin Islands. *Wetlands Ecology and Management*, *25*, 563-582. |
| Kim, S. H., Suonan, Z., Qin, L. Z., Kim, H., Park, J. I., Kim, Y. K., ... & Lee, K. S. (2022). Variability in blue carbon storage related to biogeochemical factors in seagrass meadows off the coast of the Korean peninsula. *Science of The Total Environment*, *813*, 152680. |
| Kongchum, M., Devai, I., DeLaune, R. D., & Jugsujinda, A. (2006). Total mercury and methylmercury in freshwater and salt marsh soils of the Mississippi river deltaic plain. *Chemosphere*, *63*(8), 1300-1303. |
| Kreitsberg, R., Raudna-Kristoffersen, M., Heinlaan, M., Ward, R., Visnapuu, M., Kisand, V., Meitern, R., Kotta, J. and Tuvikene, A., 2021. Seagrass beds reveal high abundance of microplastic in sediments: A case study in the Baltic Sea. *Marine Pollution Bulletin*, *168*, p.112417. |
| Kusakabe, M., Oikawa, S., Takata, H., & Misonoo, J. (2013). Spatiotemporal distributions of Fukushima-derived radionuclides in nearby marine surface sediments. *Biogeosciences*, *10*(7), 5019-5030. |
| Leipe, T., Tauber, F., Vallius, H., Virtasalo, J., Uścinowicz, S., Kowalski, N., ... & Myllyvirta, T. (2011). Particulate organic carbon (POC) in surface sediments of the Baltic Sea. *Geo-Marine Letters*, *31*, 175-188. |
| Lima, M. D. A. C., Ward, R. D., & Joyce, C. B. (2020). Environmental drivers of sediment carbon storage in temperate seagrass meadows. *Hydrobiologia*, *847*(7), 1773-1792. |
| Liu, J. P., DeMaster, D. J., Nittrouer, C. A., Eidam, E. F., & Nguyen, T. T. (2017). A seismic study of the Mekong subaqueous delta: Proximal versus distal sediment accumulation. *Continental Shelf Research*, *147*, 197-212. |
| Liu, X., Liu, H., Chen, L. and Wang, X., 2022. Ecological interception effect of mangroves on microplastics. Journal of Hazardous Materials, 423, p.127231. |
| Mariotti, G., Elsey‐Quirk, T., Bruno, G., & Valentine, K. (2020). Mud‐associated organic matter and its direct and indirect role in marsh organic matter accumulation and vertical accretion. *Limnology and Oceanography*, *65*(11), 2627-2641. |
| Martín, J., Puig, P., Masqué, P., Palanques, A., & Sánchez-Gómez, A. (2014). Impact of bottom trawling on deep-sea sediment properties along the flanks of a submarine canyon. *PloS one*, *9*(8), e104536. |
| Matthai, C., Birch, G. F., Jenkinson, A., & Heijnis, H. (2001). Physical resuspension and vertical mixing of sediments on a high energy continental margin (Sydney, Australia). *Journal of Environmental Radioactivity*, *52*(1), 67-89. |
| McAtee, J. W., & Drawe, D. L. (1981). Human impact on beach and foredune microclimate on North Padre Island, Texas. *Environmental Management*, *5*, 121-134. |
| Müller, P. J., & Suess, E. (1979). Productivity, sedimentation rate, and sedimentary organic matter in the oceans—I. Organic carbon preservation. *Deep Sea Research Part A. Oceanographic Research Papers*, *26*(12), 1347-1362. |
| Naidoo, G. (1980). Mangrove soils of the Beachwood area, Durban. J. S. Afr. Bot.. 46. 293-304. |
| Nijenhuis, I. A., Becker, J., & De Lange, G. J. (2001). Geochemistry of coeval marine sediments in Mediterranean ODP cores and a land section: implications for sapropel formation models. *Palaeogeography, Palaeoclimatology, Palaeoecology*, *165*(1-2), 97-112. |
| Nolte, S., Müller, F., Schuerch, M., Wanner, A., Esselink, P., Bakker, J. P., & Jensen, K. (2013). Does livestock grazing affect sediment deposition and accretion rates in salt marshes?. *Estuarine, Coastal and Shelf Science*, *135*, 296-305. |
| Panyawai, J., Tuntiprapas, P., & Prathep, A. (2019). High macrophyte canopy complexity enhances sediment retention and carbon storage in coastal vegetative meadows at Tangkhen Bay, Phuket, Southern Thailand. *Ecological research*, *34*(1), 201-212. |
| Paradis, S., Pusceddu, A., Masqué, P., Puig, P., Moccia, D., Russo, T., & Lo Iacono, C. (2019). Organic matter contents and degradation in a highly trawled area during fresh particle inputs (Gulf of Castellammare, southwestern Mediterranean). *Biogeosciences*, *16*(21), 4307-4320. |
| Pérez, A., Machado, W., Gutiérrez, D., Borges, A. C., Patchineelam, S. R., & Sanders, C. J. (2018). Carbon accumulation and storage capacity in mangrove sediments three decades after deforestation within a eutrophic bay. *Marine pollution bulletin*, *126*, 275-280. |
| Potouroglou, M., Whitlock, D., Milatovic, L., MacKinnon, G., Kennedy, H., Diele, K., & Huxham, M. (2021). The sediment carbon stocks of intertidal seagrass meadows in Scotland. *Estuarine, Coastal and Shelf Science*, *258*, 107442. |
| Rabouille, C., Stahl, H., Bassinot, F., Tengberg, A., Brunnegard, J., Hall, P., ... & Lampitt, R. S. (2001). Imbalance in the carbonate budget of surficial sediments in the North Atlantic Ocean: variations over the last millenium?. *Progress in Oceanography*, *50*(1-4), 201-221. |
| Radabaugh, K. R., Dontis, E. E., Chappel, A. R., Russo, C. E., & Moyer, R. P. (2021). Early indicators of stress in mangrove forests with altered hydrology in Tampa Bay, Florida, USA. *Estuarine, Coastal and Shelf Science*, *254*, 107324. |
| Rattanachot, E., & Prathep, A. (2015). Species specific effects of three morphologically different belowground seagrasses on sediment properties. *Estuarine, Coastal and Shelf Science*, *167*, 427-435. |
| Román-Sierra, J., Muñoz-Perez, J. J., & Navarro-Pons, M. (2014). Beach nourishment effects on sand porosity variability. *Coastal Engineering*, *83*, 221-232. |
| Rosencranz, J. A., Brown, L. N., Holmquist, J. R., Sanchez, Y., MacDonald, G. M., & Ambrose, R. F. (2017). The role of sediment dynamics for inorganic accretion patterns in southern California’s Mediterranean-climate salt marshes. *Estuaries and Coasts*, *40*, 1371-1384. |
| Ruranska, P. ; Ladd, C.J.T.; Smeaton, C.; Skov, M.W.; Austin, W.E.N. (2022). Dry bulk density, loss on ignition and organic carbon content of surficial soils from English and Welsh salt marshes 2019 NERC EDS Environmental Information Data Centre. https://doi.org/10.5285/e5554b83-910f-4030-8f4e-81967dc7047c |
| Ruranska, P., Miller, L. C., Hindle, C., Ladd, C. J. T., Smeaton, C., & Skov, M. W. (2020). Dry bulk density, loss on ignition and organic carbon content of surficial soils from Scottish salt marshes 2018-2019. |
| Samper-Villarreal, J., Mumby, P. J., Saunders, M. I., Barry, L. A., Zawadzki, A., Heijnis, H., ... & Lovelock, C. E. (2018). Vertical accretion and carbon burial rates in subtropical seagrass meadows increased following anthropogenic pressure from European colonisation. *Estuarine, Coastal and Shelf Science*, *202*, 40-53. |
| Sánchez-Núñez, D. A., Bernal, G., & Mancera Pineda, J. E. (2019). The relative role of mangroves on wave erosion mitigation and sediment properties. *Estuaries and Coasts*, *42*, 2124-2138. |
| Sanders, C. J., Smoak, J. M., Naidu, A. S., Araripe, D. R., Sanders, L. M., & Patchineelam, S. R. (2010). Mangrove forest sedimentation and its reference to sea level rise, Cananeia, Brazil. *Environmental Earth Sciences*, *60*, 1291-1301. |
| Schindler, M., Karius, V., Deicke, M., & von Eynatten, H. (2014). Measuring sediment deposition and accretion on anthropogenic marshland–Part I: Methodical evaluation and development. *Estuarine, Coastal and Shelf Science*, *151*, 236-245. |
| Serrano, O., Mateo, M. A., Renom, P., & Julià, R. (2012). Characterization of soils beneath a Posidonia oceanica meadow. *Geoderma*, *185*, 26-36. |
| Shaltout, K. H., Ahmed, M. T., Alrumman, S. A., Ahmed, D. A., & Eid, E. M. (2020). Evaluation of the carbon sequestration capacity of arid mangroves along nutrient availability and salinity gradients along the Red Sea coastline of Saudi Arabia. *Oceanologia*, *62*(1), 56-69. |
| Shi, C., Zhang, D. D., & You, L. (2003). Sediment budget of the Yellow River delta, China: the importance of dry bulk density and implications to understanding of sediment dispersal. *Marine Geology*, *199*(1-2), 13-25. |
| Smeaton, C., Barlow, N. L., & Austin, W. E. (2020). Coring and compaction: Best practice in blue carbon stock and burial estimations. *Geoderma*, *364*, 114180. |
| St. Laurent, K. A., Hribar, D. J., Carlson, A. J., Crawford, C. M., & Siok, D. (2020). Assessing coastal carbon variability in two Delaware tidal marshes. *Journal of Coastal Conservation*, *24*, 1-16. |
| Steinitz, M. J., Salmon, M., & Wyneken, J. (1998). Beach renourishment and loggerhead turtle reproduction: a seven year study at Jupiter Island, Florida. *Journal of Coastal Research*, 1000-1013. |
| Stephens, J. A., Uncles, R. J., Barton, M. L., & Fitzpatrick, F. (1992). Bulk properties of intertidal sediments in a muddy, macrotidal estuary. *Marine Geology*, *103*(1-3), 445-460. |
| Toscano, M. A., Gonzalez, J. L., & Whelan, K. R. (2018). Calibrated density profiles of Caribbean mangrove peat sequences from computed tomography for assessment of peat preservation, compaction, and impacts on sea-level reconstructions. *Quaternary Research*, *89*(1), 201-222. |
| Ukpong, I. E. (1995). Mangrove soils of the creek town creek/calabar river swamp, southeastern nigeria. *Tropical Ecology*, *36*(1), 103-115. |
| Van Regteren, M., Ten Boer, R., Meesters, E. H., & De Groot, A. V. (2017). Biogeomorphic impact of oligochaetes (Annelida) on sediment properties and Salicornia spp. seedling establishment. *Ecosphere*, *8*(7), e01872. |
| van Rijn, L. C., & Barth, R. (2019). Settling and consolidation of soft mud–sand layers. *Journal of Waterway, Port, Coastal, and Ocean Engineering*, *145*(1), 04018028. |
